# Molecular Sensing with Host Systems for Hyperpolarized ^129^Xe

**DOI:** 10.3390/molecules25204627

**Published:** 2020-10-11

**Authors:** Jabadurai Jayapaul, Leif Schröder

**Affiliations:** Molecular Imaging, Leibniz-Forschungsinstitut für Molekulare Pharmakologie (FMP), 13125 Berlin, Germany; jayapaul@fmp-berlin.de

**Keywords:** xenon, Hyper-CEST, supramolecular complexes, biosensors, NMR, MRI

## Abstract

Hyperpolarized noble gases have been used early on in applications for sensitivity enhanced NMR. ^129^Xe has been explored for various applications because it can be used beyond the gas-driven examination of void spaces. Its solubility in aqueous solutions and its affinity for hydrophobic binding pockets allows “functionalization” through combination with host structures that bind one or multiple gas atoms. Moreover, the transient nature of gas binding in such hosts allows the combination with another signal enhancement technique, namely chemical exchange saturation transfer (CEST). Different systems have been investigated for implementing various types of so-called Xe biosensors where the gas binds to a targeted host to address molecular markers or to sense biophysical parameters. This review summarizes developments in biosensor design and synthesis for achieving molecular sensing with NMR at unprecedented sensitivity. Aspects regarding Xe exchange kinetics and chemical engineering of various classes of hosts for an efficient build-up of the CEST effect will also be discussed as well as the cavity design of host molecules to identify a pool of bound Xe. The concept is presented in the broader context of reporter design with insights from other modalities that are helpful for advancing the field of Xe biosensors.

## 1. Introduction

Despite its excellent molecular specificity, NMR has strong limitations for many applications due to its low sensitivity at physiological conditions. The Boltzmann distribution around room temperature yields detectable magnetization for Faraday induction only if the spin densities are relatively high. This limitation is an even greater challenge for MRI and has been an active field of research for a long time. In this regard, the use of hyperpolarization techniques has opened up new possibilities in medical imaging diagnostics [1,2]. These techniques are key contributions to unleash the full analytical and diagnostic potential that is inherent to the NMR signal, particularly in biomedical applications. Conventional contrast agents act as relaxivity agents on the bulk water pool. They often require (a) relative high concentrations (e.g., 10–100 µM for Gd-based *T*_1_ agents) and (b) come with limited specificity. The strong background signal of ubiquitous tissue water is also of concern for certain applications. It can be addressed by detecting nuclei other than the abundant ^1^H. Fluorine (^19^F) NMR has been used in many studies [3] as a background-free option but still does not solve the sensitivity issue with regard to minimum detectable concentrations. The hyperpolarization of various nuclei, incl. ^3^He, ^13^C, ^15^N, ^129^Xe has thus attracted much attention over the recent years.

Hyperpolarized (hp) reporters based on ^13^C and ^15^N detection rely on covalently bound NMR-active atoms. They are used for real-time metabolic imaging to follow conversion of a metabolically active tracer (e.g., ^13^C-labelled pyruvate) for visualizing the Warburg effect in oncology applications [1,4,5] or for visualizing metabolic turnover in the heart [6,7,8]. The noble gases He and Xe, however, have been used in rather different contexts as they are not part of any biologically relevant molecules. Hp ^3^He has been applied early on for diagnostic lung imaging but is increasingly replaced with ^129^Xe for such purposes [9]. Contrary to He, Xe has a decent solubility in aqueous solutions and has thus also been used in structural biology studies to explore cavities in proteins [10]. Moreover, the investigation of porous materials also benefits from Xe NMR studies where the nucleus is sensitive to interactions with the wall material [11]. Thus, despite being an inert gas, ^129^Xe is a valuable diagnostic and biomolecular reporter for exploring its molecular environment [12,13] as it combines high sensitivity with high specificity. Its affinity to certain molecular environments has fostered further applications of hp Xe in which tailored host structures are utilized for detection through supramolecular inclusion complexes. Hp ^129^Xe NMR also displays a broad chemical shift range of about 300 ppm related to xenon’s large polarizable electron cloud. This large chemical shift range of Xe enables sensing of different chemical environments under biological solutions with high sensitivity. Many different bound Xe resonances are clearly separable within this chemical shift range and thus promote simultaneous detection of various Xe. This motivated the so-called “multiplexing” approach in analogy to different dye-based optical imaging [14,15]. Nanocarriers with Xe affinity have been designed to enable sensitivity-enhanced NMR of otherwise undetectable spin density. Such targeted Xe hosts are summarized as functionalized Xe biosensors that were first pursued by the Pines and Wemmer labs [16]. They have evolved over the past 20 years in different designs with constant improvement in sensitivity. A major step was the combination with indirect detection through saturation transfer [17], a concept that had also consequences for optimized host design. 

Xe biosensors have now been implemented for many different biological targets and their molecular properties have been studied under various conditions. As the concept moves towards preclinical applications, certain criteria have been identified to unleash the full potential that this method has shown in in vitro experiments for translation into biomedical diagnostics. This review summarizes aspects regarding the production and handling of hp Xe and addresses the synthetic challenges and strategies for host structures, their functionalization, and the important aspect of Xe binding kinetics for various host systems. It also summarizes which targets have been investigated and how dual reporters have been implemented for cross-validation. A special emphasis is put on providing a comprehensive review that pinpoints the links between different reporter aspects. This should illustrate how reporter design from other modalities might assist the advancement of “functionalized xenon” and how certain parameters of reversibly bound Xe can be tuned through various (chemical) engineering concepts. It also puts the Xe biosensor approach into a broader context of reporter development. Some concluding remarks will also consider the translational potential and recent advancements in associated fields such as Xe MRI in general. 

## 2. Xenon-Host Interactions: The Basis for Functionalized ^129^Xe NMR

As a gas, Xe is a straightforward reporter for investigating cavities in porous materials [11]. Its application range, however, goes far beyond synthetic structures. Various proteins also have an affinity for Xe. Their complexes with the noble gas have been investigated in X-ray crystallography where Xe serves as a heavy element surrogate probe for O_2_ binding sites in gas binding proteins [18,19,20]. The non-polar, inert guest can be a very powerful probe to explore host cavities that are otherwise occupied by guests but that do not yield a useful reporter signal. Together with its large chemical shift range as a consequence of its large polarizable electron cloud [21], Xe is a highly sensitive NMR reporter for cases where transient binding occurs. Identifying suitable host structures is a key step in designing Xe biosensors and various designs have been proposed. Importantly, both the binding affinity and the exchange kinetics are relevant for efficient molecular sensing.

### 2.1. Unspecific Loose Interactions with Proteins 

Generally, X-ray crystallography has been utilized to study protein structures as part of structural biology-based investigations. Intrinsic binding pockets, internal cavities, and channel pores available at proteins are somewhat less accessible or inadequately characterized by conventional X-ray crystallography. Such studies reveal only the protein’s structural information in the solid state without providing any details on the protein dynamics. To investigate the gas binding pockets both in solution and solid state, hp ^129^Xe was utilized as a promising monoatomic probe [22]. Importantly, the usage of hp ^129^Xe was highly beneficial for investigating the protein structure in solution in cases where crystal structures are unknown. Earlier, it was shown that non-specific binding of Xe to cavities did not alter the overall structure of the proteins [23] appreciably; however, the location of the cavity in the protein has an impact on its neighboring chemical environment, e.g., a cavity located near or next to the active site in an enzyme [24,25]. A structural change in proteins or a modulated catalytic activity of an enzyme is achievable depending upon the administered Xe concentration (higher Xe overpressure). Although these Xe-induced structural changes are marginal in general, under certain experimental conditions they can become highly significant [26]. Regarding sensing applications with Xe, the introduction of Xe should therefore not alter the biomolecular host system under investigation such that this could lead to its structural imbalance and loss of functionality. This will greatly hamper the usage of Xe as ‘so-called’ inert monoatomic biomolecular probe for studying the involved dynamics and the availability of binding pockets at proteins in solution and in solid state. Additionally, the Xe occupancy at cavities is often regulated by the employed temperature, surface charge, and protein state (native or denatured states), respectively. The amino acid residues lining the binding cavities and their related conformational changes are effectively studied by observing the Xe-induced chemical shifts of nearby nuclei, e.g., ^1^H, ^15^N and ^13^C. For example, a mutant protein with engineered cavity indicated increased chemical shift values (ca. 0.4–0.8 ppm) compared to small chemical shifts in the case of the wild type counterpart (0.05 ppm) [27]. In certain cases, higher chemical shift values (>1 ppm) have been reported for nearby protons available in close proximity to the Xe binding pockets/cavities in different proteins. Due to the non-specific binding of Xe to all available cavities in proteins, a fast Xe exchange and a shorter T_1_ are typically reported. In a special case, Xe binding to a cavity residing near a paramagnetic center of a protein was shown to foster a change in the magnetic behavior of the observed Xe-protein inclusion complexes [28,29]. Xe measurements in this relative fast exchange regime are unable to further specify the number of available cavities/pockets.

The changes occurring in the ^129^Xe chemical shift upon non-specific binding to the protein cavities was expressed in terms of a concentration-normalized chemical shift (α, in ppm/mM). The observed α is dependent upon the protein state (native or denatured state), structure, charge, and conformation of the protein, respectively [30]. With the onset of ^129^Xe HyperCEST NMR, the proteins can be characterized by Xe interactions occurring at the cavity and or at the surface via the different exchange pools coupled to free bulk Xe in solution. A “three-site” model explains the non-specific Xe binding in proteins based on three different environments, namely Xe@aq, Xe@cavity and Xe@surface, respectively. Direct measurement of non-specific Xe interaction with protein surfaces is not straightforward as it explicitly depends on the protein’s conformation and characteristics of surface amino acid residues, e.g., charge, polarity, hydrophobicity etc. [31]. 

### 2.2. High-Affinity, but Reversible Binding in Synthetic Structures 

Similar to biomolecular NMR of proteins, Xe NMR was utilized to examine the properties of different porous systems such as zeolites [32,33], aluminophosphates [34,35], polymers [36,37,38], self-assembling dimer [39], organic zeolite isomorphs [40], and metal organic framework (MOF) [41], respectively. Details pertaining to pore size, pore volume and surface area can be obtained through different techniques, e.g., powder X-ray, TEM, adsorption isotherms etc. However, only ^129^Xe NMR is capable of probing the connectivity and uniformity of the pores that remain inaccessible to other conventional techniques. For example, activation-induced flexibility in a porous MOF, e.g., Ni_2_(2,6-ndc)_2_(dabco) was examined using ^129^Xe NMR [42]. The line width and chemical shift of the ^129^Xe NMR signals are very sensitive parameters involved in determining the MOF’s structural transition from a narrow pore system with low porosity to a wide-pore state. The peak with a larger chemical shift indicates that Xe occupies a smaller pore compared to peak displaying smaller shift. The large chemical shift range of ^129^Xe is strongly dependent on both local environmental and chemical factors, e.g., composition of the matrix, nature/concentration of adsorbed molecules, and shape/size of void spaces, respectively [43]. A sharp peak reveals that Xe is located in a more homogeneous environment that facilitates increase in dynamic processes [44]. Additionally, chemical shift anisotropy, longitudinal relaxation time (*T*_1_), temperature also serve as important parameters that encode the information about Xe interaction at the pores and surface of MOFs [45].

However, most of these interactions are rather loose since the binding cavities are not really tailored to Xe as a guest (van der Waals radius: 2.16 Å; *V* = 42.2 Å^3^ [46]). This has two consequences: (1) bound Xe is in many cases not revealed by a well resolved additional signal but rather through a shift of the bulk pool signal. (2) Observable low affinity constants cause the bulk pool of free Xe to be by far the dominant one and require high host concentrations to identify a potential second signal. Experimental evidence demonstrated that the ratio of guest to cavity volume is an important parameter: according to Rebek’s rule, 55% of the cavity volume should be occupied by the guest for optimal molecular recognition [47]. Cryptophanes are an example for early studies of hosts with efficient encapsulation for small guests [48] and they were therefore also considered for binding of Xe. A strong NMR signal of caged Xe was then pursued by optimizing the cavity to increase the affinity constant *K*_a_ (see also Section 5.1). The exchange rate, however, was originally of minor importance, and in many cases an improved affinity comes with a decreased exchange rate. Section 4.2 will discuss why this interplay nowadays appears in a different light for ultra-sensitive detection. Excessive exchange rates are not desired either because they suppress a sufficiently resolved signal of trapped Xe. 

Well defined cavities are thus one way to achieve functionalized Xe for efficient NMR detection. This class of hosts typically binds one Xe atom at a time. The large chemical shift range of ^129^Xe also allows an alternative approach, namely partitioning into a different micro-environment. This concept allows the definition of volumes that bind many Xe atoms. Perfluorooctyl bromide (PFOB) nanodroplets and hollow protein structures as discussed in Section 5.10 are examples for this approach. Good “affinity” is then achieved through a high solubility coefficient (for liquid phase PFOB) or efficient partitioning (for gas phase hollow compartments in protein assemblies). While this is different from high-affinity cavities like in cryptophanes, it still defines a detectable second spin pool of functionalized Xe.

## 3. Production and In Situ Delivery of hp Xe

The Zeeman splitting of nuclear spin states is a minor energy correction term in technically achievable external fields. While this is an advantage with respect to the harmless RF irradiation that is used to acquire NMR/MRI data, it has major consequences for the achievable spin polarization at room temperature. Under thermal equilibrium conditions at ~300 K, the high temperature approximation applies and the system provides a rather minute “thermal polarization” in the presence of an external magnetic field. Thus, the NMR signal of dilute molecules is only detectable after extensive signal averaging and rather low SNR values. A higher concentration of the molecule of interest is typically unavoidable in order to achieve the desired NMR signal under thermal polarization conditions. To boost NMR sensitivity and to rapidly acquire signals with high SNR, hyperpolarization (hp) is actively being utilized for different reporter molecules. In hp conditions, the nuclear magnetization is artificially enhanced prior to the NMR experiment. Generally, the process provides 10^3^–10^5^ fold higher signal enhancement compared to the thermal polarization [5,13,49,50]. Electromagnetic radiation on the nuclear Larmor frequency cannot be used to directly overpopulate a spin state. Instead, all hyperpolarization techniques have in common that they rely on a precursor system that is polarized in a first step before the polarization is transferred onto the detected nuclei [5]. Two of the common hp approaches, DNP (dynamic nuclear polarization) and PHIP/SABRE (para-hydrogen induced polarization/signal amplification by reversible exchange), polarize covalently bound nuclei and thus the delivered tracer is part of the entire polarization procedure. Conversely, hyperpolarized ^129^Xe gas can be generated by applying cryogenic or brute force techniques that utilize a very high magnetic field and millikelvin temperatures [51]. The major advantage of brute-force polarisation is that no exogenous agent needs to be added to the sample. A limitation is that the signal enhancement scales linearly with the pre-polarisation field. Thus, it is an attractive method for applications where the detection field is very low and doping the sample is not feasible [52]. Additionally, this technique results in an extremely long T_1_ of nuclei in solids at very low temperature which can be avoided by addition of liquid He in order to reduce T_1_ to a few hours. For example, Hirsch and co-workers demonstrated that if pre-polarisation is carried out in a field of 14 T and at ~2.3 K, >0.1% ^13^C polarisation can be observed for metabolites, e.g., 1-^13^C-acetic acid and 1-^13^C-pyruvic acid etc., upon rapidly heating and subsequently detecting them at 1 T and 303 K [53]. Although observed polarisation levels are significant, the cost associated with this high-field/low-temperature instrumentation is of concern for routine NMR. 

For Xe biosensors, however, only the noble gas undergoes the hp procedure and it is also delivered separately from the molecular host structure. This means that the preparation of the detected spin species happens entirely in the gas phase, followed by dispersion of Xe into the solution or sample of interest.

### 3.1. Spin Exchange Optical Pumping (SEOP)

**Figure 1:**
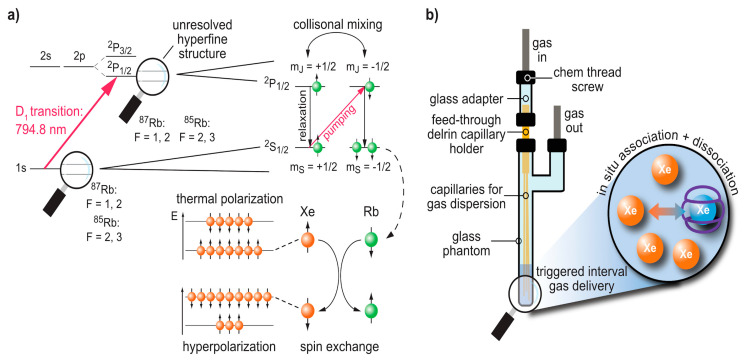
Production of hyperpolarized ^129^Xe and reversible in situ association with a host. (**a**) The SEOP process constantly maintains a high polarization of the Rb electron spin system through selective pumping of the D_1_ transition. This yields an overpopulation of one of the two spin states in the ^2^*S*_1/2_ ground state. Such polarized Rb serves as the source to polarize Xe by spin exchange, e.g., through a binary collision. The ^129^Xe spin system is eventually converted from thermal polarization to a strong overpopulation far outside the thermal equilibrium. (**b**) Xe delivery into a sample solution and reversible binding to a host.

The common technique for obtaining hp ^129^Xe is spin exchange optical pumping (SEOP) [5,13,54]. The above-mentioned precursor that undergoes initial spin hyperpolarization is an alkali metal vapour. This is a system with a single valence electron which can be easily manipulated through laser irradiation. Rubidium (Rb), e.g., melts at 39.3 °C and yields sufficient spin density in a gaseous atmosphere when operated at temperatures up to 200 °C. This alkali metal has its D_1_ transition at ~795 nm, i.e., it can be easily pumped by a strong infrared (IR) laser (see Figure 1a). Modern laser diode arrays achieve ~10^2^ W cw output to obtain a beam that passes optical elements to yield circularly polarized light. Such powers are sufficient to illuminate pumping cells volumes of several 100 mL. The pumping cell is not only exposed to the IR laser beam but also to a static magnetic field in the mT range that is aligned with the optical axis. The magnetic field causes a Zeeman splitting of the otherwise degenerate Rb electron spin states and its combination with circularly polarized photons causes a selective spin flip transition from the ground to the first excited (^2^*P*_1/2_) Rb electron state. The thermal energy inside the system causes immediate equilibration between the spin sub-levels of the ^2^*P*_1/2_ state due to collisional mixing. To avoid unwanted emission of photons with opposite circular polarization, a quench gas is added (typically ~10% N_2_) that enables radiation-free relaxation into the ground state. While this relaxation represents an influx of population for the two spin sub-levels of the ground state, the continuous selective depletion due to the optical pumping with IR photons rapidly yields a net over-population of one of the spin states. 

In a second step, ^129^Xe atoms entering the pumping cell pick up the polarization from the Rb electrons through polarization transfer in a flip-flop process. The laser systems are designed to provide sufficient photon flux for immediate re-polarization of the Rb electrons. To ensure efficient laser absorption for systems with laser emission bandwidths exceeding the Rb absorption profile, He is often added as a buffer gas (ca. 80% of the total gas mix). This causes pressure broadening for matching the diode emission profile with the D_1_ absorption profile. The cross-relaxation phenomena (spin exchange) are characterized by spectral densities and consequently dependent on the molecular interactions occurring between the Xe and the alkali metal. Such interactions can either be simple two-body hard-core collisions or three-body collisions leading to the intermittent generation of alkali metal/Xe van der Waals molecules [55]. The three body collisions increase the dipolar correlation time, thereby leading to more efficient cross-relaxation rates. Increasing the pressure results in a cross-over that shifts from a dominant three-body process to the two-body collision model. This process predominates at “high pressure” (>1 bar). Conversely, three-body collision leads to the creation of transient Rb–Xe van der Waals molecules whose lifetime depends on the overall pressure. Further, three-body collision is prevalent at low pressure leading to much longer dipolar correlation times and thus much more efficient spin-exchange mechanism [49,54]. The overall SEOP efficiency strongly depends on the pressure and temperature conditions inside the pumping cell. Recent studies have demonstrated near-unity spin polarization [56]. 

Early applications with direct delivery of Xe into the liquid sample often had only 1% Xe in the gas mix [57]. Increasing this fraction is often desired for achieving a higher Xe concentration in solution. However, more Xe entering the pumping cell in continuous flow also depolarizes the Rb faster and requires a sufficiently strong laser system to maintain the Rb polarization bath without risking a breakdown of the process. Regarding the role of the N_2_ quench gas at high laser power, it can become a challenge to manage the side effects of the heat that is deposited into the gas mix by strong lasers: if the Rb is vaporized directly from a droplet inside the pumping cell, the conversion of photon energy into heat causes further vaporization of Rb. This process is called “rubidium runaway”, and it is a deleterious, self-amplifying effect that causes rather inhomogeneous illumination of the pumping cell. Eventually, this yields poor Xe hyperpolarization. It requires appropriate temperature management inside the setup [58] to keep the runaway effect under control.

As mentioned above, an adjustable Xe fraction in the gas mix can be advantageous. Thus, on-site combination of N_2_ with Xe (and He) as implemented through mass flow controllers at the polarizer inlet in combination with a pressure controller behind the pressurized sample is the preferred way to operate such a SEOP setup for such applications [59]. The sample is then directly fed from the polarizer outlet. With a natural abundance of 26% for ^129^Xe, non-enriched Xe is often sufficient for many NMR applications, particularly in vitro studies. However, extensive signal averaging can be a challenge for in vivo applications with low spin densities. These studies can benefit from isotopically enriched ^129^Xe (~80%) in the interest of total acquisition time. 

### 3.2. Delivery and Handling of Xe Compared to Other hp Tracers

**Figure 2:**
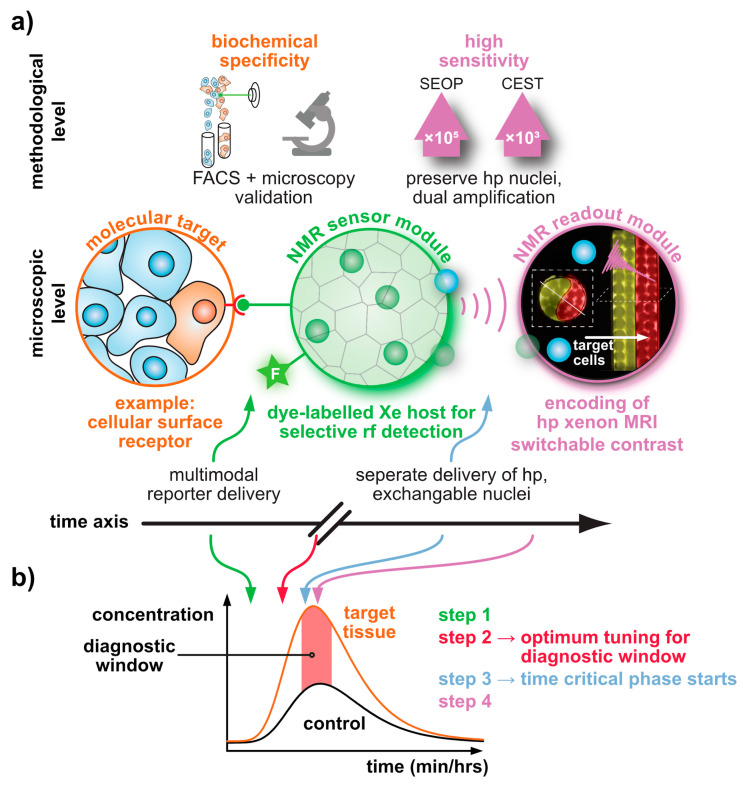
Separating the sensing and the detection module for functionalized Xe. (**a**) The sensor unit is pre-delivered and can carry fluorescence markers for cross-validation with cell sorting (FACS) and microscopy. Spontaneous, reversible binding of the subsequently delivered hp ^129^Xe combines high sensitivity from SEOP and chemical exchange saturation transfer (CEST) with high specificity. The lifetime of the hp nuclei is somewhat decoupled from the overall time it needs for sensor accumulation in the target tissue. (**b**) This decoupling allows to achieve an optimized diagnostic time window where the sensor concentration in target and control tissue differ significantly.

In comparison to DNP and PHIP/SABRE applications that polarize the reporter molecule, the Xe biosensor concept follows a modular approach: The detected nuclei undergo SEOP separately from the molecular host that confers a dedicated chemical shift to the Xe nuclei. Thus, the gas simply needs to be bubbled into the sample solution to participate in spontaneous, reversible binding into the host’s cavity (see Figure 1b). For sensing a specific analyte or cellular target, the hosts can be coupled to a targeting moiety. This assembled sensing module is then delivered without the hp nuclei and Xe delivery occurs only after specific binding/interaction of the latter one has taken place in the target volume and clearance has occurred in the control areas. This is illustrated in Figure 2a and is possible due to the spontaneous in situ association of the hp nuclei with the host structures. The concept of the “relaxation clock” of hp ^129^Xe practically being stopped until the gas encounters its first interaction partners in solution or in tissue is also illustrated in Figure 2a with the broken time axis. Hyperpolarized ^129^Xe encounters the challenge of T_1_ reduction in the presence of paramagnetic species e.g., oxygen and blood, competing vascular delivery timescale etc. [60] Introduction of optical reporters onto the hosts or host derivatives enables cross-validation of the molecular targeting on the cellular level through microscopy and cell sorting. Optimized incubation conditions can be revealed and Xe delivery and detection are occurring at a later step (see Figure 2b). 

This concept thus offers sensing options that are complementary to the other hp techniques. While the latter ones focus on (fast) metabolic processes, Xe biosensors address molecular markers on a time scale beyond metabolism. In fact, Xe as a noble gas is not part of any metabolically active tracer. Its polarization life time (given by the time constant *T*_1_ for longitudinal relaxation: *T*_1_ ~7–8 s in arterial human blood [61]; 15–16 s in mouse brain [62]) is shorter for biomedical applications than those of currently used ^13^C and ^15^N tracers in hp NMR/MRI (*T*_1_ ~25 s at 3 T in vivo [63]). Some compounds have been reported with very slow relaxation, e.g., ^15^N-enriched choline (~4 min [64]) and ^15^N-enriched nitro compounds (~100 s [65,66]). The relaxation of hp Xe can be somehow preserved by dissolving it in an appropriate medium, e.g., Intralipid [67] or perfluorooctyl bromide (PFOB) emulsions [68] prior to its in vivo administration. Otherwise, hp Xe also suffers from faster relaxation similar to the ^13^C tracers. Of course, freezing the as-produced hp Xe might prolong the relaxation time for transportation between the SEOP production and the imaging sites. While this is challenging for Xe MRI beyond the lung, it is still feasible, as demonstrated by recent encouraging advancements in human brain and kidney MRI with dissolved Xe [69,70].

The *T*_1_ relaxation properties of hp Xe are very favorable for ^129^Xe spins remaining in the pure gas phase. Relaxation times of 4.6 h at room temperature and 5.75 h in a dedicated storage cell at elevated temperatures have been measured [71]. This enables opportunities for off-site (batch) production and storage of hp Xe and its later use in an NMR setup. This is a much simplified handling compared to clinically established ^13^C DNP studies that require a streamlined infrastructure for production, quality assurance, and delivery to the application site within a rather narrow time window of ca. 60 s [72]. 

Early applications of hp Xe produced the gas in batch mode where it was collected in a cold finger for cryo-separation from He and N_2_ upon leaving the pumping cell. The solidified Xe was sublimated and the pure gas was then transferred into a sample tube and mixed with the sample solution by shaking [73]. This yields a high ^129^Xe NMR starting signal with but leaves only a limited data acquisition time window if no re-delivery occurs: the magnetization is continuously decaying due to *T*_1_ relaxation and due to the applied rf readout pulses. Many studies therefore switched to bubbling the entire gas mix into solution [57,74], a method that allows extensive signal averaging with repetitive delivery albeit at a lower SNR per individual readout. However, with improving polarizer performance, meaningful data can often be acquired within a few acquisitions. Depending on the analyte under investigation, different solvents might be required. Various carriers with different Xe solubility have been investigated [75], including saline solution, lipid emulsions, and plasma expanders. As Xe hosts often come with a limited water solubility, DMSO is also an alternative [76]. Future applications might also benefit from Xe carriers like liposomes that can release their gas load upon ultrasound treatment [77] and thus locally provide a high Xe concentration after improved transit conditions.

## 4. Signal Transfer Detection Concepts for Xe

Although the preparation of Xe as a hp spin system provides a significant signal enhancement, the nuclei are not necessarily detected directly in a conventional NMR spectrum. Especially for studies with Xe dissolved in solutions containing other molecules of interest, the interactions of Xe with other nuclei might provide routes of signal transfer detection that also come with enhancement. This can include polarization transfer from protons with a larger Zeeman splitting or, vice versa, from hp Xe onto nearby nuclei. Another option is the signal transfer between multiple Xe pools that differ by chemical shift and exist as an abundant pool for detection that is in chemical exchange with dilute pools representing interaction partners of Xe. 

### 4.1. Spin Polarization Induced Nuclear Overhauser Effect (SPINOE) 

Investigating the properties of the Xe chemical environment is also possible via selective polarization transfer from ^1^H to ^129^Xe. This approach promoted some findings linked to xenon binding sites [78] and preferential Xe solvation [79]. These experiments provide direct information about the xenon environment and can be helpful for interpreting observed Xe chemical shifts albeit this effect relies on weak intermolecular cross-relaxation between ^129^Xe and nuclear spins in the immediate environment. Additionally, a selective irradiation within complex ^1^H spectra to enable SPINOE might be hard to achieve and performing a 2D ^1^H-^129^Xe heteronulcear NOESY can also lead to long acquisition times. Alternatively, Xe polarization can also be transferred to different nuclei of molecules in solution [80,81,82] and to surfaces [83,84,85,86] via cross-relaxation through a process called spin polarization-induced nuclear Overhauser effect (SPINOE). For example, transient binding of Xe to α-cyclodextrin indicated distance-related cross relaxation rates leading to generation of signal enhancements of protons present in the proximity of the Xe binding site [81]. The SPINOE polarization transfer experiments can be utilized for studying the structure and dynamics involved in the molecules with which Xe interacts and also accordingly the former’s hydrophobic potentials. 

Cryptophane-A (CryA) was employed as a model system for investigating the SPINOE characteristics experienced between Xe and CryA protons lining the internal cavity. The Xe/CryA complexation occurs without a high degree of constrictive binding compared to other Xe hosts, thereby giving rise to Xe resident times in the order of milliseconds [87]. The SPINOE experiments of CryA were performed in (CDCl_2_)_2_ solvent in which two peaks were noticed for free and bound Xe at ~160 and ~60 ppm, respectively. The relaxation of ^129^Xe is a slow process compared to its exchange rate. Additionally, the relaxation time of ^129^Xe utilized in this model system is slow compared to ^1^H relaxation which might be beneficially used while performing ^129^Xe to ^1^H polarization transfer. Considering the impact of cross relaxation between ^1^H-^1^H (zero at RT) occurring for CryA, the ^129^Xe-^1^H polarization transfer was expected to attain 70% of its maximum value. The SPINOE spectra for Xe inside CryA were obtained at different conditions such as (1) positively polarized ^129^Xe; (2) Xe at equilibrium; and (3) with negatively polarized ^129^Xe, respectively, and compared to ^1^H equilibrium data of CryA (ca. 50 mM). The equilibrium spectrum was dominated by signals from methoxy groups while the SPINOE indicated signals assigned to aromatic protons. A signal enhancement between 3 and 13% was achievable for protons of CryA due to SPINOE action on it. This proton enhancement due to Xe binding was proven by observing 2 fold higher values from SPINOE compared to H-Xe cross relaxation rates anticipated from diffusive coupling. The average distance between the aromatic proton and Xe was found to be within 4.1–5.2 Å; Xe positioned at the center of the cavity would be in a range of 3.2–3.8 Å to the nearest proton. Based on SPINOE results, it was shown that the most probable conformation of the spacer bridges in CryA was a *gauche* one while complexing ^129^Xe. The chemical shifts pertaining to proton pairs present in the linker OCH_2_CH_2_O indicated that they exist in different conformations to each other, namely *gauche* and *anti* with respect to vicinal oxygen atom. Thus, SPINOE experiments are helpful for obtaining detailed microscopic information by directly probing the molecular structure and dynamics in solution [88].

The cavities available in different proteins are often studied using either X-ray crystallography under Xe overpressure or through hydration studies using liquid state ^1^H-NMR, respectively. X-ray diffraction studies are limited since they do not provide information on dynamic properties involved during interaction between cavities and probe molecules and also require investigation under high pressure [89,90]. This issue can be easily circumvented using liquid state ^1^H-NMR involving solvent-protein proton cross relaxation albeit it should not be masked by the inherent water and hydroxyl proton exchanges [91]. To avoid such conditions, small probe molecules are utilized in ^1^H-NMR to probe the cavities available in proteins via the cross relaxation occurring between the protons of small molecules and of the proteins. This might require application of small molecules at higher pressure, e.g., methane at 200 bar in order to obtain better “loading” conditions for improving the sensitivity of this ^1^H-NMR-based NOE approach. However, usage of higher pressure might induce structural modifications in proteins [89] or unfold protein structure [92], thus hindering NOE-based applications. Hp ^129^Xe with high sensitivity was utilized as an atomic probe to map the cross relaxation between ^129^Xe and protons of molecules of interest, e.g., benzene, via the SPINOE approach. Since ^129^Xe does not induce structural changes on medium sized host molecules, it might be a useful probe for studying cavities in proteins using SPINOE experiments [93]. Instead of indirectly characterizing the interaction between the protein and Xe, a direct characterization was achieved using heteronuclear cross relaxation occurring between hp Xe and a protein, e.g., the non-specific lipid transfer protein (ns-LTP). Ns-LTP from wheat is a 90-amino acid protein belonging to the ubiquitous ns-LTP family in the plant kingdom [94] and it is responsible for transporting amphiphilic molecules of different sizes between the membranes in vitro. Its structure is stabilized by four disulfide bridges and comprises four α-helices packed against a C-arm [95,96,97]. In the case of wheat ns-LTP, a hydrophobic cavity size of about 400 Å^3^ [98] was observed and it was shown to increase up to 750 Å^3^ [99] in the presence of aliphatic part of lipid at the cavity leading to shape like a tunnel [100,101,102]. Generally, interactions occurring between small molecule probes and hydrophobic cavities are evaluated either using higher pressure of interaction partners such as methane or ethane or by utilizing saturated solutions of benzene, cyclopropane, cyclohexane (albeit without a possibility to define their exact location inside the investigated cavity) [103].

The number of Xe binding sites in ns-LTP from wheat was determined through solvation simulation and was supported experimentally by SPINOE polarization transfer between Xe and the protein. Additionally, the chemical shift variation of amino acid protons lining the hydrophobic cavity also confirmed the number of available Xe sites in ns-LTP. In this context, eight of the amino acids displayed chemical shift variation of >0.02 ppm for Xe pressures below 5 bar, while for the other 8 amino acids a slight variation of >0.01 ppm was observed. The three sites found by solvation simulation were confirmed by SPINOE experiments and these measurements indicated that only one Xe probes the three sites available at the protein. However, the presence of two additional binding sites were only revealed through SPINOE results instead of by solvent simulation. Further, only one peak was observed for Xe, i.e., Xe in solution (189 ppm) upon interaction with protein and due to fast Xe exchange no bound Xe peak was observed using direct Xe NMR. Through SPINOE approaches, the proton signals from methyl groups of amino acids present at the cavity were enhanced between 1% and 3% upon polarization transfer from Xe (e.g., 2% enhancement with 200 ms mixing time). However, the amino acids available at the surface of the protein reveal a chemical shift variation in the presence of Xe which remains undetected by SPINOE approach. The effect might be hampered due to the small local structural modifications and/or by too short Xe resident times on the protein surface. The partial site occupancies demonstrated through chemical shift variation under variable Xe pressure indicated the existence of complex dynamics that might affect the protein as a whole on the large time domain [104]. Unless other small molecules are utilized as probes for protein study, Xe binds specifically and is located in well-defined sites without the necessity for structural distortions of the protein under investigation [105]. 

To this end, a single study reported somehow the possible clinical adaptation of SPINOE technique [106]. Briefly, this study demonstrated the SPINOEs occurring between [1,4-^13^C_2_]fumarate hyperpolarized via DNP and solvent water protons. 20 mM fumarate hyperpolarized to 36% via DNP resulted in ~2% SPINOEs enhancement at 9.4 T. Additionally, the SPINOE increases at lower field strengths for fumarate (~14% at 3.4 T), suggesting that it might be applicable to the field strengths utilized in the clinic (1.5–3 T). Thus, SPINOE might offer the possibility of following the progress of a bolus of hyperpolarized ^13^C-labeled material in the bloodstream by acquiring the signal from solvent protons rather than from ^13^C metabolites, thereby keeping the hyperpolarization of the latter untouched. Overall, this study demonstrates the usage of the SPINOE technique as a potential approach for studying different tissues beyond the perception of being considered only as an NMR-based analytical tool.

Xe bound to hydrophobic cavities results in xenon–proton cross-relaxation rates that are several orders of magnitude lower than the intramolecular proton–proton cross-relaxation rates. As a consequence, the xenon polarization is transferred to the nearby protons of the protein through dipole–dipole interaction. The available hyperpolarization is thereby ‘diluted’ throughout the network of more remote protons. To quench this spin-diffusion, a proton off-resonance rf field is utilized during the mixing period. This approach, which is also a derivative of the SPINOE experiment, has been introduced with the acronym SPIROE [107]. For a SPIROE experiment, the difference of precession frequency between the proton magnetization spin-locked along the effective field and the xenon magnetization scales down the transfer efficiency. Other possible limitations of SPIROE are i) instability due to sample or coil heating (e.g., when a long radiofrequency (rf) irradiation is used), or ii) variations of the internal dynamics leading to nonvanishing proton-proton cross-relaxation rates. The benefit of using the SPIROE approach over the SPINOE was established by investigating the interaction between laser-polarized xenon and a cryptophane derivative in 1,1,2,2-tetrachloroethane. In this context, *C*_2_-symmetrical cryptophane-233 is particularly well-designed for SPIROE experiments as its dissymmetry allows discrimination between the different protons inside the cavity. Generally, all resonances present in the ^1^H spectrum appear albeit with different intensities in a SPINOE spectrum. Contrarily, the SPIROE spectrum detects only the aromatic protons, the protons of the aliphatic chain in *C*_2_ and, with lower intensity, the equatorial protons of the methylene bridge groups. Thus, SPIROE experiments serves as a new tool for quantifying interactions between a hp gas and its neighboring atoms through the estimation of the average distances between xenon and protons, as well as the intermolecular dynamics [108].

### 4.2. HyperCEST and MT Detection 

**Figure 3:**
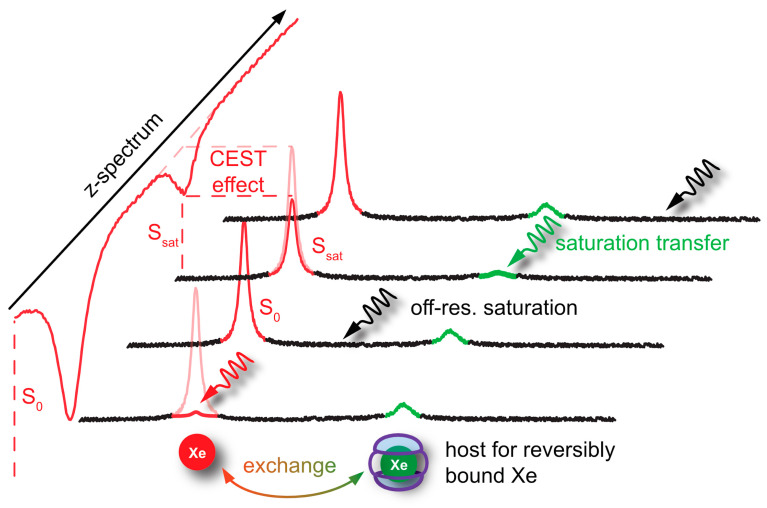
Principle of CEST detection. The signal of unbound Xe can be detected at high signal-to-noise-ratio while the nuclei are in constant exchange in and out of the binding site of the host. Direct saturation yields a complete signal loss while saturating onto the reversibly bound Xe yields a partial signal loss for the respective frequency offset. Sweeping the saturation frequency over a larger frequency range in a pseudo-2D experiment yields a z-spectrum with the complete shape of the CEST response.

In cases where Xe can undergo transient binding with a host structure in a relative slow exchange regime (relative to the NMR time scale), two ^129^Xe NMR resonances can be observed: one for the bulk pool (denoted as pool *A*) and one for the bound pool (pool *B*). Such two signals are often well separated due to the large chemical shift range and can thus be addressed individually by selective radiofrequency (rf) pulses. If pool *A* is the dominant pool, it can be used to enhance the sensitivity for detecting pool *B*. This was first applied in a study where the *A* pool served as a magnetization reservoir while the signal from *B* was repeatedly detected with selective rf pulses that cause a stepwise loss of hp magnetization [109]. However, there is a more efficient way to benefit from the spin exchange between the two pools: chemical exchange saturation transfer (CEST) is a method where the magnetization in pool *B* is saturated, i.e., nullified, by means of a (cw) rf pulse that is applied over a time window that is long compared to the average residence time of the transiently bound spins. Chemical exchange during this saturation period thus causes a signal loss in *A* as hundreds to thousands of spins are affected per exchange site and accumulate in the detection pool. This relative signal loss (*S*_ref_ − *S*_sat_)/*S*_ref_ from the saturated scan (*S*_sat_) vs. the reference scan (*S*_ref_, without saturation) is detected immediately after the saturation period (see Figure 3). Importantly, the CEST method allows to preserve the spectral dimension: applying the saturation pulse at incremental frequency offsets prior to detection of the bulk pool enables a stepwise sweeping along the chemical shift dimension. The overall shape of this so-called *z*-spectrum (i.e., plotting the remaining *z*-magnetization after each saturation offset) carries information about the connected pool sizes and exchange dynamics between them. The combination of CEST and hp nuclei, coined as HyperCEST [17], offers several advantages for an ultra-sensitive detection as it joins two amplification strategies under rather favourable conditions. Its strength becomes obvious when placing it in perspective with CEST applications based on thermally polarized nuclei.

The concept of exchanging nuclei was initially used by Forsén and Hofman to study chemical exchange reactions of comparable pool sizes [110,111]. It later found applications in ^1^H-MRI to generate image contrast in the abundant water pool through a dilute saturated pool where the term CEST was introduced [112]. Many versions of CEST with endogenous substances bearing labile -OH, -NH, and NH_2_ protons have now been implemented [113,114]. However, the range of chemical shifts in ^1^H-NMR is limited and makes it difficult to selectively saturate pool *B* without affecting pool *A* (this is described as the so-called spillover effect that needs further corrections for quantifying the CEST response [115,116]). An improved spectral resolution is provided by paraCEST agents that work with exchanging water molecules from paramagnetic chelation complexes [117], many of them being responsive to their biophysical or biochemical environment [118]. The exchange rates of the coordinated water is, however, often high and requires high saturation pulse powers and relative high concentrations [119]. In any case, CEST with thermally polarized nuclei is always limited by the intrinsic *T*_1_ relaxation that counteracts the rf-driven saturation effect. The bulk pool thus typically only reaches a steady state of incomplete saturation and benefits from high-field setups where *T*_1_ is increased and makes the saturation transfer more efficient [120]. 

**Figure 4:**
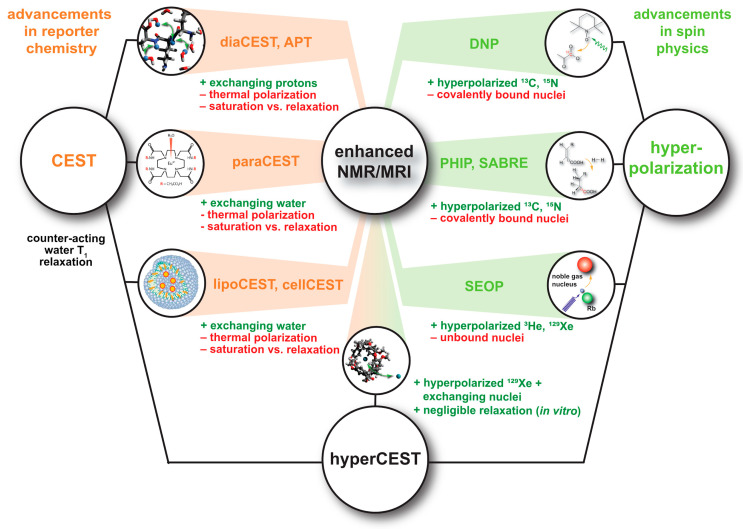
HyperCEST combines advantages from advancements in reporter chemistry and spin physics, i.e., the enhanced magnetization from a pre-polarized spin system plus the efficient use of exchanging nuclei to transfer information from a dilute to a bulk pool. Pros and cons for each technique are shown in green and red, respectively. Thermally polarized CEST systems are typically limited by the counter-acting *T*_1_ relaxation that limits the achievable saturation effect.

HyperCEST combines several advantages that apply otherwise only for either diaCEST or for paraCEST agents [121]. Figure 4 illustrates that it benefits both from favourable CEST parameters and the signal enhancement given through the hyperpolarization process. Reversibly bound Xe is thus an ideal system for CEST sensing because of the following aspects: Firstly, the large chemical shift range typically provides conditions where the bulk pool signal is well separated from the bound Xe signal. Most studies have been performed at field strengths of *B*_0_ = 7 to 9.4 T where the Larmor frequencies of both pools are separated by several kHz. This leaves much room for performing studies also at lower field strengths. Secondly, values for the exchange rate, *k_BA_*, of currently known hosts for HyperCEST are in the range *k_BA_* ~10^1^–10^3^ Hz and therefore do not require excessively large *B*_1_ pulse powers for achieving a decent saturation response. In fact, excessive saturation only causes broadening of the CEST response in the *z*-spectrum once all Xe entering the host is practically instantaneously saturated [122]. Thirdly, hp Xe nuclei in sample solutions undergo rather slow *T*_1_ relaxation towards the relatively small thermal magnetization, i.e., the relaxation is not counteracting the buildup of the CEST effect. The pool of unbound hp ^129^Xe is ideal to “store” the CEST information. Given sufficient host concentrations (e.g., cryptophane-A in the µM range) it can cause complete saturation of the detection pool. It is eventually only limited by “back exchange”, i.e., Xe atoms repeatedly entering the host after being already saturated. 

Nevertheless, relaxation of the hp nuclei is not irrelevant for HyperCEST. Build-up of the signal loss in pool *A* needs to be faster than its intrinsic *T*_1_ relaxation [122]. This is at least for in vitro systems easy to achieve. A critical aspect is the combination of the host loading efficiency, *β* (for molecular hosts based on chemical affinity, this is characterized by the binding constant *K*_A_), and the exchange rate *k_BA_* from pool *B* into pool *A*. The net effect is then characterized by the gas turnover rate *βk_BA_* as introduced by Kunth et al. [123]. 

In some cases, Xe hosts with a defined cavity have shown an unexpectedly high exchange rate such that the observation of a dedicated signal from bound Xe becomes difficult or even impossible [124,125]. The presence of the host pool is nevertheless detectable in the z-spectrum and manifests as a significant broadening of the saturation response from the bulk pool. Slight off-resonance saturation causes a “magnetization transfer” (MT) response that is also known in ^1^H-MRI to detect a water pool bound to macromolecular structures which undergoes exchange with the detected, freely tumbling water [126]. The MT effect can be of a considerable amplitude when compared to samples that lack the fast exchanging pool. However, it does not provide the spectral specificity that comes with a dedicated HyperCEST response. 

The host designs discussed in the following sections evolved around these aspects of the gas turnover rate, the spectral resolution (for combining high specificity of the signal detection with high sensitivity from HyperCEST), and the options to couple targeting groups onto the hosts for specific interactions with different analytes. The right combination of these parameters enables generation of ultra-sensitive Xe biosensors.

## 5. Aspects of ^129^Xe Biosensor Design

**Figure 5:**
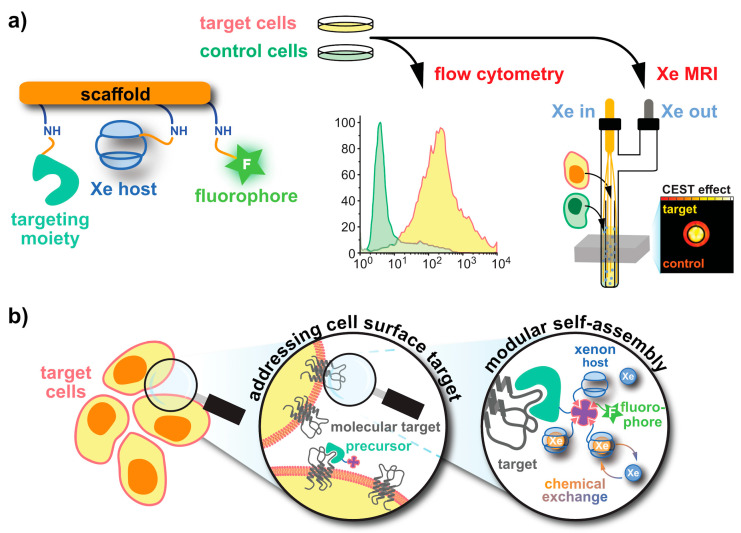
Strategies for obtaining functionalized Xe sensors. (**a**) A Xe host and targeting unit can be coupled to a (peptidic) scaffold via peptide synthesis at activatable tethers (orange). An optional dye label enables combined fluorescence and MRI specificity tests. (**b**) In a modular approach, a functionalized precursor (e.g., an avidin-activated binding motif) can be delivered to the target. Subsequent delivery of biotinylized Xe hosts and dyes yields the complete sensor (see Section 5.2.1).

The key elements of Xe biosensors comprise the Xe host, a tether or spacer, and the ligand for a certain molecular target [16]. The development focusing on certain building blocks fostered strategies in which these units are typically combined on a scaffold or backbone and often include a fluorophore for cytometry to check for binding specificity (see Figure 5a). Peptide synthesis concepts are often used for the coupling but modular approaches as illustrated in Figure 5b are also an option. These will be discussed later on. Many of the early known Xe interaction partners show an unspecific binding and/or rapid exchange with no dedicated NMR resonance of bound Xe that can be used for CEST detection. The observation of reversible formation of a Xe@cryptophane inclusion complex in solution [87] fostered the idea to further develop this concept into a sensing platform. Cryptophanes with their tailored cavity sizes have thus been used in many sensor designs as starting material for coupling with binding motifs for different biological targets [15,127,128,129,130,131]. Further development of the HyperCEST approach, however, showed that there are other hosts with better Xe exchange dynamics [123,124,132,133].

### 5.1. Cryptophanes as Xe Hosts 

**Figure 6:**
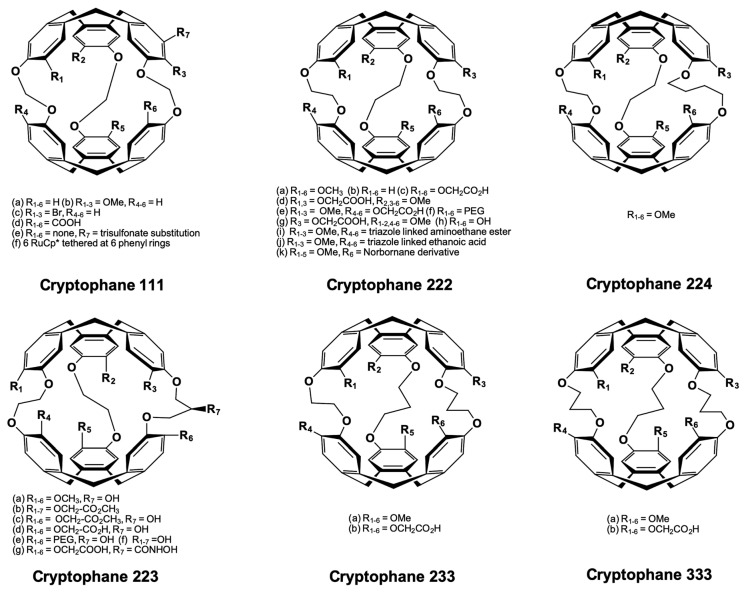
Different cryptophanes and their derivatives that were evaluated as potential ^129^Xe hosts. All above cryptophanes are shown to bind hp ^129^Xe to a varying degree which was reflected through the observed difference in the binding constants.

Cryptophanes were first synthesized as molecular containers for studying their binding capabilities to different guest molecules [48,134,135,136]. These hydrophobic containers are formed by connecting together either one of the caps such as cyclotribenzylene (CTB) (often called cyclotriveratrylene (CTV)), cyclotriguaiacylene (CTG), or cyclotriphenolene via linkers of different lengths in solution. Generally, alkyl dioxy linkers are introduced in between the two similar caps in order to generate the cryptophane containers. Cryptophanes have been classified based on the *nml* “core” nomenclature, where *nml* represents the number of carbons available at the linker. Different cryptophanes have been synthesized, including cryptophane-000, 111, 222, 333, 444, 555, 666, and also intermediate ones such as 223, 233, 224, 122, 112, respectively [134,135,136,137]. An overview of this host family members is given in Figure 6. Without considering the asymmetry of the linkers, cryptophanes are classified based on the caps as *syn* and *anti* isomeric structures. Connecting the two CTB caps with opposite helicity (*M*,*P*) results in “*syn*” stereo form in which R_1–3_ and R_4–6_ groups (i.e., phenyl substituents) are positioned on the same side of the linkers. Conversely, “*anti*” diastereomers are generated upon connecting the CTB caps with the same helicity (*M*,*M* or *P*,*P*). In this case, the R_1-3_ and R_4-6_ groups reside on the opposite side of the linkers. If the three linkers are identical and derived from enantiomeric CTB units, then the *syn* isomer exhibits *C*_3h_ symmetry (achiral) while the *anti* isomer with *D*_3_ symmetry remains inherently chiral. The CTB moieties are reported to be flexible, thus they can invert or adopt a saddle-twist conformation at ambient temperature [138,139]. This feature of CTB resulted in considering the existence of four different cryptophane conformers, e.g., *out-out*, *in-out*, *in-in*, and *out-saddle* conformers. The existence of such cryptophane conformers are particularly dependent on the environment, i.e., the presence or absence of a guest inside its cavity. The different synthesized cryptophanes are often named alphabetically based upon the chronology of their synthesis due to an exceedingly complex naming system (when following systematic IUPAC nomenclature). Cryptophane-A (222-4) exists as *anti* isomer and is linked through ethylenedioxy linkers (-O(CH_2_)_2_O-) including the methoxy groups substituted at the CTB rims (R_1–6_ = OCH_3_) [134]. Cryptophane-B (*syn*-222-5) is the respective *syn* isomer of cryptophane-A and has been recently synthesized in lower yields, yet it binds Xe in 1,1,2,2-tetrachloroethane-*d*_2_ solvent [140] Cryptophane-C (222-6) and -D (*syn*-222-7) are typical modifications of cryptophane-A and -B, respectively, and they lack methoxy groups on one of the CTB units resulting in larger portals [135,141]. Cryptophane-E (333-9) and -F (*syn*-333-10) are connected via proplyenedioxy linkers (-O(CH_2_)_3_O-) along with methoxy groups substitutions at the CTB rims [136]. Similarly, the CTB units in cryptophane-I/J (444-13, *syn*-444-14) and -K/L (444-15, *syn*-444-16) are joined together through *trans* and *cis* butenedioxy linkers in addition to methoxy groups tethered at the CTB rims [142]. Different cryptophanes with varied internal cavity sizes were synthesized in order to entertain differently sized guests in solution.

Based on the different conformational possibilities and X-ray crystal structures, cryptophanes are regarded as somewhat flexible and their internal cavities must be dynamic in terms of internal volume to accommodate different guests. According to the crystal structure, the internal cavities are generally seen as spheroidal or oblong and the cavity volume somewhat scales with the length of the linkers. The cavity volume of cryptophane-A (CryA) is larger (*V*_cav_ ≅ 87–119 Å^3^) than that of its cryptophane-000 [143] and -111 [144] counterparts, albeit with a rather flexible structure. CryA and other cryptophanes are generally synthesized by employing one of the following approaches: (a) two-step, (b) template and/or (c) capping synthetic methods. CryA dissolved in organic solvents gave rise to solvent binding in the cavity which is explicitly shown through the observed binding constants, e.g., CH_2_Cl_2_@CryA: *K*_a_ ≅ 475 M^−1^ (298 K), CHCl_3_@CryA: *K*_a_ ≅ 230 M^−1^ (298 K), and CH_4_@CryA: *K*_a_ ≅ 130 M^−1^ (298 K), respectively [87]. Such solvent competition (e.g., in CHCl_3_) had impaired earlier studies in which the different guest encapsulations by CryA were studied. In general, solvent competition and solvation effects have to be duly considered prior to investigating molecular recognition properties of container molecules like CryA. To surpass the solvent binding effect and to enhance the binding capabilities for different guests, several structural modifications were performed on CryA. One of the earlier studies indicated that Xe binds remarkably to CryA (*K*_a_ ≅ 3000 M^−1^) in tetrachloroethane solvent. In this regard, structural modifications of CryA could help to achieve even better binding constants and it could also impart water solubility on the overall hydrophobic CryA. Such modifications were carried out to achieve an unsymmetrical phenolic cryptophane (e.g., introduction of allyl [16,145,146] and benzyl ether [147]), propargyl-incorporated CryA [148,149], propargyl-functionalized benzyl alcohol, and PEGylated CryA [150], respectively).

To make hydrophobic cryptophanes soluble in aqueous media requires attachment of hydrophilic residues, e.g., mono-, di-, tri- and hexa-acid derivatives of CryA. The solubility of such derivatives roughly correlates to the number of soluble residues tethered to the core structure and it also depends upon the solution pH. Other forms of water soluble cryptophanes are made available by introducing moieties such as peptides, dendrimers, PEG units and cationic transition metal units, respectively. It was found that aqueous complexes of phenolic CryA indicated that cations of suitable size (e.g., Cs^+^) could strongly bind to these highly anionic hosts. This strong binding is somewhat anticipated due to an isoelectronic configuration of Cs^+^ (Kr5s^2^5p^6^) in the outer shell similar to Xe. Direct determination of the Cs^+^ binding constant using ITC was inaccurate since the detection limit of the ITC was reached. Therefore, Cs^+^ binding to cryptophanes was determined indirectly through competition between Rb^+^ and Cs^+^ for cryptophanes. In this way, a very high binding constant was reported for hexahydroxy substituted- (*MM*)- and (*PP*)-cryptophane A derivatives with *K*_M_ = 6 × 10^9^ M^−1^ and 2 × 10^9^ M^−1^, respectively. This observation was mainly driven through the presence of phenolate moieties of CryA and by a strong Coulombic interaction between the phenolate moieties and Cs^+^, as well as the electrostatic interaction occurring between the cationic species and π-donor cavities of the host. Even though more than one Cs^+^ ion (*V* = 19.5 Å^3^) could very well fit inside the CryA cavity (*V*_vdw_ = ~95 Å^3^), only one Cs^+^ was shown to bind to CryA with high affinity. The greater the polarizability of the cation [151,152], the higher is the binding constant of the alkali metal cations, i.e., Cs^+^ >> Rb^+^ >> K^+^ > Na^+^ to hexahydroxy-substituted CryA derivatives [153]. 

Based on the insights regarding the cryptophane encapsulation abilities, the NMR biosensing interest in these hosts is based on their ability to bind Xe. Importantly, non-covalent complexation of Xe by CryA led to the achievement of ultra-low detection limits for ^129^Xe NMR-based biosensors and reporters. Among the different molecular containers that are known to bind Xe, cryptophanes are treated as a gold standard since they exhibit the highest Xe binding constant. Such high binding between cryptophane and Xe is often confirmed using isothermal titration calorimetry (ITC) measurements via the strongly positive entropy values noticed for Xe binding. For example, the triacetic acid cryptophane derivative (TAAC) revealed strongly positive entropy value (ΔS = 5.9 cal⋅mol^−1^⋅K^−1^ at 293 K), suggesting high affinity as temperature rises from RT to physiological conditions. Thus, ITC at 310 K indicated a high association constant of (33 ± 2) × 10^3^ M^−1^ for Xe-TAAC complexation [154]. The entropy and enthalpy values from ITC are anticipated to incorporate contributions from (a) dissolution of clathrate water structure that surrounds Xe in solution, (b) water release from hydrophobic CryA cavity, and (c) non-covalent dispersion interaction existing between Xe and CryA derivative [155]. These non-covalent contributions explain the enhanced Xe binding observed for CryA and its derivatives [144,149,154]. Also, the higher affinity of Xe for cryptophane is supposed to result from entropic (‘hydrophobic’) effects induced via desolvation of Xe clathrates upon complexation as shown through ITC findings. Such interaction was facilitated by CryA adopting more compact size in water, thereby promoting favorable van der Waals contacts with Xe [154]. The crystal structure of CryA with Xe as guest revealed that Xe is positioned near the optimal distance from CTG caps which is seen through van der Waals interaction distance of 3.86 Å between phenyl carbon and Xe [156]. CryA generates the packing fraction close to Rebek’s empirically determined value by adopting intermediate volume fraction for Xe leading to favorable balance between entropic and enthalpic forces that contribute to observed high Xe affinity. The flexible ethylenedioxy linkers, although existing in both *gauche* and *anti* conformations, adapt according to the incoming hydrophobic guest volume such that the cavity volume could be increased up to 20%, thereby enabling inclusion complexation. X-ray crystal data confirms that Xe encapsulation by CryA is mainly driven through different hydrophobic and non-covalent interactions [157].

Cryptophanes with smaller, spheroidal cavities (*n,m,l* ≤ 3) are well suited for encapsulating Xe with slow exchange on the ^129^Xe NMR chemical shift timescale. This is a prerequisite for certain sensing applications. For example, Xe@CryA observed by ^129^Xe NMR in (CDCl_2_)_2_ demonstrated a slow in-out exchange of Xe as a consequence of constrictive binding and a chemical shift (~62 ppm) in addition to the binding constant of *K*_a_ = 3.95 × 10^3^ M^−1^ at 278 K [158]. The binding constant of Xe in (CDCl_2_)_2_ for different cryptophanes (*n,m,l* ≤ 3) at 278 K are 28000 M^−1^ (Cry111-1), 3900 M^−1^ (Cry222-4), 1400 M^−1^ (Cry222-8), 2810 M^−1^ (Cry223) [158] and similarly for water soluble cryptophane congeners (hexa-acid functionalized, 293 K): 6800 M^−1^ (Cry222-67), 2200 M^−1^ (Cry223-71), 1600 M^−1^ (Cry223-72) in D_2_O, respectively. Along this line, Dmochowski and coworkers reported a binding constant of 4.2 × 10^4^ M^−1^ for a series of tri-functionalized 222-core cryptophanes (222-77, 222-42, 222-43). The observed differences are attributed to the average number of water molecules displaced from the cavity upon Xe binding. Xe binding constant determinations are influenced by different factors such as the solvent, cations, counter-cations, ionic strength, molecular dipoles, and exterior functional groups etc. Additionally, the presence of imploded cryptophane conformations in solution might significantly hinder the accurate estimation of binding constants [159]. For example, calorimetric methods attributed to guest complexation enthalpies may be altered due to convolution of enthalpy of *saddle* to *crown* conversion and the enthalpy of complexation. 

Introduction of additional methoxy groups on the cryptophane backbone has a strong impact on the in–out exchange dynamics of xenon albeit the lengths of the linker are the same. In this context, a series of cryptophane derivatives that differ only by the number of methoxy groups attached to their phenyl rings were synthesized (compounds **6a–s**, **9a–s**, **12a–s**, where a and s denotes *anti* and *syn* arrangement of the linker). All these compounds are shown to bind xenon. The introduction of methoxy substituents positioned on each side of the linker rigidifies the cryptophane-222 scaffold. Thus, these compounds display several conformations of their bridges with slow exchange dynamics in solution. This effect seems more pronounced with the more rigid *syn* derivatives. Conversely, *anti* derivatives with higher flexibility reveal a major conformation for the linkers after the introduction of xenon. The Xe exchange dynamics is found to be strongly dependent on the degree of substitution. ^1^H-NMR spectra of these derivatives in solution show the presence of different conformations in the slow exchange regime that is explained via a decrease of the flexibility of their skeleton. Using ^129^Xe-^1^H dipolar cross-relaxation (SPINOE) spectra, it was demonstrated that a single conformation is present in the solution that can bind xenon. Thus, these results demonstrate that cryptophane derivatives possessing identical linkers can show very different properties towards xenon encapsulation [160]. 

The chemical shift of the ^129^Xe nucleus is highly sensitive to its immediate environment which also holds true for Xe encapsulated by container molecules like cryptophanes. It was shown that there exists a modest temperature dependence of the Xe chemical shift which is inversely correlated to the overall rigidity of the cage [158]. The solvent effects on encapsulated Xe are limited due to its shielding inside the cavity. While Xe@CryA appears at 62 ppm in (CDCl_2_)_2_ at 278 K, water soluble hexa-acid Cr222-67 resonates at 64 ppm in D_2_O at 293 K, and the PEG-modified counterpart (Xe@Cry222-39) exhibits a peak at 77 ppm in H_2_O, respectively. It was also reported that an increase in linker length and volume of cryptophane led to a decrease in chemical shift observed in H_2_O at 293 K. The hexa-acid substituted cryptophane complex Xe@Cry222-67 appears at 64 ppm, Xe@Cry223-71 at 52 ppm, Xe@Cry233-72 at 42 ppm, and Xe@Cry333-68 at 35 ppm, respectively [161]. Since the Xe@CryA chemical shift range is relatively narrow (Δδ = 16 ppm) and Xe-binding cryptophanes are limited, there is a need for installation of chemical shift modifiers (e.g., functional groups) onto its structure. The flexibility and length of the tether might have a strong influence on the observed Xe NMR line width and the chemical shift sensitivity of the bound nucleus [162,163]. The functional groups appended onto the arenes of the cryptophanes are the ones which affected the chemical shift dramatically, e.g., ^129^Xe@[(Cp*Ru)_6_(Cry111-1)]Cl_6_ resonated at 308 ppm at 293 K in D_2_O with an extraordinary 277 ppm downfield shift from the ^129^Xe@Cry111-1 resonance in (CDCl_2_)_2_ [164,165]. 

With the advent of HyperCEST, the exchange behaviour of Xe with its hosts became even more important than the binding constant. Zeolites in suspension, e.g., allow loading with a relatively large amount of Xe from the dissolved pool but the gas has a rather long residence time inside the pores [166]. While this behaviour is useful for providing a micro-environment with convenient relaxation conditions (*T*_1_ ~30–40 s), the long residence time makes this carrier useless for HyperCEST applications. The kinetics of guest exchange (ingress/egress) with the host was ascertained by utilizing various NMR methods, including line width analysis, inversion recovery or exchange spectroscopy (EXSY) experiments. The guest kinetics is proposed to be dependent upon the way in which the guest binds (e.g., constrictive binding) and on the energy barrier challenges which it has to endure upon binding to the host. It is noteworthy that a spheroidal container like cryptophane and other molecular containers are known to indulge in generating a significant kinetic barrier to the ingress/egress of guests in solution [167]. The egress of guests is reported to undergo two types of “gating” mechanisms that regulate its exchange. These are called “French door” and “sliding door” mechanisms, respectively. The “French door” mechanism involves the opening of host portals via a high barrier conformational change, thereby promoting guest exchange. The constrictive binding energy in this case is usually dependent entirely on the host structure. Another plausible exchange mechanism (“sliding door”) is similar to squeezing the guests through the flexible portals via the changes in linker conformation. The sliding door mechanism is mainly driven by the nature of the guest and the host structure [168]. In terms of cryptophanes, the methoxy groups that line the CTB rim could effectively swing out of the arene ring planes to open the gate for guest entry. Conversely, linkers at the cryptophanes are somewhat flexible, thereby offering almost a sliding type door for guest ingress/egress. The constrictive binding is the main factor that governs the kinetic stabilities of cryptophane complexes in solution, i.e., slow exchange of guests on NMR time scale. It is shown that CryA complexes are kinetically stabilized by constrictive binding effects albeit having an affinity for very small sized guests. For example, constrictive binding energies of Xe@CryA and CH_4_@CryA complexes (34 and 32 kJ mol^−1^, respectively) in (CDCl_2_)_2_ are much higher compared to intrinsic thermodynamic stability (19 and 11 kJ mol^−1^), respectively [169].

Although a high Xe binding constant will be critical for sensing applications, the Xe in/out exchange rate plays a major role in applications that aim for an ultra-low detection limit such as HyperCEST experiments. To achieve an optimal HyperCEST contrast, long *T*_1_ relaxation times are desired. However, detection based on analyte effects on the *T*_2_ relaxation of reversibly bound Xe is also known [170]. The Xe exchange rate is known to follow two different types of exchange mechanism, namely dissociative type (a supramolecular *S*_N_1-type) and/or a degenerate, associative (*S*_N_2-like) mechanism, respectively. The associative mechanism depends on the host and Xe concentration, and becomes a relevant contribution for high host concentrations (the amount of dissolved Xe is assumed to be fixed through the Ostwald solubility coefficient). The dissociative counterpart does not reveal the extent to which the solvent may be involved in the Xe expulsion. A systematic study of the Xe release rates from different cryptophanes correlated to the diameter of the portal of the cryptophane cages in organic solvents, e.g., follow the order Cry222-8 (R^1–6^=OCH_3_) > Cry222-6 (R^1–3^=OCH_3_, R^4–6^=H) > Cry222-4 (R^1–6^=H). Interestingly, Xe NMR line widths of Xe@Cry222-4, Xe@Cry223-24, Xe@Cry233-23, Xe@Cry224-25 and Xe@Cry333-9 indicate that at low pressure the exchange is faster for smaller cavities than larger ones and at high pressure a reverse situation was observed [171]. Other hosts with faster exchange times have been investigated in several studies and are further discussed in Section 5.8 and in Table 1 and Table 2. 

### 5.2. Peptide Chemistry

**Figure 7:**
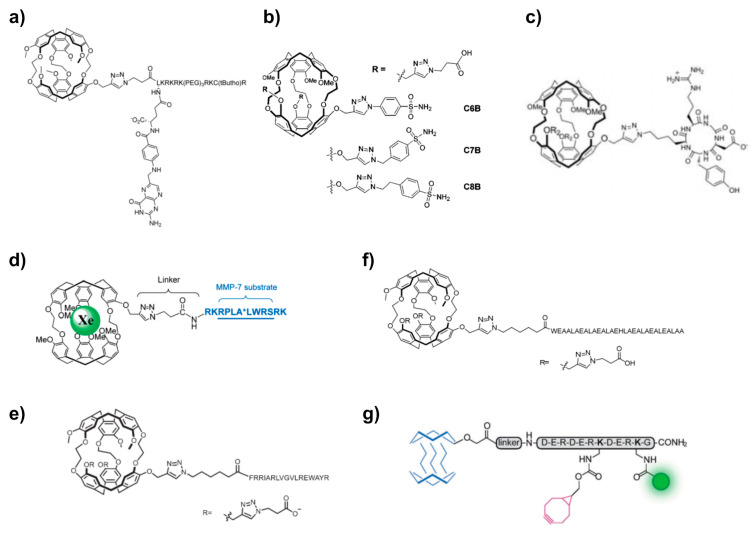
Exemplary cryptophane-A-based targeted ^129^Xe biosensors discussed in this review. These biosensors were developed for investigating the following cellular targets: (**a**) folate [176] (**b**) carbonic anhydrase [177] (**c**) integrin [127] (**d**) matrix metalloproteinase-7 [148] (**e**) calmodulin [178] (**f**) cellular pH change response [179], and (**g**) glycans [128], respectively. Figures reproduced with permission from respective journal publishers.

One of the limitations of hosts from the cryptophane family is their poor solubility in water. To improve this aspect, the above-mentioned version with several solubilizing Ru complexes had been introduced [165]. However, a metal free version is favourable for biological applications and was later demonstrated in different approaches [172,173]. Attaching peptidic units to CryA was used as a measure to improve solubility irrespective of further functionalization of the sensor. For instance, a water solubilizing tetra-peptide, Lys-Glu-Glu-Glu-Glu, was chosen to investigate Xe@CryA as a temperature sensor in MR thermometry [174,175]. The functionalization of cryptophanes for binding to selected targets has actually also been performed in many cases with peptide chemistry protocols. An overview of different concepts is given in Figure 7, with a more detailed discussion in the following sections. 

#### 5.2.1. Avidin-Biotin Systems 

Avidin, a protein majorly found in both avians and amphibians, displays high affinity towards its cofactor biotin that drives multiple biological processes. The avidin-biotin interaction is one of the strongest non-covalent bindings (*K*_D_ ≅ 10^−15^ M) occurring via avidin’s ability to bind four biotins under biological conditions. Once formed, these interactions are unaffected by the influence of pH, proteolytic enzymes, temperature, organic solvents and other denaturing agents [180,181,182,183]. The avidin-biotin systems find applications in biochemical assays, affinity purification, immunohistochemistry, ELISA, immunoprecipitation, cell surface labeling and FACS, respectively [184]. Supramolecular adducts formed between avidin and biotinylated Gd(III) chelates somewhat improved the ^1^H MRI-based sensitivity issues: dimeric biotinylated Gd(III) cross-links adjacent avidin and yields increased relaxivity but leads to the formation of multilayered adducts and complex mixtures of oligomers with different Gd payload [185]. These limitations from ^1^H MRI-based probes were addressed through the generation of a CryA-based biosensor for detecting the biotin-avidin interaction at relatively low concentrations. To do so, this biosensor prototype (B1) comprised three units, i.e., the host CryA-ma, the targeting ligand (biotin), and a linker that was generated in addition to the attachment of a short peptide (CysArgLysArg) for promoting solubility. This biosensor B1 showed four ^129^Xe resonances owing to its four different diastereomers *RLL*, *RLR*, *LLL* and *LLR* in solution. However, the solution containing both the biosensor (300 µM) and avidin as the analyte (≅80 nmol) exhibited only three peaks at 193 (Xe@aq), 70 ppm (Xe@CryA) and at 2.3 ppm downfield to the bound Xe peak that was assigned to Xe in the avidin-bound sensor. Pre-saturation of avidin with biotin followed by biosensor administration indicated no observable peak for sensor-avidin interactions. Without avidin pre-saturation, the diastereomers signals overlap and produce only two Xe resonances. However, complete saturation of B1 with avidin indicated only a single broad peak [16].

The influence of linker lengths that connect the CryA cage to biotin was investigated by generating five different biotinylated Xe biosensors with variable linker lengths. Additionally, such an approach was beneficial in identifying the molecular properties that promote optimal line width and higher sensitivity for the detected biosensor. If the attached linker is short, it might restrict the CryA cage motion due to its proximity to the protein surface, thereby increasing the observed chemical shift. The second biosensor, B2, was generated by replacing the cysteine maleimide linkage in B1 by a lysine. B2 (140 µM) exhibited only two diastereomers namely *RL* and *LL*, respectively. Addition of the avidin tetramer (29 µM) to B2 resulted in a downfield shift of two diastereomeric peaks at 65.1 and 64.5 ppm to 67.9 and 66.4 ppm, respectively. Another biosensor B3 similar to B2 but with a different rigid linker (six glycines) revealed two narrow peaks with line widths of 35 and 24 Hz (1 ppm separation) due to its *RL* and *LL* diastereomeric forms. Addition of avidin (20 µM) to B3 (77 µM) showed four peaks with large line widths (45–55 Hz) and of unequal intensities. Next, biosensor B4 was generated with a short linker and led to the increase of both line width and chemical shift sensitivity upon avidin binding. However, binding of biosensor B5 (CryA tethered to 12 PEG units) resulted in no chemical shift change. These findings indicate that the linker length significantly influences the line width and chemical-shift sensitivity of such biosensors upon protein binding [162]. ^129^Xe NMR of immobilized avidin-agarose beads (ca. 250 µL) treated with biosensor B3 and supplied with a continuous flow of H_2_O saturated with hp Xe revealed three peaks at 65.4 (Xe@B3), 193.6 (Xe@agarose), and 192.5 ppm (Xe@aq), respectively. Conversely, ^129^Xe chemical shift imaging indicated image intensity of Xe@aq (≅193 ppm) spanning over the entire sample space while the Xe@B3 (65.4 ppm) image intensity was localized only around the actual volume of the beads. These results indicate that MR imaging of targeted Xe biosensor distribution could be accomplished with good sensitivity due to the hp nuclei [163].

As one of the applications based on the avidin-biotin approach, our lab combined a biotinylated CryA with an avidin-labeled anti-CD14 antibody such that a modular biosensor was generated for imaging CD14 targets in live cells. CD14 is a glycosylphosphatidylinositol-anchored protein (55 kDa) expressed on myeloid cell surfaces [186,187] and it also serves as a well-characterized macrophage marker. The modular biosensor consists of “targeting” and “readout” modules. The targeting module is generated by conjugating multiple avidin units to an anti-CD14 IgG2b monoclonal or to an IgG2b control antibody. The readout module comprised a CryA monoacid (CryA-ma) that was conjugated to biotin via a PEG spacer. This can be combined with a biotinylated fluorescent dye for complementary optical detection. The versatility of this modular approach was checked by either sequentially adding the targeting and readout modules to cells in separate incubations or by a single incubation of the cells with the complete biosensor containing both readout modules. A more refined quantification of CryA was achieved by using a “branched readout module” where a CryA diacid is conjugated to both biotin and fluorescein via the PEG spacers. The flow cytometry data showed that the sequential addition of two modules (4:1 CryA:fluorescein) promoted quantification of cells labeled with CryA indirectly through fluorescein. The fluorescence microscopy images of RAW 264.7 (CD14(+)) cells indicated that the CD14 biosensor is predominantly localized at the cell surface. The ^129^Xe NMR analysis of RAW264.7 (CD14(+)) and 3T3 (CD14(−)) cells incubated with CD14 biosensor showed a resonance (~120 ppm) only for RAW 264.7 cells. The ^129^Xe MRI and fluorescence measurements illustrated that the antibody-based CD14 biosensor was able to delineate the RAW 264.7 cells from 3T3 cells under different incubation conditions. Thus, the antibody-based modular biosensors approach demonstrates that they can be delivered separately with reduced toxicity and it also enables faster and specific labeling of targets of interest [15].

The modular avidin-biotin approach was further extended to investigate the claudin family using targeted Xe biosensors. Claudins (Cldn) are tetraspan transmembrane proteins which are the main constituents of tight junctions (TJ) [188]. Their localization outside TJs is presumably linked to tumorigenesis [189]. *Clostridium perfringens* enterotoxin (CPE) allows to derive a targeting unit as it binds with high affinity to Cldn3, -4, -6, -7 and -9 and with moderate/low affinity to other Cldns. CPE is released by *C. perfringens*-type A strains and it is a β-pore forming toxin that causes food poisoning. Contrarily, the C-terminal domain of CPE (cCPE) alone is shown to be devoid of toxicity both in vitro [190] and in vivo [191]. Nonetheless, cCPE was shown to preserve its specific binding to Cldns [192,193]. The Cldn3/4-targeted Xe biosensor based on the modular platform promotes detection with superior sensitivity for such a membrane-bound protein target. For this study, avidin was conjugated to lysine residues of the targeting module, i.e., the mutant GST-cCPE-K257A, via a linker followed by tethering it to biotin-fluorescein and/or biotin-CryA readout modules. Flow cytometry of HEK cells transfected with Cldn4-FLAG and parent HEK cells incubated with the Cldn4 biosensor (160 nM) revealed 48-fold higher fluorescence for Cldn4 transfected HEK cells than non-transfected counterparts. The ^129^Xe HyperCEST MRI showed a median of 59% CEST effect for transfected HEK cells compared to 11% pertaining to the non-transfected cells. A unique peak for Xe@CryA, i.e., in a lipidic/cellular environment (~120 ppm) could be observed. Thus, this approach demonstrated that a significantly lower expressed cellular target can be efficiently detected using the highly sensitive HyperCEST method while such targets normally remain inaccessible to relaxivity-based MR agents [194].

#### 5.2.2. SPPS-Based Approaches

As discussed earlier, hydrophobic cryptophanes are rendered water soluble by installation of certain functional groups onto their structure. This is mostly achieved by tethering peptides of different length and charge onto the handles available at the cryptophanes. The peptides are synthesized using solid phase peptide synthesis (SPPS) technique in which appropriate amino acids are assembled onto a resin using standard peptide coupling reagents under basic conditions in DMF solvent. In some cases, the cryptophanes are attached to a side chain (e.g., lysine) of the peptide while it is still anchored onto the resin. In other approaches, the final peptides are cleaved from the resin and the coupling between the cryptophanes and the peptide aims for a better coupling efficiency and possibly higher yields of the Xe biosensors in solution. Usually, a fluorophore tag will be tethered in addition to the cryptophane such that the sensitivity and selectivity of the Xe biosensors could be validated by using both Xe NMR/MRI and fluorescence measurements. Mostly, Xe biosensors are synthesized by using CryA-ma in which the monoacid handle can be functionalized with peptides, linkers, and targeting moieties, respectively. Cryptophanes with two acid handles (CryA-da) are also employed to tether different moieties of interest onto either side of it. Tri- and hexa-functionalized CryA have been developed to foster its solubility in aqueous media. The generation of Xe biosensors involving both SPPS and solution-based coupling led to different synthetic hurdles as described above and resulted in a non-uniform and undesired functionalization of the host. To avoid such issues, a model biosensor was synthesized entirely on the resin, therefore a compact biosensor will be attained without any side products and with a reduced number of purification steps. 

**Figure 8:**
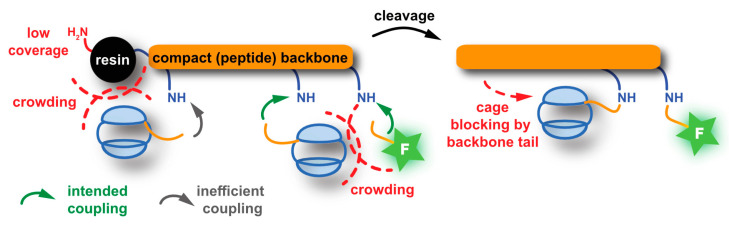
Generation of hosts for functionalized Xe on the solid support. Possible challenges (highlighted in red) include limited reactivity at intended coupling sites due to crowding at side chains when attaching bulky functional units such as CrA cages (blue) or fluorescent dyes (green); low coverage of the resin might be inevitable if coupling of such units is attempted too close to the resin; blocking of the cage through the peptide tail might occur after cleavage. Reprinted with permission from *Bioconjugate Chem.* 2018, 29, 12, 4004–4011 [195]. Copyright (2018) American Chemical Society.

The complete generation of Xe biosensors on the solid support (e.g., a resin) is beneficial in avoiding so-called “crowding effects” arising due to the introduction of CryA at the amino acid residue linked to the vicinity of the highly loaded resin. It also helps to prevent the steric or crowding effects occurring due to the presence of the CryA moiety in proximity to bulky substituents like a fluorophore tag. Another issue that should be avoided is the potential back-folding of the N-terminus of long peptides into the cavity of CryA attached to the amino acid side chain in solution. This might prevent the entrance of Xe into the host, thereby leaving no possibilities for Xe-based NMR sensing (see Figure 8). Our lab pursued the re-synthesis of a CryA-da-based Xe biosensor entirely on the solid phase. The biosensor comprises CryA-da, fluorescein, and an exemplary targeting unit (e.g., biotin) connected via the linkers that were originally combined in solution. The earlier solution-based approach led to reduced overall yield (~10 %) in addition to the generation of functionalized CryA-da with mixed labels [15]. Three different approaches were tried out to generate a successful Xe biosensor completely on the solid state. Firstly, the synthesis of the model biosensor biotin-(PEG)_3_CH_2_CH_2_-(CryA-da)-(PEG)_3_CH_2_CH_2_-Lys(5/6-FAM)-Gly was attempted by joining two sub-components of the biosensor on the solid phase. Unfortunately, the biosensor was not achievable due to less surface coverage of the highly loaded resin that occurred due to an inevitable administration of low molar equivalents of certain building blocks during the peptide synthesis. The final coupling did not proceed to completion in spite of prolonging the reaction time. 

With the insights learned from first coupling procedure, the synthesis of the second model biosensor, biotin-(PEG)_3_CH_2_CH_2_-(CryA-da)-(PEG)_3_CH_2_CH_2_-Lys(5/6-FAM) was attempted entirely on the solid support as it fosters the possibility of introducing the CryA-da directly into the growing peptide chain. In this approach, our lab utilized low loaded Tentagel^®^ RAM resin and different components of the biosensor were sequentially tethered onto the resin. Notably, the coupling of CryA-da onto the preceding amino acid was achieved by providing sub-millimolar equivalents of the former one during coupling. Hence, cross-reactivity between the CryA-da and resin and or other amino acids was avoided. After NHS activation of the carboxyl group at CryA-da, excess amine (biotin-(PEG)_3_-CH_2_CH_2_-NH_2_) was administered to facilitate coupling under alkaline conditions. Then, fluorescein was tethered at the lysine side chain by an extended peptide coupling procedure. This second approach enabled successful synthesis of the entire Xe biosensor on the resin with an overall yield of ca. 20%. Thirdly, to demonstrate exclusive side chain approach for introducing the Xe host, CryA-ma was employed during the synthesis of the model biosensor acetyl-Glu((PEG)_3_biotin)-Lys(CryA-ma)-(PEG)_3_CH_2_CH_2_-Lys(5/6-FAM)-Gly. Our lab attempted the synthesis using low loaded Tentagel^®^ RAM resin as above and synthetically proceeded successfully until the steps prior to CryA-ma coupling at the side chain. Just to avoid cross-reactivity of cage at the glutamic acid residue, acetylation of the free amine was performed prior to Dde protecting group removal from lysine side chain which is installed as the precedent amino acid to glutamic acid. Even after repeating the coupling and prolonging the reaction time, it was noted that the amine at the lysine side chain is unavailable for further coupling with CryA-ma. This observation could be attributed to the hopping nature of the Dde group among different free amines and to the acetylation procedure. To surpass this issue, lysine with different protective groups could be employed during the Xe biosensor synthesis. 

The ^129^Xe HyperCEST NMR response of the second model biosensor was evaluated in H_2_O plus 1% DMSO with reference to the naked CryA-ma under similar experimental conditions. The ^129^Xe HyperCEST NMR indicated a CEST peak at −131.2 ppm for the biosensor compared to CryA-ma CEST resonance at −132.4 ppm, respectively. The HyperCEST response of the biosensor was somewhat reduced which could be attributed to the attachment of different units in close proximity albeit showing Xe exchange rate similar to that of CryA-ma. Subsequently, ^129^Xe HyperCEST MRI of the above biosensor under similar experimental conditions confirmed its ability to produce MR images with a reasonable contrast compared to the CryA-ma reference. This demonstrated that the entire Xe biosensor could be successfully synthesized on the solid support under appropriate peptide synthesis conditions such that host like CryA-da could be introduced onto the growing peptide chain without any undesired side products and peptide-cage interactions, respectively [195].

### 5.3. Click Chemistry Approaches

The term “click chemistry” was originally coined by Sharpless and co-workers [196] to address a series of powerful, highly reliable, and selective reactions for the rapid synthesis of new compounds and combinatorial libraries, respectively. Generally, such a click chemistry approach must be modular, wide in scope, give very high yields, generate inoffensive by-products (if at all) that can be removed by non-chromatographic methods, and should remain stereospecific. The characteristics of such processes include simple reaction conditions, readily available starting materials and reagents, and simple product isolation, respectively [197]. One of the most widely studied reactions under click chemistry is Cu(I)-catalyzed Huisgen 1,3-dipolar azide-alkyne cycloaddition (CuAAC) resulting in 1,2,3-triazoles as product [198]. The copper(I)-catalyzed reaction is mild and very efficient without the prerequisite for protecting groups and no purification under most reaction conditions [199,200]. The azide and alkyne functional groups remain inert towards biological molecules and aqueous environments, thereby promoting Huisgen 1,3-dipolar cycloaddition in target-guided synthesis and activity-based protein profiling [201,202]. Unlike amides found in nature, the triazole is not susceptible to cleavage, oxidation and reduction, respectively. Because use of Cu(II) salts with ascorbate is problematic during bioconjugation, TBTA (tris[(1-benzyl-1H-1,2,3-triazol-4-yl)methyl]amine) has been employed as a replacement to achieve enhanced copper-catalyzed cycloaddition without having any detrimental effect on the biological scaffolds [203]. The CuAAC cycloaddition finds applications in classical organic/combinatorial synthesis, medicinal chemistry, drug discovery, polymer/material science, and in chemical biology, respectively [204]. Additionally, the CuAAC reaction is also employed in synthesizing catenanes and rotaxanes to achieve complex structures, supramolecular complexes, and functionalization of existing structures like container molecules [205]. 

In the context of Xe biosensor synthesis, click chemistry was utilized as a valuable tool in introducing an alkyne or azide as a handle onto CryA during its generation from two CTG units via the linkers [149,200]. The overall yields of such click chemistry-based introduction of units such as, e.g., alkynes while forming the CryA is less (3–5%) compared to other approaches. However, CryA with an alkyne unit is useful for clicking it to an azide-containing moiety (e.g., peptides, fluorophore etc.). The click chemistry between derivatized CryA and appropriate azide moieties is facilitated by Cu(I) catalyzed Huisgen-based 1,3 cycloaddition to yield a respective triazoles linked Xe biosensors. CryA was either mono- or tri-derivatized with propargyl groups (alkyne) such that it could be easily anchored to an azido moiety. The monopropargyl derivative of CryA was synthesized in 12 nonlinear synthetic steps, leading to 3% overall yield [148]. An improved yield of 49% was attained if the final cyclization of the monopropargyl-derivatized CryA was performed in a perchloric acid/MeOH (50:50) mixture. The Huisgen 1,3 cycloaddition of the monopropargyl derivative of CryA to azido peptides anchored onto the solid support was performed by using CuSO_4_, sodium ascorbate and a base (e.g., 2,6-lutidine) either in aqueous conditions or in DMSO at room temperature to yield approximately 80% overall yields in the presence of large excess of sodium ascorbate (40 equiv.). The monopropargyl derivative of CryA was successfully coupled to azido peptides to generate different Xe biosensors for the detection of matrix metalloproteinase (MMP) enzyme activity [148], cell penetrating peptides capabilities [127] and highly expressed folate [176] receptors in different cancer cells, respectively. In terms of MMP activity, the ^129^Xe NMR of uncleaved intact MMP-7 biosensor in D_2_O indicated signals from two diasteromers (*RL* and *LL*) at 61.8 and 62.4 ppm, respectively. The mixture of uncleaved and cleaved MMP-7 biosensor yielded two groups of two peaks each, separated by 0.6 and 0.8 ppm, respectively. In the case of the folate biosensor, two peaks at 64.8 and 66.0 ppm were observed in acetate buffer (pH 5) due to its diastereomeric forms (*LRL* and *LRR*) arising due to the chirality contributions from folate, peptide, and cryptophane. These ^129^Xe NMR-based studies show that upon successful usage of click chemistry the as-generated biosensors were able to delineate their respective targets of interest via the chemical shift change under aqueous conditions.

Dmochowski and co-workers have synthesized three different derivatives (propargyl, allyl, benzyl) of tri-substituted CryA in six steps (~4–5% yield) by using commercial starting materials. The protecting groups (e.g., propargyl, allyl, benzyl) on CTG caps can be deprotected to yield tri-hydroxy CryA derivatives, where the latter might be used for installing targeting moieties (e.g., peptides). Assessing the cavities of tripropargyl-, triallyl- and tribenzyl-CryA derivatives using hp ^129^Xe NMR showed chemical shifts between 63 and 65 ppm while a signal appeared at 57 ppm for the trihydroxy-CryA derivative. This difference in chemical shift between the trihydroxy- and other tri-substituted CryA is somewhat attributed to the larger portals of trihydroxy-CryA derivative [147]. 

The tripropargyl derivative of CryA was synthesized in six nonlinear synthetic steps with an overall yield of 6.4% while the yield for a strategy with five linear steps was 9.9%. Monopeptidic CryA was obtained preferentially by controlling the reaction stoichiometry between tripropargyl CryA and azido peptides, e.g., 1:0.8 of CryA to azido peptides. The click reactions were achieved by using either CuBr or CuSO_4_ along with TBTA, base, and sodium ascorbate in organic solvents at room temperature to generate a copper(I)-catalyzed [3+2] azide-alkyne cycloaddition (CuAAC) product. The triazole-hexyl spacer resulting from click chemistry is beneficial in maintaining the peptides in close proximity to the CryA-bound ^129^Xe nucleus while reducing the potential steric hindrance with CryA during conjugation. The monopeptide CryA synthesized via click chemistry was obtained with an overall yield of 60–80%. To make the biosensor more water soluble, a solubilizing linker such as 3-azidopropionic acid was treated with crude monopeptide CryA via a second CuAAC to yield Xe biosensors with two propionic acids and respective peptides in yields approximately between 40–70%. This approach was used to synthesize enantiopure trifunctionalized cryptophanes for various applications [206], including pH-dependent cell labeling of cancer cells [179], targeting integrins expressions in cells [127], sensing human carbonic anhydrase (CA) I and II enzymes [177,207,208,209] and monitoring Ca(II)-triggered peptide binding to calmodulin [178], respectively. 

The pH biosensor had a variable ^129^Xe NMR chemical shift between 64.2 ppm (pH 7.5) and 67.6 ppm (pH 5.5) in solution at room temperature that was mainly facilitated by reversible peptide (Trp)-cryptophane interactions. Upon treating HeLa cells (1 × 10^7^ cells/mL) with this biosensor (5–10 µM), a CEST peak was observed at 78.4 ppm for pH 5.5 and at 65.0 ppm for pH 7.5. Notably, this large downfield shift occurs due to an insertion of the biosensor into the cell membrane at pH 5.5 compared to the condition with free sensor at pH 7.5. 

The ^129^Xe NMR spectrum of an integrin biosensor in tris buffer (1 mM, pH 7.2) showed only one resonance at 65.8 ppm in spite of the presence of two enantiomers. Addition of α_IIb_β_3_ (16 µM) to the biosensor (50 µM) yields two resonances at 67.1 ppm (free CryA) and 71.2 ppm (bound CryA). Observing a single bound resonance was attributed to the presence of a short alkyl spacer between the cyclic peptide c[RGDyK] and CryA, leading to a similar mode of interaction for both biosensor racemates at the integrin receptor. 

A water-soluble biosensor targeting the enzyme carbonic anhydrase (CA) was developed without the induction of an additional stereocenter onto the CryA core. Installation of the arylsulfonamide ligand on the biosensor allowed comparison of biosensor-CA interaction to other CA inhibitors. The cryptophanes were attached via the triazole linkers with 6-, 7- and 8-bonds to benzenesulfonamide (C6B, C7B, C8B) ligand. The shortest biosensor (C6B) was reported to fit the protein cleft leading to positioning of CryA at the edge of the protein surface. The longer versions C7B and C8B were generated to modulate the interaction between the protein and CryA and to analyze the resulting changes in ^129^Xe NMR chemical shift. C6B, C7B, and C8B showed peaks for bound Xe at 63.5, 63.9 and 62.9 ppm, respectively. Addition of CAI or CAII to C6B resulted in two peaks, namely one for bound and one for unbound biosensors. Conversely, treatment of CAI or CAII with C8B indicated three peaks, i.e., two bound C8B enantiomers and one unbound, respectively. Interestingly, C7B interaction with CAI indicated two peaks (one bound and one unbound) and C7B with CAII revealed three peaks similar to C8B. 

In the case of a calmodulin (CaM) biosensor (FRRIAR-TUC), the compound was generated by tethering a 17-mer truncated peptide (i.e., FRRIAR that enabled CaM binding) to CryA via click chemistry. ^129^Xe NMR of FRRIAR-TUC in CaM binding buffer gave no NMR signal for caged Xe. However, addition of equimolar *halo* form of CaM changed the conformation of FRRIAR-TUC in CaM binding buffer such that two peaks for bound Xe (65.9, 67.6 ppm) could be observed. These were assigned to diastereomers formed due to conjugation of racemic CryA to chiral peptide. Conversely, no Xe signal was observed for FRRIAR-TUC in presence of the *apo* form of CaM, nor for the *halo* form of CaM in the presence of FRRIAR alone. All these above findings show the usefulness of tri-functionalized (tri-propargyl) CryA in generating Xe biosensors with higher yields for investigating a target of interest or for monitoring the activity of an enzyme. 

As an alternative short synthetic route for generating water-soluble tri-substituted CryA derivatives, Rousseau and co-workers introduced polyethylene glycol (PEG) during the synthesis of cyclotriveratrylene (CTV) caps. The PEG groups improve solubility of CryA intermediates and reduce the number of steps by avoiding protection/deprotection of the starting material. Firstly, tosyl-functionalized PEG monomethyl ether with eleven repeating units was synthetically introduced to the starting material vanillyl alcohol, followed by Sc(OTf)_3_-mediated trimerizeriation of monoPEG derivative of vanillyl alcohol leading to the generation of CTV units in good yield. Removal of the methoxy group from PEG-substituted CTV using lithium diphenylphosphide gave the desired phenolic hydroxyl groups without degrading the PEG groups. Further, the propargyl derivative of the benzyl alcohol was utilized to form the counterpart CTV cap which is finally cyclized to yield CTV with PEG groups. The final PEGylated tri-propargylated CryA derivative (PEG-CryA) displayed the highest solubility in H_2_O (20 mM). Additionally, the propargyl moieties promotes Huisgen cycloaddition with organic azide, thereby opening avenues for installing different moieties (targeting motifs, fluorophores, etc.) and for grafting it onto macromolecular scaffolds (e.g., dendrimers, bacteriophages). Direct hp ^129^Xe NMR of the PEG-CryA in H_2_O showed a peak at 77.4 ppm for caged Xe. Thus, this synthetic route serves as a modular approach in which different PEG-CryA derivatives can be synthesized (e.g., hexaPEG CryA, triPEG CryA) by using an appropriate starting material for the generation of different CTV caps [150].

Click chemistry can also be used for the in situ binding strategy that the sensors will undergo with the molecular target of interest. This was demonstrated for tethering the alkyne group attached on the peptide backbone of a biosensor to azide groups exposed in the glycan structure of metabolically labeled cells such that the cell surface glycans could be investigated using ^129^Xe HyperCEST NMR and MRI. Glycans are known to be actively involved in cell-cell interactions, virus-host interactions, embryonal development, cancer cell metastasis, and immune function [210,211,212,213]. A multimodal biosensor was synthesized consisting of three functional units, namely the Xe host (CryA), a bioorthogonal targeting unit (bicyclo[6.1.0]nonyne (BCN)), and a fluorophore (fluorescein), respectively. These three units were anchored onto a peptide scaffold (DER repeat peptide) along with a PEG linker for achieving water solubility and for reducing non-specific binding of the biosensor. Metabolic incorporation of azides into cell surface sialic acids was achieved using Ac_4_ManNAz [213,214,215], followed by bioorthogonal labeling of resulting azido glycans with the biosensor. Fluorescence microscopy indeed revealed staining of the cell surface by the biosensor while the control cells displayed only lower and punctuated staining. As both CryA and BCN are known to induce nonspecific uptake, different conjugates were synthesized similar to the biosensor but without BCN attachment or without CryA and also one compound missing both BCN and CryA for studying the influence of these units on the biosensor specificity. Our lab found that each functional unit moderately increased the unspecific binding. However, the BCN-bearing conjugate exhibited sufficient specificity towards metabolically labeled cells than control cells. Cells treated with both the biosensor and Ac_4_ManNAz generated a superior HyperCEST contrast compared to control cells incubated with the biosensor alone. This versatile approach could be easily extended to imaging of other biomolecules (e.g., proteins, lipids) by incorporating an azido bioorthogonal chemical reporter [128].

### 5.4. Xe Biosensor Generation via Single Arm Functionalization of Cryptophanes-223

Although CryA is widely used for the synthesis of different Xe biosensors, inherent setbacks such as generation of complex statistical mixtures and lack of hydrophilic cryptophanes hamper the synthesis as well as the translation of CryA-based biosensors in vivo [163,216]. To surpass this issue, water soluble cryptophanes, e.g., CryA-hexacarboxylic acid have been synthesized and utilized as platforms for introducing further functionalization moieties [161,217,218]. However, due to high symmetry of these water soluble cryptophanes, their purification and preparation at higher quantities (a prerequisite for in vivo applications) is not feasible. Several other approaches have been proposed to attain other versions of water soluble cyptophanes [147,173,219,220]. However, the intended upscaled production and selective functionalization is difficult to achieve. Therefore, other cryptophanes that come with sufficient water solubility, an easily scalable synthesis, and that are amenable for selective functionalization while still providing sufficient Xe binding and exchange are of special interest. In this context, cryptophane-223 can serve as a potential candidate for the generation of intriguing biosensors. Cry-223 is built from two cyclotribenzyl (CTB) units connected together via two ethylenedioxy linkers and one propylenedioxy linker that lead to the formation of hydrophobic cavity slightly larger than that of cryptophane-A. Cry-223 (R^1^ = OCH_3_ (@CTB), R^2^ = H) and its water soluble congener (R^1^ = OCH_2_COOH (@CTB), R^2^ = H) displayed large association constants for Xe and also slow Xe exchange albeit higher than that observed in CryA [146,161,169]. Remarkably, the Cry-223 skeleton offers the possibility for selective functionalization via the propyleneidoxy unit that acts as a single arm without disturbing the main substituents, e.g., methoxy groups substituted at the phenyl rings of the CTB units. For example, tethering a hydroxyl group at the propylenedioxy site does not induce stereogenic center on the latter, even though two CTB units display the same absolute configuration *M* or *P*, respectively [221].

Cry-223 synthesis was attempted via two different approaches in order to achieve reasonably high yields of the final compound. The model compound was a hydroxyl derivative of cryptophane-223 in which the hydroxyl group was tethered at the propylenedioxy linker (single arm) to facilitate further functionalization with moieties of interest during biosensor generation. In the first approach, the model compound was synthesized by connecting the propylenedioxy linker bearing OH groups without protecting groups onto the CTB units followed by subsequent linking of two ethylenedioxy linkers to CTB, respectively. Finally, another CTB unit was connected to the other end of the linkers, thereby completing the formation of Cry-223 (23% yield) with the aid of Sc(OTf)_3_ [222] that enables selective ring closure. In the second approach, a similar synthetic strategy was followed to generate Cry-223 by utilizing protected OH groups at the propylenedioxy linker and formic acid for effecting ring closure at 40–44% yield. Conversion into its hydroxyl analogue, i.e., Cry-223 (R_1_^1–6^ = OH, R_2_ = OH), through either one of the above synthetic approaches resulted in similar yields, e.g., 81–88 %. Other water soluble congeners have been synthesized either by incorporating six polyethylene glycol (PEG) units, e.g., PEG_3_ (85%) or carboxylic acid groups (CH_2_COOH) at the R_1_ position (82%) in which an OH group is present at the propylenedioxy kink (single arm, R_2_ position), respectively. Another water-soluble congener was synthesized at 71% yield by mesylating an alcohol group at the R_2_ position of Cry-223 (R_1_^1–6^ = OH) [221].

In another study, several cryptophane-based hosts of this type were investigated in different solvents. Cry1 (Cry-223 (R_1_^1–6^ = CH_3_, R_2_ = OH)), Cry2 (Cry-223 (R_1_^1–6^ & R_2_ = CH_2_-CO_2_CH_3_)) and Cry3 (Cry-223 (R_1_^1–6^ = CH_2_-CO_2_CH_3_, R_2_ = OH)) [223] indicated Xe binding in (CDCl_2_)_2_ at 64, 82 and 58 ppm, respectively. In the case of Cry2, a large broad signal (8-fold broadening) was observed compared to Cry3 [224]. This was attributed to the presence of the acetate groups at positions R_1_ and R_2_ that induce conformational change at R_2_ and reduce the affinity of Xe to the Cry2 cavity and also depends on the solvent utilized for the measurements. Similarly, water soluble cryptophane derivatives such as Cry4 (Cry-223 (R_1_^1–6^ = CH_2_-CO_2_H, R_2_ = OH)), Cry5 (Cry-223 (R_1_^1–6^ = PEG, R_2_ = OH)) and Cry6 (Cry-223 (R_1_ = R_2_ = OH)) were evaluated using ^129^Xe NMR. The compounds Cry4 and Cry6 revealed bound Xe peaks at 50 and 63 ppm, respectively, but only under basic stabilization conditions. Contrarily, Cry5 (soluble at pH 7) showed a bound Xe peak at 67 ppm [224]. These water soluble cryptophanes (Cry4 and Cry5) displayed slow dynamics on the NMR time scale, indicating their suitability for generation of potential Xe biosensors. Additionally, the Xe exchange was fast enough for detecting these inclusion complexes at micromolar concentration [221].

**Figure 9:**
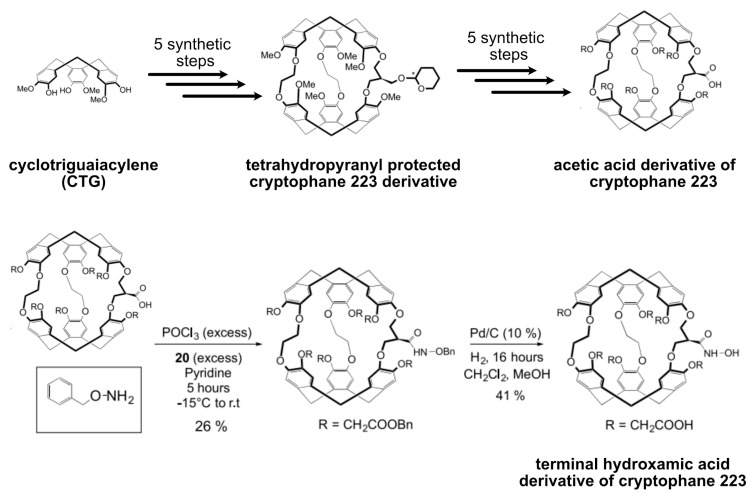
Summary of important synthetic steps involved in the synthesis of novel and functionalizable cryptophane 223 derivatives. The final compound containing terminal hydroxamic acid along with six acetic acid confers water solubility and also enables recognition of specific cationic species (e.g., metal cations) in solution. In this way, while preserving the Xe binding to the host cavity, the functionalization can be carried out more efficiently at the single arm of cryptophane 223 than that of cryptophane 222 (CryA), respectively. Figure adapted from Ref. [225].

Recently, another study was reported on Cry-223 single arm functionalization by Berthault and co-workers [225]. In this approach (summarized in Figure 9), hydroxamic acid was substituted at the single arm of propylenedioxy linker rather than the OH group as in the above study from the same authors. This approach was followed since having a hydroxyl group at R_2_ position along with six CH_2_COOH groups at R_1_ position is not optimal for further functionalization due to the steric hindrance experienced by these functional groups. The hydroxamic derivative of Cry-223 (R_1_^1–6^ = CH_2_COOH, R_2_ = CONHOH, Cry7) was obtained after 11 synthetic steps with a final yield of 41%. However, only the Cry-223 with substituents R_1_^1–6^ = OCH_3_, R_2_ = CH_2_-OH gave X-ray quality crystals and displayed an internal cavity volume of 108 Å^3^ while forming an inclusion complex with an ethanol molecule. Treating Cry7 with Zn^2+^ in tris buffer (pH = 7) gave a 1:1 complexation via the hydroxamic moiety located at position R_2_ (evidenced by ITC results). ^129^Xe NMR of Cry7 at different pH (9.9, 8.2, 3.3) revealed bound Xe peaks at 58.4, 59.4 and 61.3, ppm, respectively. Upon addition of Zn^2+^ or Ni^2+^ (1 and 5 equiv.) to Cry7 (0.2 mM) in tris buffer (pH 7.5) and performing ^129^Xe NMR, only a moderate change in the observed encapsulated Xe signal was observed. Although these new cryptophanes show moderate Xe response to complexation of divalent cations, they still might serve as potential candidates for further functionalization via the single arm to generate targeted Xe biosensors.

### 5.5. Multiplexing Approaches Involving Cryptophanes

Since Xe NMR displays a large chemical shift range of ca. 300 ppm, it can promote the option of having multiple hosts to study multiple analytes via differences in the observed chemical shifts. This approach termed as “multiplexing” has been implemented in the context of different ^129^Xe HyperCEST NMR and MRI applications. One of the approaches to demonstrate multiplexing is by utilizing slightly different Xe hosts for addressing multiple analytes. For example, a CryA-based Xe biosensor including a short linker and a chelating ligand was originally developed for sensing endogenous zinc (Zn^2+^) [218]. The biosensor was synthesized as 50:50 diastereomeric mixtures (1 P and 1 M) in high purity. Direct Xe NMR of above biosensor (260 μM) in PBS buffer at pH 7.4 indicated a bound Xe peak at 65.6 ppm. Upon Zn^2+^ addition the bound Xe peak splits into two (65.75, 67.2 ppm) with equal intensities. This demonstrates the Xe NMR capabilities of distinguishing two different diastereomers of CryA from its racemic mixture effected through structural changes arising due to Zn^2+^ chelation. To confirm this observation, diastereomers (1 P and 1 M) were synthesized by starting from enantiopure cryptophanol-A derivatives, [217] followed by coupling them to the linker bearing the NTA unit. Xe NMR of 1 P and 1 M in the presence of 50 % (*w*/*w*) Zn^2+^ indicated two peaks at 65.75 and 67.2 ppm, respectively. The Xe NMR signal is saturated at 50% (*w*/*w*) Zn^2+^ and this situation denotes the possibility of dimerization in the presence of zinc. Indeed, ^1^H diffusion-weighted NMR showed that a 2:1 complexation is formed between biosensor and Zn^2+^. Under these dimerization conditions, Xe in a cryptophane is unable to detect the configuration of other cryptophane participating in the complexation. A sensitive detection of Zn^2+^ ions (100 nM) was selectively achieved over the weakly binding Ca^2+^ or Mg^2+^ ions using the above Xe biosensor in Xe MRI.

The same authors subsequently utilized this biosensor (1 M), i.e., (+)-*MM*-cryptophane-(L)-NTA, for detecting multiple metal cations simultaneously using ^129^Xe NMR-based multiplexing approach. Here, the host has enabled the detection of unique and well-defined NMR signature through NTA ligand that chelates different metal ions. Exposing the 1 M sensor (970 μM) in 1 M HEPES buffer to successive addition of 5% Zn^2+^, 5% Cd^2+^ and 5% Pb^2+^ ions revealed a specific downfield chemical shift for encapsulated xenon, i.e., 1.5 ppm for Zn^2+^, 0.3 ppm for Cd^2+^ and 4.5 ppm for Pb^2+^, respectively. The Xe NMR-based sensitive detection was also extended to identification of paramagnetic metal ions in solution. For example, Co^2+^ metal ions were tested for chelation by 1 M via NTA. However, no distinct bound Xe peak was observed upon chelation. Conversely, Co^2+^ (150 μM) chelation by another diastereomer (−)-*PP*-cryptophane-(L)-NTA (1P, 300 μM) showed a high field shift of caged Xe by −2.4 ppm, irrespective of the cobalt concentration. The frequency difference observed for the sensor without and with chelation to metal ions of interest remained constant and this insight was helpful to detect metal ions in different mediums and environments, respectively.

Using ^129^Xe HyperCEST MRI, the sensitive detection of Pb^2+^ ions (nM regime) was demonstrated by utilizing 3-fold excess of 1M for chelating the former ions in solution. This method was useful in detecting 1.6 × 10^12^ Pb^2+^ ions in a few seconds while working in nanomolar concentrations. To show the feasibility of this approach in vitro/in vivo, whole human blood was treated with 1 M and Pb^2+^ solutions leading to generation of five peaks in Xe-NMR, i.e., Xe in RBC (1) and in the bulk (2) near 200 ppm, both Xe@1M-Pb^2+^ (3) and Xe@1M (4) near 70 ppm, followed by gaseous xenon (5), respectively. The selective chelation of 1 M to Pb^2+^ was proven through the observation of only one peak related to sensor 1 M under similar experimental conditions. Thus, this chelation based multiplexing approach can be extended to other chelators, e.g., EDTA or DTPA, such that different analytes can be detected simultaneously in solution [226].

Another way to achieve multiplexing is through functionalization of different cryptophanes with different ligands such that various Xe biosensors might display distinct signals. As an example, the avidin-biotin interaction (~10^15^ M^−1^) was exploited to develop different Xe biosensors based on cryptophanes for expediting multiplexing through the attached ligand (biotin). The effect of the cryptophane structure on the chemical shift was shown by measuring two different cryptophanes, namely CryA and CryE in tetrachloroethane, which led to observation of distinct peaks for both cryptophanes (CryA: ca. 70 ppm, CryE: ~30 ppm upfield from CryA peak), respectively. Similarly, conjugation of CryA to different ligands, i.e., in this case biotin via the linker and water-soluble moieties (amino acids) gave rise to the generation of Xe biosensors. The CryA-based biosensor revealed three peaks assigned to Xe@solution (193 ppm), bound Xe (70 ppm) and free CryA (~1 ppm downfield to bound Xe peak), respectively. Introduction of ca. 80 nmol of avidin monomer to the above solution indicated a new third peak at approximately 2.3 ppm downfield to bound Xe peak. Increasing the concentration of avidin in the solution indicated an intensity increase of the third peak in addition to a decrease in the observed intensity of free CryA peak. The specific binding event between avidin and biotin was proven by the appearance of no third peak following the presaturation of the sensor with biotin [16].

Multiplexing can be achieved by using either different types of cryptophane cages in a single solvent [161] or by utilizing two different cryptophanes suspended in immiscible solvents. The two hosts explored in this way were cryptophane-111 (cage 1, 300 μM) dissolved in tetrachloroethane and water soluble cryptophane-A hexacarboxylic acid (cage 2, 300 μM) in water [227]. Xe encapsulation by cage 1 and cage 2 comes with binding constants of 10000 M^−1^ [144] and 6800 M^−1^ [161] at 293 K, respectively. ^129^Xe NMR of above immiscible solutions indicated four peaks at 230 ppm (Xe@tetrachloroethane), 190 ppm (Xe@H_2_O), 60 ppm (Xe@cage 2) and at 38 ppm (Xe@cage 1), respectively. In compliance with the spectroscopic results, Xe MRI at similar experimental conditions showed that the two cryptophanes in their compartments were successfully localized. Overall, the demonstration of the multiplexing approach in any case (in vitro/in vivo) requires rather pronounced differences in observed chemical shift, preferably by utilizing different types of Xe hosts and not just by applying minor residue modifications on a common parent compound. The multiplexing approach can also be implemented by combining a cryptophane-based host with other gas binding structures. This has been demonstrated with PFOB nanodroplets for unspecific cell labelling and will be discussed in “super hosts” section in more detail.

### 5.6. Fluorophore Coupling for Dual Readout

The original Xe biosensor concept relied on the idea that the chemical shift dimension allowed to discriminate between target-bound and free sensors [16]. While this approach does indeed work in neat solution samples, the achieved chemical shifts are usually not sufficient to resolve the two signals in cell suspensions or later in living tissue. This does not impose per se a problem as many fluorophore reporters also simply work as a tag that does not necessarily change their signal upon interaction with the target. In many cases, the reporters rely on a ligand unit with sufficient specific affinity for the target molecule such that unspecific binding in control areas without the target is minimized through simple wash-out. The switch from direct detection to HyperCEST detection was also an important step where the gain in sensitivity was more important than the limited spectral resolution in applications with strong saturation pulses to reveal rather low concentrations of Xe hosts. As MRI does not have cellular resolution, the specific binding of newly emerging biosensors was often visualized by complementary fluorescence microscopy and fluorescence assisted cell sorting (FACS). Integration of a reporter dye into the assembled biosensor (see Figure 5a) thus became a useful additive feature.

A transferrin-targeted sensor introduced by Boutin and coworkers [129] is an example where rhodamine green was coupled to the targeting protein in a similar way as the CryA unit. This allowed verification of cellular uptake by optical microscopy. In cases of smaller targeting units (like short peptides), a dye like Cy3 can be directly integrated into the cryptophane conjugate [228] to achieve the same goal. This strategy was also pursued for a folate sensor [176], generation of bimodal probes for detecting proteins that are genetically incorporated with a tetracysteine tag [229,230] and the sensor for enabling cell surface glycan detection [128,231]. These are examples for validating a targeted cellular uptake/binding of functionalized Xe hosts via fluorophore-based detection. However, it was also shown that CryA conjugates can be internalized without a specific unit that promotes such uptake [232]. Certain CryA-dye conjugates have been demonstrated to embed into phospholipid membranes (presumably due to the hydrophobicity of the cage) but without undergoing a full transition through the membrane bilayer [233]. This points toward a transport mechanism that exists in cells but not in synthetic membrane systems.

Based on this observation, it became clear that a more quantitative analysis than just microscopy would be necessary to quantify unspecific vs. targeted uptake for future biosensor compounds. FACS allows the comparison of uptake in target and control cells if the sensor also carries a fluorescence label. As many dyes are commercially available in a biotinylated form (e.g., a FITC-biotin conjugate) a modular biosensor approach allows to couple both CryA-biotin and FITC-biotin to an avidin-labeled targeting unit. This has been demonstrated for the CD14-targeted sensor [15] and the claudin-4-directed one [194]. Besides such building blocks relying on avidin coupling units, it is also possible to synthesize CryA-dye conjugates with a -COOH handle for further coupling [234]. Importantly, this study showed that the choice of the dye also impacts cellular uptake where TAMRA was enabling better uptake than the previously used fluorescein (presumably due to more pronounced hydrophobicity of TAMRA which enables uptake into the membrane). A dye label can also be used to design a so-called “magnetofluorescent” biosensor: such a sensor yields a change in fluorescence upon target interaction that is also linked to a change in the HyperCEST signal [235].

### 5.7. Cryptophane-Based Theranostic Probes

There is an increasing need for having molecular probes in which multifunctional moieties could be included for achieving both targeted diagnostic detection and therapy measures. Generally, nanosystems (e.g., nanoparticles) have been considered as potential platforms for generating multifunctional probes. However, the size of such nanosystems is considered to be large since the former is mostly taken up by macrophages and cleared by the reticuloendothelial systems (RES), thereby hampering specific uptake by certain cells, e.g., those of the blood brain barrier. To circumvent this issue, small molecules have been proposed for achieving perfusion into tissues. In this context, cryptophane was utilized recently to generate a small molecule-based probe in which multiple functionalities were installed to attain different goals, e.g., targeting, fluorescence, therapy and monitoring thereof. The as-synthesized probe (small molecule multifunctional tool, SMFT) consisted of CryA linked to a hydroxyl derivative of porphyrin via a linker.

Since cancer cells are known to exhibit different pH compared to the normal cells, testing the SMFT probe efficiency at different pH was of special interest. This characteristics of this SMFT probe was evaluated using ^129^Xe NMR and it revealed no observable bound Xe peak for SMFT at 25 μM. However, ^129^Xe HyperCEST NMR-based evaluation revealed a CEST peak at 72 ppm for SMFT between pH 3 and 5. Increasing pH from 5.1 until 9.3 led to the appearance of another peak at 70 ppm and this peak responded linearly to the pH increase. pH > 9 led to the deprotonation of the hydroxyl groups at the porphyrin ring, causing the generation of phenolate groups and an increased intensity of the CEST peak at 70 ppm. Similarly, incubation of the A549 cells with SMFT for 4 h led to the appearance of a CEST peak at 74 ppm (pH 5.1 and 7.4) and comparable ^129^Xe HyperCEST image contrast was observed under similar experimental conditions, respectively. At this juncture, due to the existing in vivo challenges that deter the easy translation of ^129^Xe HyperCEST technique into pre-clinics, the usefulness of this technique for SMFT probe was only demonstrated as a proof-of-principle experiment in vitro. Therefore, it is currently not feasible to investigate and illustrate the impact of small pH changes via SMFT accumulation in tumors under in vivo conditions using ^129^Xe HyperCEST MRI.

The red fluorescence intensity of SMFT decreased between pH 5.1 and 9.3 in a similar fashion comparative to that of the observed ^129^Xe HyperCEST response. The SMFT probe also revealed a strong fluorescence over a broad pH range (3–9.3), thereby fostering biological applications. The confocal microscopy images of A549 cells incubated with SMFT exhibited uniform uptake of the latter in its cytoplasm which was displayed as high red fluorescence intensity. It was reported that the highly acidic microenvironment of cancer cells selectively uptake porphyrin [236]. This selectivity was checked by incubating A549 (lung tumor) and WI-38 (normal lung) cells with SMFT (50 μM) for 4 h. A higher uptake of SMFT by A549 cells was proven through an increased red fluorescence intensity. The in vivo efficiency was evaluated by fluorescence after intravenously injecting the mice bearing A549 tumor with SMFT (1 mM) which led to enhanced uptake of SMFT in tumor (24 h incubation) compared to low accumulation in liver and kidney. The photodynamic therapy (PDT) capability of SMFT (10 μM) revealed singlet oxygen production after 20 min irradiation with 650 nm laser light similar to that of the second-generation photosensitizer 5,10,15,20-tetra (4-sulfanatophenyl)-porphyrin (TPPS4) under similar conditions. Singlet oxygen generation increased linearly with irradiation time and probe concentration and stayed effective between pH 4 and 8. In the cellular context, SMFT displayed an IC_50_ value of 15 μM after irradiating A549 cells with 650 nm laser for 20 min. Under in vivo conditions, mice with A549 tumor received intratumorally injected SMFT (1.15 mM), followed by irradiated with 650 nm laser for 40 min after 2 h with a therapy monitoring period over 20 days. Only in the case of the SMFT+ group a tumor reduction was noticed compared to control groups (e.g., PBS, PBS + irradiation). Although, this study serves as a proof-of-principle for observing the therapy response using a theranostic small molecular probe, still the diagnostic aspect of the reported probe is only observable through the in vivo fluorescence images rather than by the proposed ^129^Xe HyperCEST technique [237,238,239,240].

**Table 1:** Pros and cons of different hosts usage for ^129^Xe molecular sensing applications.

Host	Pros	Cons
Cryptophanes	Display high Xe binding constant and exhibit medium to fast exchange Well studied host for ^129^Xe HyperCEST NMR/MRI applications that offers nM sensitivity Chemical shift tunability by varying the linker length and substituents of CTV caps Bound Xe peak and CEST resonance of cryptophanes can be modulated by switching pH, temperature etc. Functionalizable through different attached handles Several water soluble cryptophanes are available for ^129^Xe biosensing Multiplexing approach demonstrated for different cryptophanes Several cryptophanes-based ^129^Xe biosensors available for different intra- and inter-cellular targets Tethering a fluorophore unit via linker onto the cryptophane-A derivatives indicated delayed toxicity for the latter (80 μM) [234] Incubation of low receptor expressing cell lines with target-specific Xe biosensor indicated low toxicity, e.g., cRGD-CryA and folate-CryA biosensors [127,177]	Cryptophane synthesis is elaborate with multiple steps Very low yields for cryptophane synthesis Undesired conformational change leads to suppression of ^129^Xe binding Commercially unavailable. Cryptophane *syn* and *anti*-isomers either not isolated nor easily synthesizable. Xe binding to cryptophanes is competitively blocked by certain guests, e.g., CHCl_3_ blocks Xe entry into cryptophane A Incubation of mouse fibroblasts (L929) with Cryptophane A monoacid derivative (CryA-ma) showed toxicity at 60 μM [234] Incubation of higher receptors expressing cell lines with Xe biosensors (ca. 100 μM) indicated a higher toxicity for e.g., cRGD-CryA and folate-CryA biosensors [127,177] Clickable, hydrosoluble PEGylated CryA derivative was shown to exhibit 50% toxicity upon its incubation at 60 μM [151] Biocompatibility and toxicity of cryptophanes and their derivatives in vivo are currently unknown
Cucurbit[n]urils (CB[n]s)	Enables fast Xe exchange and gas turnover Can be combined with controlled Xe displacement through competitive guests with very high affinity (e.g., adamantane@CB7 ~10^14^ M^−1^) First experience in in vivo ^129^Xe HyperCEST NMR/MRI applications ‘Odd’ numbered CB[n]s (e.g., CB[5], CB[7]) are water soluble. Acidic solution, counterions (e.g., Na^+^, K^+^) also enhance solubility of CB[n]s CB[n]s Xe response can be regulated by changing the pH, temperature, counterions etc. Easily synthesizable and commercially available Larger CB[n]s might bind Xe in the presence of a co-guest that helps to probe supramolecular architecture CB[n]s forms oligomeric structures aimed for different applications CB[n]s form unconventional host-host inclusion complexes with different hosts e.g., cyclodextrin, calixarenes etc CB[7] exhibits relatively low developmental and organ-specific toxicity in zebrafish models albeit its biomedical application can be exploited at sub-toxic concentrations [240] CB[6] and CB[7] displayed no neurotoxicity at 1 mM concentration (1/3 of maximum tolerated dose for CB[7]) [241] CB[5] and CB[7] showed high cell tolerance at concentrations of up to 1 mM in cell lines originating from kidney, liver or blood tissue using cytotoxicity assays [242] CB[7] was efficiently internalized by macrophages indicating their potential for the intracellular delivery of drugs [242] Biochemical markers testing for vital organs of mice injected with CB[7] (5 g/kg orally, 500 mg/kg peritoneally and 150 mg/kg intravenously) showed excellent biocompatibility for CB[7] [243]	Introduction of functionalizable handle onto CB[n]s structure is not straightforward and faces several synthetic hurdles ‘Even’ numbered CB[n]s are water insoluble or display meager solubility Presence of counterions reduces Xe association constant and its exchange rate Limited Xe chemical shift tunability since co-guest might be needed in certain cases to achieve the desired chemical shift change CB[n]s prefer linear alkylamines, hydrophobic guests over small hydrophobic Xe Multiplexing using CB[n] might be hindered by non-specific competitive binding of different guests. In vitro and in vivo translation are hampered for CB[n]s due to numerous non-specific binding endogenous competitive guests Exposing CB[7] to live zebrafish revealed measurable cardiotoxicity and locomotion and behavioral toxicity at concentrations of ca. 500 μM and negligible developmental and hepatotoxicity at concentrations up to 750 μM [240] Extended exposure to CB[7] (500–750 μM) induced mortality of tested zebrafish [240] CB[6] and CB[7] (each 1 mM) displayed myo- and cardiotoxicity (1/3 of maximum tolerated dose for CB[7]) [241]
Pillar[n]arenes	Easily synthesized with reasonable yields Pillar[5]arene and their counterparts with different counterions (e.g., Na^+^, NH^4−^, SO_4_^2−^) generate contrast via MT saturation effect Water soluble pillar[n]arene derivatives are easily synthesizable The presence of co-guests in pillar[5]arenes allows to tune Xe exchange towards the slow exchange regime Pillar[n]arene-based biosensing is still in its infancy and has lot of potential for tuning the NMR conditions Vesicles formulated using pillar[6]arene for drug delivery indicated low toxicity for normal cells [244,245] Water-soluble pillar[6]arene indicated very low toxicity through its relative cell viability (about 50%) in an extremely high incubation concentration (500 μM) [246]	Xe exchange might be too fast to allow well resolved CEST signal Monofunctionalization of pillar[n]arenes is not straightforward and suffers from synthetic hurdles Pillar[n]arenes are unavailable from commercial sources Co-guest is often needed to observe the desired Xe binding Carboxylated pillar[6]arene at concentrations ≥25 μM in HEK293 cells and ≥250 μM in OVCAR-3 cells significantly inhibited cell proliferation while its pillar[7]arene counterpart inhibited OVCAR-3 cell proliferation ≥100 μM [247] Biocompatibility of pillar[5]arenes both in vitro and in vivo is still lacking In vivo biocompatibility of pillar[6]arene and pillar[7]arene remain unexplored
Metal organic polyhedral (MOP)	Weak Xe binding and relatively slow Xe exchange is observed for MOP Easily synthesized in a single step by combining different organic sub-components under basic conditions Water-soluble MOPs are easily synthesized The organic linker connecting the metal ion centers can be varied in order to generate MOPs with different hydrophobic cavity sizes MOPs also serve as hosts for different macrocycles such that they form cage-in-cage supramolecular structures, e.g., cryptophane-111 inside a MOP The bound Xe peak and CEST response might be altered with varying temperature, pH etc.	MOPs exist in different conformations, e.g., Fe-MOPs exist in 3 conformations, out of which only one is detectable with conventional NMR and techniques. Monofunctionalization of MOP might be difficult to achieve Isolation of different diastereomers of Fe-MOP is not feasible Paramagnetic metal cation present at the MOP might alter the relaxation time T_1_ and depolarize Xe As of now, there are no reports available on in vitro and in vivo toxicity of MOP
Modular hosts (micelles & liposomes)	Easy production of micelles and liposomes with controllable size and layers Encapsulation of hundreds of hosts in a liposome is possible e.g., with cryptophanes in liposomes Offers possibilities to indirectly prepare targeted hosts by functionalizing the outer layer of liposomes with desired target moieties Hosts such as cryptophanes can be tethered to the phospholipid or to micelle surfactant molecule prior to producing the micelles and liposomes Dual modality biosensors can be established using cryptophanes and dyes for ^129^Xe HyperCEST NMR and optical imaging Multiplexing approach might be feasible upon introduction of different hosts inside the liposomes Stable over months with fabricated size and encapsulated cargo, e.g., Xe hosts, drug etc. Modular hosts serve as ideal models for studying membrane-embedded Xe and its host interactions and membrane fluidity Generally, micelles and liposomes are considered to be non-toxic both in vitro and in vivo [248,249] These modular hosts are mostly utilized as drug carriers thereby leading to successful drug delivery in vivo.	Unlabeled modular hosts exhibit only medium to fast Xe exchange due to rather loose transient binding Tethering Xe hosts, e.g., cryptophanes, onto the phospholipids, micelles arm might not be straightforward. Excessive encapsulation or loading with Xe hosts inside phospholipid carriers might disrupt the liposomal layers. Appropriate molar ratios of phospholipids, hosts and dyes can be achieved only after several trial and error attempts Starting materials for generating the liposomes are often expensive
Biogenic scaffolds (viral capsids, bacteriophages)	Serve as platforms for covalently tethering high payloads of Xe hosts e.g., cryptophanes. Enables detection at nanomolar concentrations of biogenic scaffolds. Different accessible amino acid residues available for activation using different chemical agents for coupling with hosts, dyes, targeting moieties etc. Stable to harsh reaction conditions, pH and temperature changes Xe exchange is mostly restricted in and out of the anchored host but not around/through the scaffold surface Capsids allow decoration of both inner and outer membranes with different molecules of interest Bacteriophages present large number of p8 proteins (>1000) for chemical modification with different molecules Bacteriophages can be easily engineered to express Scfv fragments of any antibody that might be utilized for targeting a cell receptor, e.g., the EFG receptor. Generally, viral capsids and bacteriophages (M13, fd) are considered to be non-toxic for both in vitro and in vivo applications Evaluation of commercially available bacteriophages cocktail revealed no signs of in vivo toxicity [250] No evidence of M13 bacteriophage toxicity was reported for normal and cancerous colorectal cell lines [251] M13 bacteriophages were also found to be safe in vivo, e.g., no change of liver and renal functions upon M13 administration [251]	Engineering of biogenic scaffolds is cumbersome and time consuming Higher concentrations of biogenic scaffolds, e.g., bacteriophages, are insoluble in water and biological solutions Higher modifications of the scaffolds with different molecules, e.g., host, linker, dye, etc. might display low efficiency by the modified scaffold Long term storage of modified scaffolds in biological buffers at 4 °C leads to degeneration of the probe Only cryptophanes have been tethered onto such scaffolds; conjugation of other Xe hosts remains largely unexplored Bacteriophages are known to be immunogenic in nature [252]
Gas binding proteins	can be engineered to enhance Xe binding and for regulating its fast exchange into surrounding medium from different binding pockets Show a dedicated signal for bound Xe with direct detection and in ^129^Xe HyperCEST NMR at μM concentrations ^129^Xe serves as a surrogate (e.g., for oxygen) for probing the structure and dynamics of different gas binding proteins Multiplexing approach possible by using two different gas binding proteins e.g., bla, and maltose binding protein Point mutagenesis of above proteins at specific residues leads to better CEST response at lower concentrations Responsive biosensors for certain protein targets, e.g., maltose binding protein changes Xe HyperCEST response upon addition of maltose Gas binding proteins are shown to be non-toxic, e.g., TEM-1β-lactamase [253]	Mutagenesis and genetic engineering of gas binding proteins is time consuming and requires many preparative steps Expression of such proteins in *E. coli* with handles necessary for further functionalization might not be straightforward Non-specific binding of Xe to pockets/cavities on the surface of the proteins might interfere with the desired bound Xe signals To observe responsiveness from gas binding protein biosensors, supplying a higher concentration of the substrate might be inevitable Under extreme conditions, usage of Xe might hinder or alter the protein structure or functions In vivo toxicity of gas binding proteins seems to be no issue in ultrasound imaging studies
Superhosts (Nanoemulsions, gas vesicles)	Can encapsulate and exchange a huge payload of Xe atoms Allow detection of Xe at picomolar sensitivity Can be generated either synthetically or through genetic engineering Genetically engineered gas vesicles (GVs) are reported to host and exchange more Xe atoms compared to synthetic nanoemulsions e.g., PFOB nanoemulsions Multiplexing ^129^Xe HyperCEST approach has been demonstrated for GVs possessing different lengths and shapes generated from diverse bacterial species GVs can be combined with a targeting moiety, e.g., EFGR antibodies on their surface GVs enable stable HyperCEST contrast due to physical partitioning instead of chemical affinity Nanoemulsions can be functionalized indirectly via the protective phospholipid layer similar to liposomes PFOB emulsions are reported to be non-toxic in vivo [254,255,256] No acute or adverse toxicity was noticed for mice injected with gas vesicles (GVs) [257]	Nanoemulsions tend to increase in droplet size over time, leading to unstable emulsions and change in observed CEST intensity GVs collapse at certain overpressure conditions, therefore care should be taken during handling Circulating life time of GVs in vivo might require repeated administration and impact thereof must be accounted for Stability of GVs for longer time is also of a concern

### 5.8. Xe Binding to Other Synthetic Hosts: Different Exchange Kinetics

**Figure 10:**
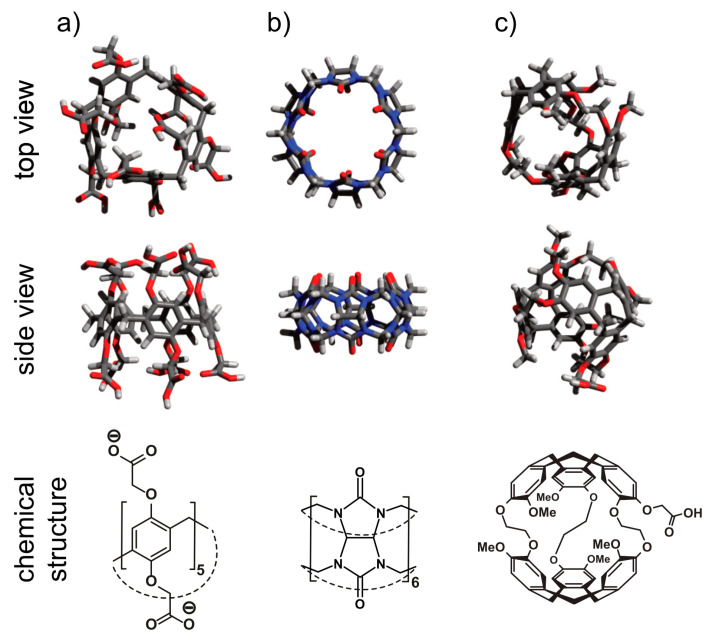
Comparison of Xe hosts: (**a**) a water-soluble pillar[5]arene; (**b**) cucurbit[6]uril (CB6); (**c**) cryptophane-A-monoacid (CryA-ma). Reproduced with permission by Wiley & Sons from ChemPhysChem 20, 246–251 (2019).

Optimizing the affinity of Xe to cryptophane hosts became less important for the Xe biosensor design with the advent of the HyperCEST approach. Instead, it was recognized that moderately improved exchange conditions would be beneficial. Although cryptophanes serve as the gold standard for achieving highly sensitive ^129^Xe NMR detection, the former’s synthesis is not easy due to multiple steps leading to very low yields. This prompted several research groups in the Xe community to search for alternative hosts that can be easily synthesized or that are commercially available. This also led to the investigation of synthetic hosts with easier access to the binding cavity (see Figure 10).

#### 5.8.1. Cucurbit[n]urils-CB[n]

Cucurbit[n]urils (CB[n]) are a class of macrocycles whose trivial name was derived from the pumpkin’s botanical name (*Curcurbitaceae*) due to the host’s structural resemblance to pumpkins. These macrocyclic structures comprise a rigid hydrophobic interior cavity which is accessible to different guests via the two opposing quasi-planar portals that are decorated with carbonyl groups. Although the first CB[n] synthesis was reported quite early, the structural elucidation of CB[n] was carried out by X-ray diffraction only a few decades ago [256]. The classical way of synthesizing CB[n] is a straightforward reaction in which glycoluril and formaldehyde or paraformaldehyde were reacted in acidic (e.g., H_2_SO_4_ or HCl) and refluxing conditions to yield a mixture of different CB[n], albeit CB[6] being the predominantly isolated species [257]. The glycoluril (tetrahydroimidazo[4,5-*d*]imidazole-2,5-dione) unit was synthesized by treating urea with diketones (e.g., glyoxal) via the condensation under acidic conditions [258,259,260,261,262,263]. Since the condensation reaction is exothermic, the quality of the glycoluril obtained depends on the temperature rise observed during the reaction. Glycoluril has been often utilized as the precursor for the generation of unsubstituted CB[n]s. Both C- or *endo*-shaped and S- or *exo*-shaped methylene-bridged glycoluril dimers were recognized as the fundamental building blocks for generating the CB[n]s and its related derivatives [264,265,266].

Different members of the unsubstituted cucurbit[n]urils (n = 5,6,7,8,10,14 etc.) are generally obtained by employing higher equivalents of formaldehyde to glycoluril units (2:1) under acidic reaction conditions [261]. The formation of CB[n] starts with the dimer generation, followed by oligomer ribbon formation and finally proceeding to ring closure [263,267,268,269]. This was clearly supported by the isolation of the oligomers [261] in accordance with a step-growth cyclo-oligomerization process [270,271] that operates during the formation of CB[n]s. For example, Kim and co-workers synthesized a mixture of CB[n]s depending on their reaction conditions, i.e., glycoluril treated with formaldehyde in 9 M H_2_SO_4_ at ~75 °C for 24 h, followed by 12 h treatment at 100 °C. Using this classical synthetic approach, only CB[5] (10%), CB[6] (60%), CB[7] (20%), and CB[8] (10%) were isolated from the reaction mixture [272,273]. The CB[n] mixture obtained from classical synthesis could be categorized into two parts [274]—a water-insoluble fraction comprising mainly even “*n*” such as CB[6], *i*CB[6] (“inverted” CB[6] [258]), CB[8] and CB[5]@CB[10], and a water soluble fraction comprising CB[n] types with odd “*n*” like CB[5], CB[7] and *i*CB[7], respectively. The *i*CB[6] and *i*CB[7] are formed as kinetic intermediates during the synthesis of CB[n]s in HCl (85 °C) with “ring contraction”, followed by formation of more stable smaller rings, e.g., CB[5] and CB[6], respectively [275]. It was found that both the solvent and the reaction temperature have a pronounced effect on the achievable relative percentages of CB[n]s [272].

The unsubstituted CB[n]s with an even “*n*” such as CB[6], CB[8], and CB[10] display poor solubility in common solvents and also exhibit chemical inertness that limits their further applications. The aforementioned issues with ‘even’ unsubstituted CB[n]s might be addressed by the generation of substituted CB[n]s that improve solubility. Furthermore, it might provide a handle that is amenable to functionalization [276]. This led to the generation of substituted CB[n]s such as cyclohexane-substituted (CyH)_n_CB[n]s (CyH = cyclohexano; n = 5–8 [277]), Me_12_CB[6] (Me = methyl [278,279]), (CyP)_n_CB[n] (CyP = cyclopentano [280]), Ph_2_CB[6] (Ph = phenyl [281]), HMeCB[6] (H = hexa [282]), TMeCB[6] (T = tetra [283]), (CyH)_2_CB[6] [284] and (Me_2_CyP)_n_CB[6] [278], respectively. Among the different substituted CB[n]s, the cyclohexane-based derivatization of the CB[n] (n = 5–8, CyH_n_CB[n]s) rendered them more soluble in both water and certain organic solvents. For example, 1,4-DiCyHCB[6] exhibited good solubility in water while displaying similar host-guest properties as that of unsubstituted CB[6] albeit the latter is almost insoluble in water [285].

As CB[n]s possess carbonyl groups on either side of the portals, they might be prone to binding to different metal cations, e.g., monovalent alkaline cations. These CB[n]s are known to coordinate to metal cations thus fostering the formation of supramolecular frameworks [276,286,287]. Such interactions are driven mainly through coordination, ion-dipole, or through hydrogen bonding. For instance, two Na^+^ ions coordinate to CB[6] on each side of the latter’s carbonyl portals that act as a lid on a barrel [288,289,290]. These “Na^+^-lidded” CB[6] was shown to encapsulate small guests such as THF and benzene in a reversible fashion under the influence of the pH change. This approach was extended to Cs^+^ ions since its ionic radius (1.81–2.02 Å) suggests that it might fit better at the CB[6] portals. Slow diffusion of ethanol into CB[6] suspended in 0.2 M CsCl solution resulted in formation of Cs^+^-coordinated CB[6] crystals. Contrary to Na^+^ ions, under higher concentration only one Cs^+^ ion was coordinated to one of the CB[6] portal via four carbonyl groups, thus forming a so-called “metal-ion-bottomed” molecular bowl. Evaluation of Cs^+^-coordinated CB[6] (2.6 × 10^−2^ M) with regard to its ability to accommodate small guests such as THF was tested in 0.4 M CsCl solution. ^1^H NMR analysis of the THF indicated two signals one for free and another one for encapsulated THF. The formation constant for such an inclusion complex was found to be (1.1 ± 0.1) × 10^3^ M^−1^ at room temperature which is larger than sodium-complexed CB[6] ((5.1 ± 0.5) × 10^2^ M^−1^ [288]. Similar to Na^+^-complexed CB[6], the Cs^+^ counterparts also release the encapsulated guest in a reversible way upon a stimulus induced by a change in pH (acidic/basic cycle) [291].

CB[6] is known to encapsulate small molecules and monoatomic guests such as Xe in water, acidic or saline solution, thus producing a bound Xe signal at (122 ± 0.5) ppm compared to free Xe signal at (190 ± 0.5) ppm, respectively. Addition of excess salt or acidic pH is necessary to solubilize higher concentrations of CB[6] in solution. However, such conditions might deter the absolute determination of the binding constant. Therefore, Kim and co-workers employed a water soluble and substituted CB[6] for determining the Xe binding constant. In this context, water soluble Cy_6_CB[6] at higher concentration showed a Xe binding constant of (1.3 ± 0.1) × 10^3^ M^−1^ and a bound Xe resonance at 93 ppm. Additionally, Xe@Cy_6_CB[6] displayed a *T*_1_ of ~40 s that is sufficient for performing Xe NMR where the -CH_2_- protons at the portals might foster the Xe relaxation in this derivatized CB[6] [292]. CB[6] and CB[7] without any further functionalization have been used in so-called displacement studies with ^129^Xe NMR that are described in more detail in Section 6.2.

To exemplify the use of unsubstituted CB[6] that has limited water solubility for different applications, a rotaxane approach was followed such that the CB[6] can participate as the rotaxane component and could promote the ^129^Xe NMR-based molecular sensing of an analyte of interest. The “rotaxane” acronym derived from Latin words for “wheel” and “axle” describes a compound that comprises a linear species (also called rod like part or guest) and a cyclic species (also known as beadlike part, ring, or host) which are bound together in a threaded structure via non-covalent forces. These components are forced to stay together due to the presence of so-called ‘stoppers’—sterically bulky groups that are large enough to not pass through the ring component [293]. Rotaxanes can be categorized into two groups, namely pseudo- and semi-rotaxanes, respectively. Rotaxanes without bulky stoppers at both ends of the axle are ‘pseudorotaxanes’, meaning that it is a supramolecular complex rather than a compound. Conversely, a pseudorotaxane tethered to only one stopper is termed “semirotaxane”. As a type of interlocked molecules, rotaxanes are used as a platform for constructing functional artificial nanomachines [294].

The rotaxane approach was successfully utilized to generate a chemically activated CB[6]-rotaxane platform for ^129^Xe NMR-based applications [295]. In this strategy, the ‘dumbbell’ stopper component of the rotaxane blocked ^129^Xe from accessing the CB[6] cavity in an uncleaved state. Cleavage of the unit then enables freeing of the CB[6] cavity for ^129^Xe binding. The rotaxane stoppers generated in this study were pyrene-functionalized 2-azidoethylamine (PyAA^+^) and an adamantyl-ester functionalized propargylamine (AdPA^+^). They were combined together via CB[6]-catalyzed azide-alkyne 1,3-dipolar cycloaddition. Cyclodextrin was utilized as caps to improve the solubility of the end groups (i.e., stoppers) of the rotaxane [296,297]. Testing the fully assembled rotaxane (100 µM) using ^129^Xe NMR indicated no Xe@CB[6] signal at relatively high concentration. This indicates that CB[6] is completely locked in the rotaxane complex. The CB[6]-rotaxane was activated by subjecting it to excess base-catalysed ester hydrolysis (e.g., LiOH for 8 h). This led to the generation of semi-rotaxanes consisting of a single bulky rotaxane stopper and a terminal carboxylate on the axle component of the rotaxane with these semi-rotaxanes having the association and Xe exchange kinetics necessary for producing a HyperCEST response. This will be beneficial in investigating complex biological systems without any background CB[6] release due to it being mechanically locked into a rotaxane. It can then be released at a region of interest by an endogenous stimulus.

Similarly, the rotaxane-based modular approach was utilized to generate Xe sensors for selective and sensitive detection of protease activity (e.g., MMP-2) using ^129^Xe HyperCEST NMR. The stopper (PyAA^+^) was connected with (5,6)-carboxytetramethylrhodamine (TAMRA) as a stopper via an axle containing the PLG-LAG recognition sequence for matrix metalloprotease-2 (MMP-2). As described above, the CB[6]-catalyzed azide-alkyne 1,3-dipolar cycloaddition introduced CB[6] onto the axle while combining different components of the rotaxane together. No significant CEST effect was found for the intact rotaxane, thus indicating that the two bulky stoppers at the end cause the CB[6] to remain in place without being released from the rotaxane axle. The rotaxane (5 µM) was subjected to cleavage for 24 h by the enzyme MMP-2 (5 nM), leading to CB[6] release from the rotaxane axle and resulting in a sensitive detection (15% CEST effect) of MMP-2 using ^129^Xe HyperCEST [298].

As another example, the CB[6]-rotaxane platform was utilized for investigating extracellular H_2_O_2_ at low physiological levels (0.5–50 µM) associated with different disease states [299,300]. The as-generated rotaxane consisted of a *p*-xylenediamine moiety that displayed the lowest reported *K*_a_ value for CB[6] (5.5 × 10^2^ M^−1^) in order to allow easy synthesis in addition to an H_2_O_2_-cleavable cap of an aryl boronic acid group on the rotaxane. The other end of the non-responsive rotaxane was tethered with a fluorophore to validate the H_2_O_2_ activity via fluorescence or with a maleimide such that it can be further conjugated to biomolecules prior to H_2_O_2_ detection via ^129^Xe HyperCEST. The Xe@CB[6] response was observed after the CB[6] release from the rotaxane due to the cleavage (1 h) induced by addition of 50 µM H_2_O_2_ to 25 µM rotaxane resulting in an apparent maximum saturation of about 25%. Additionally, mass spectrometry data indicated that the H_2_O_2_ usage did not alter the molecular structure of the CB[6] or the cleaved axle.

To show the feasibility of coupling a maleimide on rotaxanes to a biomolecule, the rotaxane was conjugated to cysteine residues present on the vascular cell adhesion molecule (VCAM)-1 binding peptide and onto a tobacco mosaic virus (TMV) protein-based nanoparticle. This protein assembly could be advantageous in drug delivery and imaging applications [301,302]. The VCAM-1 peptide was completely modified while the TMV was intentionally modified to 30% resulting in 10 rotaxanes per TMV disk. HEK293T cells were then exposed to tumor necrosis factor alpha (TNF) as a signaling protein involved in immune cell regulation that causes an increase in production of cellular H_2_O_2_ [303,304]. The cells were exposed to 40 µg of TNF (TNF+) or remained untreated (TNF-) and incubated for 6 h at 37 °C. Next, the supernatant was treated with TMV-rotaxane conjugate (final concentration of 1 µM) and the reaction was followed by ^129^Xe HyperCEST over several hours. No significant Xe HyperCEST response was observed for untreated cells (TNF-) even after 8 h, while a stable response was visible for TNF+ sample after 2 h and persisted until 24 h albeit a significantly broadened signal. This demonstrated that a low micromolar level of H_2_O_2_ expressed in cells could be successfully estimated under biologically relevant concentrations [305,306].

Recently, a monosubstituted, i.e., directly functionalized CB[n] was shown to detect a specific protein target at ease using ^129^Xe HyperCEST. In this regard, a directly biotinylated CB[7] (btCB[7]) was utilized for Xe NMR investigations. Earlier, btCB[7] was synthesized as a carrier system for delivering anti-cancer drugs to murine lymphocytic leukemia cancer cells expressing higher levels of biotin receptors [307]. ^129^Xe HyperCEST of unmodified CB[7] indicated a response similar to that of previous reports [124]. Conversely, the Xe@btCB[7] response appeared at δ = −68 ppm similar to unmodified CB[7] albeit significantly weaker than the latter. Due to the large diameter of CB[7] (7.3 Å) a faster Xe exchange than CB[6] and a broader Xe@CB[7] resonance was anticipated. The broadening for Xe@aq (0 ppm) was larger than expected in comparison to the width and intensity of the bound Xe peak. The peak at −68 ppm was hypothesized to arise from a known, low population (~1 %) stereoisomer *i*CB[7] in which a single glycoluril unit is inverted [258]. The *i*CB[7] is also known to possess reduced internal cavity size (~5.5 Å diameter), resulting in preference for smaller guests. The CB[7]-bound Xe resonance is too broad for direct detection, however, its presence is manifested in the broadened Xe@aq peak [258]. Interestingly, the Xe@*i*btCB[7] is significantly weaker than the Xe@*i*CB[7] which could possibly be due to less *i*btCB[7] formed during its synthesis compared to *i*CB[7] generated from unmodified CB[7] synthesis. Additionally, the observation might be due to an exclusion complex formation between *i*btCB[7] and its own biotin tail or to that of another *i*btCB[7]. However, MD simulations indicated that the biotinylated tail does not interfere with the CB[7] portals, thus fostering Xe access to the cavity. It could be as well due to dimerization between two *i*btCB[7] molecules in solution. Indeed, the dimer formation was supported through MALDI analysis and MD simulation results. However, avidin binding to biotin is strong enough to disrupt the btCB[7] dimerization [235], thereby producing meaningful Xe NMR responses. Addition of avidin to btCB[7] (50 µM) revealed four distinct signals, namely at −68 ppm (Xe@*i*btCB[7]), 0 ppm (Xe@aq) and new signals at δ = −40 ppm and 100 ppm, respectively. The resonance at 100 ppm was assigned to interaction of Xe atoms with avidin. The substantial response at δ = −40 ppm was assigned to btCB[7] bound to avidin (Xe@btCB[7]-avidin) which was supported by observing no signal in the case of unmodified CB[7] in the presence of avidin. Similarly, no signal occurred for btCB[7] in the presence of avidin that was pre-saturated with biotin. Additionally, the imaging potential of such interactions between btCB[7] and avidin was estimated to generate ~50 % CEST difference, which is sufficient to provide a significant imaging contrast. Thus, achieving close proximity of these hosts to protein targets infer functionalized CB[n]-based biosensors as very attractive candidates for imaging applications [308].

#### 5.8.2. Pillar[n]arenes

Pillar[n]arenes are another family of macrocycles which are pillar-shaped or cylindrical cyclic hosts covered with aromatic walls [309,310,311,312]. Unlike calix[n]arenes, the repeating units in pillar[n]arenes are connected via methylene bridges at 2- and 5-positions (i.e., *para*-position), respectively. This leads to the generation of pockets that are open at both ends. Pillar[n]arenes are named after the inspiration gained from the highly symmetrical pillars that constitute the Parthenon in Athens [309]. They are constructed from electron-rich 1,4-dialkoxy-benzenes. These manifest the cavity with an affinity for electron-deficient guests and also enhance the π-electron density in the cavity, respectively. Pillar[n]arenes display a preference to bind cationic guests such as pyridinium, viologen and ammonium moieties. They also bind neutral linear molecules with electron-withdrawing groups (see, e.g., the affinity of alkylated pillar[5]arenes for neutral guests).

Generally, pillar[5]arenes are synthesized from the reaction of 1,4-dialkoxybenzene with paraformaldehyde in the presence of an appropriate acid. Interestingly, the solvents used for the reaction determine the number of the repeating units generated during the synthesis of different pillar[n]arenes. For example, pillar[5]arenes are prepared in high yields in 1,2-dichloroethane while larger pillar[n]arene homologues (e.g., n = 6,7,9,10) and linear oligomers are generated in chloroform or chlorocyclohexane utilized as solvent. Importantly, pillar[n]arenes can be easily functionalized [312] through their alkoxy substituents on both rims that can be converted into phenol, bromide, azide and alkyne moieties. Additionally, the physical properties of pillar[n]arenes such as solubility, conformational aspects, and guest affinity are governed by the substituents available at the rims. Pillar[n]arenes with alkyl and phenolic substituents are only soluble in organic solvents and they form host-guest complexation only in organic phase. Conversely, appending hydrophilic moieties like cationic or anionic groups onto both rims of pillar[n]arenes render them water soluble. A water soluble pillar[5]arene was prepared by modifying its rims with 10 carboxylate anions via the hydrolysis of the ethoxycarbonylmethoxy-substituted pillar[5]arene under basic conditions [313]. This water soluble pillar[5]arene formed a 1:1 host-guest inclusion complexes with cationic viologen salt, namely paraquat, and the determined association constant of the complex was (8 ± 2) × 10^4^ M^−1^. This is almost 70-fold higher than for complexes formed between paraquat and a pillar[5]arene substituted with 10 phenolic moieties in MeOH. Water soluble pillar[5]arenes decorated with carboxylate groups also displayed highly selective binding (*K* = ca. 10^3^ M^−1^) towards amino acids such as L-arginine, L-lysine and L-histidine, respectively. The water soluble pillar[n]arenes with large cavities did form host-guest complexes with larger guests such as naphthalene diimide and 1,10-phenanthrolinediium cations. The cationic pillar[5]arene containing 10 trimethyl ammonium groups on the upper and lower rims act as a good anion receptor and indicated a high affinity for sodium alkyl sulfonates in water, e.g., 1:1 complexation of cationic pillar[5]arene with 1-octanesulfonate in water (*K* = (1.3 ± 0.9) × 10^4^ M^−1^) has been reported [314].

Water soluble pillar[5]arenes as hosts for small neutral guests (e.g., hexane, xenon etc.) have been studied by using ^1^H diffusion and ^129^Xe NMR, respectively. For the investigation, water soluble pillar[5]arenes were developed by appending carboxylate groups at both rims [313]. ^1^H NMR diffusion measurements in water indicated that hexane was encapsulated by carboxylate-substituted pillar[5]arene. The peak appearing upfield was assigned to the hexane threaded inside the pillar[5]arene cavity while the unencapsulated hexane peak occurred downfield with much less intensity. Based on the diffusion results it was proven that a pseudorotaxane was formed as a 1:1 inclusion complex. Furthermore, the capability to host Xe in the absence and in the presence of hexane was investigated using ^129^Xe NMR. Xe was encapsulated by the host in the presence of a hexane “thread” as it was revealed by an upfield shift of more than 75 ppm compared to the external reference solution (i.e., Xe solubilized in CDCl_3_). As the pillar[5]arene cavity was more lipophilic due to hexane encapsulation, a downfield shift for Xe chemical shift was anticipated and also it led to a chemical shift by more than 10 ppm downfield for Xe compared to Xe@pillar[5]arene, respectively. This study demonstrated the feasibility of utilizing Xe as the NMR reporter for obtaining information on the additional encapsulated guest inside a water soluble host such as pillar[5]arene derivatives [315].

In a subsequent study, our lab tested Xe binding to several water soluble pillar[5]arenes differing only by the counterions (e.g., Na^+^, NH_4_^+^, Br^−^) mainly through magnetization transfer (MT) experiments of loosely bound Xe [125]. This screening led to the conclusion that Xe is displaying fast exchange that is tunable by counterions impacting the exchange kinetics. ^129^Xe NMR of the water soluble pillar[5]arene with Na^+^ counterions (340 µM) exhibited no resolved signal for transiently bound Xe but only a sharp resonance for the dissolved Xe. Conversely, the same compound with NH_4_^+^ counterions (100 mM) in D_2_O indicated a bound Xe signal significantly larger than the free Xe resonance [315]. This was attributed to the large host excess in the latter study compared to conditions where dissolved Xe and host molecules are present at comparable (µM) concentrations. Pillar[5]arenes are actually known to exhibit a high affinity for larger guests, e.g., propane over methane [316]. Therefore, monoatomic Xe is anticipated to form rather labile/high exchange rate complexes with pillar[5]arene containing Na^+^ counterions. Unlike CB[6], only the neutral guests such as alkyl chains and alkanes bind to pillar[5]arenes containing Na^+^ or NH_4_^+^ counterions in aqueous media without displacing the co-guest Xe [315].

The magnetization transfer (MT) for detecting the presence of a host works similar to the CEST effect but differs by observing a broad saturation response manifested around the signal of free Xe rather than well resolved saturation response. Pillar[5]arenes with sodium counterions revealed a pronounced MT effect at 5 µM, i.e., a broad saturation response with FWHM of ca. 700 Hz at 295 K around the chemical shift of dissolved Xe. For comparison, the analogue response from solutions containing the parent pillar[5]arene, pillar[5]arenes with NH_4_^+^ counterions, or a reference sample without host had a width of only ca. 100 Hz FWHM. The underlying difference in MT effect observed between parent compound and counterion-containing pillar[5]arenes might arise due to partial blocking of its portals by different counterions. Similar to CB[6], the presence of counterions might cause a modulation of the Xe exchange rate at the pillar[5]arenes. A hypothetical aggregation of pillar[5]arenes for explaining restricted Xe access could be excluded as the MT effect prevailed even at very dilute conditions (200 nM) in addition to no significant change in their observed hydrodynamic sizes. ^129^Xe MRI scans of the pillar[5]arene with Na^+^ counterions demonstrated a superior MT performance compared to a HyperCEST response from CryA: a reference sample with CryA-ma at 5 µM yielded a CEST signal of 29% at 10 µT saturation field strength whereas the MT image (ca, −5.61 ppm offset from free Xe) showed a maximum signal change of 47%. Although the presence of CryA-ma also causes an MT response, this was only a moderate effect due to the existence of an apparent slower Xe exchange. Thus, MT detection using such pillar[5]arenes could be a promising alternative to CB6 where limitations arise due to non-specific interactions occurring with other binding partners.

#### 5.8.3. Metal-Organic Polyhedra—MOPs

Metal organic polyhedra (MOP) are a class of discrete supramolecular metal complexes which self-assemble from metal cations and organic sub-components in solution. Such metal complexes form hydrophobic cavities of varying sizes and promote hydrophobic guest encapsulation under aqueous and organic conditions [317]. The water-soluble tetrahedric (*T*_d_) Fe-MOP version ([Fe_4_L_6_]^4−^ [(CH_3_)_4_N]^4+^) can be self-assembled from 4,4′-diaminobiphenyl-2,2′-disulfonic acid, 2-formylpyridine sub-components and Fe^2+^ under alkaline condition. The four Fe^2+^ centers are located at the vertices and ligands bridge them along the six edges of the tetrahedra. The sulfonate groups situated at the exterior promote high water solubility while the aromatic rings form an interior hydrophobic cavity (141 Å^3^) that is essential for guest encapsulation. This Fe-MOP was shown to encapsulate different types of guests in water, e.g., P_4_, SF_6_, cyclohexane, cyclopentane, THF, benzene [317,318,319,320]. After preliminary screening of several gaseous guests for Fe-MOPs, it was initially found that Fe-MOP showed no binding affinity for different gases (including Xe, Ar, N_2_, O_2_, C_2_H_4_, CO_2_, N_2_O). However, recently it was shown for the first time that indeed a Xe encapsulation by Fe-MOP in H_2_O occurs. This was demonstrated by ^129^Xe NMR and computational modeling.

The late discovery of Xe encapsulation by Fe-MOP may be seen in the context of different Fe-MOP diastereomers. In theory, the Fe(II) stereocenters at Fe-MOP generate three different diastereomers, i.e., homochiral *T* (ΔΔΔΔ/ΛΛΛΛ), heterochiral *C*_3_ (ΔΔΔΛ/ΛΛΛΔ) and achiral *S*_4_ (ΔΔΛΛ), respectively. The Fe-MOP encapsulates differently sized guests by undergoing structural alterations through one of the plausible mechanisms, namely non-dissociative (constrictive binding) and partial dissociative mechanisms [321]. It is proposed that this Fe-MOP encapsulates Xe as *T* diastereomer via the constrictive binding mechanism. However, the Xe encapsulation process is further influenced by the solvent, the orientation of the guest with respect to the host or additional guests, guest reactivity, and temperature. Using Fe-MOP (8.9 mM) for encapsulating Xe in H_2_O (Xe solubility = 20 mM in H_2_O at 298 K and 4.9 bar) implies only 24% occupancy of the total supramolecular cages and revealed a Xe binding constant of 16 M^−1^. Such low affinity is most likely due to the less optimal ratio of the volume of the Xe atom to the cavity volume enclosed by the Fe-MOP (here, ca. 30% for Xe). Interestingly, the Xe@Fe-MOP resonated at a higher chemical shift (16.6 ppm downfield) than free Xe in D_2_O. The 2D Xe EXSY spectrum in D_2_O and least-square fits of a two-site exchange model revealed a Xe exchange rate of 10 Hz. This also proves that the downfield signal with slow exchange is arising from Xe@Fe-MOP whereas Xe interaction with a sub-component or exterior of the cage will involve a faster exchange rate. Further, ^1^H NMR-based investigation showed that this Fe-MOP undergoes small structural alteration via constrictive binding for encapsulating Xe compared to binding of the larger guest cyclohexane. Such structural alterations (dynamics) of Fe-MOP are accelerated at higher temperatures for encapsulating larger guests in a non-dissociate way. By optimizing the cage dimensions, new Xe molecular reporters can be developed with a provision for installing targeting moieties by synthesizing a “non-self-assembled” MOP [322].

Recently, an iron (II)-based MOP was utilized to encapsulate another small molecule-based macrocycle in order to generate host-in-host complexes as a new class of enantiopure Russian dolls for improvising the guest encapsulation properties of the inner macrocycle. Interestingly, such host-in-host encapsulation was helpful for gaining better stereochemical control over the inner host. This also enabled to drive the guest binding of the inner macrocycle, namely encapsulation of a cation in a highly positively charged environment of the outer host system [323,324]. Additionally, the guest nuclei at the inner host were able to sense the chirality of the outermost host in spite of it being in certain stereochemistry configuration [153,325]. The outer host was a coordination-driven self-assembled polyhedra formed by combining the subcomponenets triazatruxene, bis(trifluoromethanesulfonyl)imide, and 2-formylpyridine in the presence of Fe(II) in acetonitrile. This self-assembly led to the generation of Fe^II^_4_L_4_ MOP in which one triazatruxene cap aligns along each face of the tetrahedra (*T*_d_). The inner host, cryptophane-111 (CRY), was built by combining two cyclotribenzylene (CTB) units [326]. This unit was reported to encapsulate small guests, e.g., CH_4_, Xe, cations and anions. A series of stereoisomers are possible for the outer MOP host owing to (anti-)clockwise orientation of triazatruxene ligands around the center of the *T*_d_ face in addition to conformations (Δ or Λ) arising due to arrangements of tris-chelated octahedral vertices of the tetrahedron [327,328]. Interestingly, ^1^H NMR of this MOP displayed only one set of ligand signals pertaining to exclusive formation of a pair of *T*-symmetric tetrahedral enantiomers. The crystal structure of this MOP indicated that anticlockwise (*A*) triazatruxene always paired with Δ of iron(II) stereocenters while the clockwise (*C*) analog paired with Λ, thus leading to the generation of stereoisomers as Δ_4_-1 and Λ_4_-1, respectively. The cage-in-cage complex was formed in acetonitrile by treating CRY (*rac*-CRY) with MOP for 12 h at 70 °C. A stereoselective encapsulation supported by crystal structure led to the formation of a mixture of two enantiomeric host-guest complexes, namely *PP*-CRY⊂Δ_4_-1 and *MM*-CRY⊂Λ_4_-1. The binding constant of MOP for CRY (1:1 host-guest complex) was indirectly determined to be *K*_a_ = (9.5 ± 0.4) × 10^6^ M^−1^. The binding affinity of CRY⊂MOP for cesium cations was tested in solution since it is a known guest for other cryptophanes [153]. Indeed, a Cs^+^ ion was bound to CRY⊂MOP as indicated through the new set of signals observed in ^1^H and ^133^Cs NMR (bound resonance: −316 ppm, free resonance: 32 ppm). The binding constant was determined as (34 ± 3) M^−1^, respectively. The MOP was found to slow down the Cs^+^ exchange within the encapsulated CRY. Without the MOP, the exchange appears fast on the NMR time scale. ^129^Xe NMR of CRY⊂MOP in acetonitrile indicated two peaks at 179 (free Xe) and 47 ppm ([Xe⊂CRY]⊂MOP). ^129^Xe EXSY measurements revealed an exchange rate of 11 ± 2 Hz between [Xe⊂CRY]⊂MOP and free Xe, respectively. ^1^H NMR after encapsulation of enantiopure *PP*-CRY by MOP showed peaks corresponding to two diastereomeric host-guest complexes, namely *PP*-CRY⊂Δ_4_-1 and *PP*-CRY⊂Λ_4_-1 (1:1 ratio), respectively. The high enantioselectivity of binding between diastereomers of CRY and MOP was proven by 530 times higher affinity of (9.5 ± 0.4) × 10^6^ M^−1^ between *PP*-CRY and Δ_4_-1 than between *PP*-CRY and Λ_4_-1 (1.8 ± 0.1) × 10^4^ M^−1^, respectively. Using ^133^Cs or ^129^Xe NMR, the two diastereomeric complexes (*PP*-CRY⊂Δ_4_-1 and *PP*-CRY⊂Λ_4_-1) can be delineated through the observed peaks (^133^Cs NMR: 256, 311 ppm; ^129^Xe NMR: 47 and 54 ppm) of the nested hosts. These findings suggest that the outer MOP, depending upon its stereochemistry, can either strengthen or weaken the shielding effect of the inner CRY core as manifested in two different NMR-based measurements [329].

**Table 2:** Different important details pertaining to various ^129^Xe hosts.

Xe Hosts	Solvent and Binding Guests	Chemical Shift (ppm)	Binding Constant (M^−1^)	Xe HyperCEST Effect (ppm) and HyperCEST MRI
**Cryptophanes**				
Cryptophane 111	Xe in (CDCl_2_)_2_	~31	28,000 (278 K)	
(1). [(Cp*Ru)_6_(Cry111)]Cl_6_	D_2_O	308 (293 K)		
Cryptophane 222 (CryA)	CH_2_Cl_2_		475 (298 K)	
	CHCl_3_		230 (298 K)	
	CH_4_		130 (298 K)	
	Xe in (CHCl_2_)_2_		3000	
(1). hexahydroxy substituted (*MM*)-CryA derivatives	Cs^+^ in H_2_O		6 × 10^9^	
(2). hexahydroxy substituted (*PP*)-CryA derivatives	Cs^+^ in H_2_O		2 × 10^9^	
(3). triacetic acid functionalized CryA derivative	Xe in H_2_O		33,000 ± 2000	
(4). Trifunctionalized CryA	Xe in H_2_O		4.2 × 10^4^	
(5). CryA	Xe in (CDCl_2_)_2_	~62 (278 K)	3.95 × 10^3^ (278 K)	
(6). hexaacid functionalized CryA derivatives	Xe in D_2_O	64 (293 K)	6800 (293 K)	
(7). PEG-modified CryA	Xe in H_2_O	77		
(8). CryA-linker-biotin (B1)	B1 (300 μM) and ~80 nmol avidin	193 (Xe@aq), 70 (Xe@CryA), 2.3 ppm downfield to bound Xe (avidin-bound B1)		
(9). cysteine-maleimide by a lysine in B1 (B2)	B2 (140 µM) and 29 µM avidin	*RL* and *LL* diastereomeric peaks at 65.1 and 64.5 ppm shifted to 67.9 and 66.4 ppm		
(10). B2 with rigid linker (B3)	B3 (77 μM) and 20 μM avidin	four peaks with large line widths (45–55 Hz)		
(11). B2 with short linker (B4)		increased both line width and chemical shift sensitivity		
(12). Biosensor B3	250 μL avidin-agarose beads	65.4 (Xe@B3), 193.6 (Xe@agarose), 192.5 (Xe@aq)		
(13). CryA-based CD14 biosensor	cells			~120 ppm only for RAW264.7 cells
(14). CryA-based claudin biosensor	cells			59% CEST effect for transfected HEK cells compared to 11% in non-transfected HEKs. Xe@CryA in lipidic/cellular environment (~120 ppm)
(15). CryA-dicarboxylic acid derivative based biosensor	H_2_O + 1% DMSO			−131.2 ppm
(16). CryA-monocarboxylic acid	H_2_O + 1% DMSO			−132.4 ppm
(17). CryA-based MMP7 biosensor	D_2_O	61.8 (*RL*) and 62.4 (*LL*) diastereomers. Two groups of two peaks separated by 0.6 and 0.8 ppm observed for mix of uncleaved and cleaved MMP7		
(18). CryA-based folate biosensor	acetate buffer (pH 5)	64.8 (*LRL*) and 66.0 (*LRR*) diastereomers		
(19). Trisubstituted CryA derivatives (subst. = tripropargyl, triallyl, tribenzyl, trihydroxy)		63–65 ppm, 57 (trihydroxy substituted CryA)		
(20). CryA-based pH biosensor	solution and cells			64.2 (pH 7.5), 67.6 (pH 5.5) at room temperature. In HeLa cells: 78.4 ppm (pH 5.5), 65 ppm (pH 7.5)
(21). CryA-based integrin biosensor	Tris buffer (pH 7.2)	65.8, two resonances at 67.1 (free CryA) and 71.2 (bound CryA) on treating α_IIb_β_3_ (16 μM) with biosensor (50 μM).		
(22). CryA-based carbonic anhydrase biosensor (triazole linkers with 6-, 7- and 8-bonds to benzenesulfonamide forms C6B, C7B and C8B ligands)	H_2_O	bound Xe peaks for C6B, C7B and C8B are at 63.5, 63.9 and 62.9, respectively.		
(23). Tripropargyl CryA-based Calmodulin biosensor	buffer	biosensor + eq.molar CaM (*halo* form) showed two peaks for bound Xe (65.9, 67.6).		
(24). PEGylated tripropargylated CryA derivative	H_2_O (20 mM solubility)	bound Xe at 77.4 ppm		
(25). Zn^2+^ chelating hexacarboxylic acid CryA derivative	Sensor (260 μM) in PBS buffer	65.6 (bound Xe), Zn^2+^ addition splits bound Xe peak into 65.75 and 67.2.		
(26). (+)-*MM*-cryptophane-(*L*)-NTA	Sensor (970 μM) in HEPES buffer	5% Zn^2+^, 5% Cd^2+^, 5% Pb^2+^ indicated downfield shift of bound Xe i.e., 1.5 (Zn^2+^), 0.3 (Cd^2+^), 4.5 (Pb^2+^)		
(27). CryA-based porphyrin sensor	solution and cells			CEST peak at 72 ppm (between pH 3 and 5). pH increase from 5.1 until 9.3 showed another peak at 70 ppm. A549 cells incubation with sensor indicated CEST peak at 74 ppm (pH 5.1 and 7.4).
Cryptophane 223 (Cry223)	Xe in (CDCl_2_)_2_	52	2810 (278 K)	
(1). hexaacid derivative of Cry223	Xe in D_2_O	42	2200	
(2). Cry223 (R_1_^1−6^ = CH_3_, R_2_ = OH)		64		
(3). Cry223 (R_1_^1−6^ = R_2_ = CH_2_CO_2_CH_3_)	(CDCl_2_)_2_	82		
(4). Cry223 (R_1_^1−6^ = CH_2_CO_2_CH_3_, R_2_ = OH)		58		
(5). Cry223 (R_1_^1−6^ = CH_2_CO_2_H, R_2_ = OH)		50		
(6). Cry223 (R_1_ = R_2_ = OH)		63		
(7). Cry223 (R_1_^1−6^ = PEG, R_2_ = OH)		67		
(8). Cry223 (R_1_^1−6^ = CH_2_COOH, R_2_ = CONHOH)		58.4 (pH 9.9), 59.4 (pH 8.2), 61.3 (pH 3.3)		
Cryptophane 333	solution	35		
**Cucurbit[n]urils**				
Cucurbit[6]uril (CB[6])				
(1). CB[6]-Na^+^ coordination	THF encapsulation		5.1 ± 0.5 × 10^2^ at room temperature	
(2). CB[6]-Cs^+^ coordination	THF encapsulation		1.1 ± 0.1 × 10^3^ at room temperature	
(3). CB[6]	H_2_O, acidic or saline solution	122 ± 0.5 (bound Xe signal), 199 ± 0.5 (free Xe signal)		
(4). Cy_6_CB[6] (Cy = cyclohexane)	H_2_O	93 (bound Xe peak), T_1_ ~40 s	1.3 ± 0.1 × 10^3^	
(5). CB[6]-based rotaxanes	solution			rotaxane (100 μM): no Xe@CB[6] signal. Base-catalyzed hydrolysis led to semi-rotaxanes and appearance of Xe@CB[6] signal.
(6). CB[6]-based MMP2 sensor	rotaxane (5 μM), MMP2 (5 nM)			cleavage of rotaxane led to CB[6] release from former’s axle (15% CEST effect)
(7). CB[6]-based H_2_O_2_ sensor	rotaxane (25 μM), H_2_O_2_ (50 μM)			cleavage of rotaxane led to an apparent maximum saturation of about 25%
Cucurbit[7]uril (CB[7])				
(1). CB[7]-based biotin biosensor				−68 ppm Xe@btCB[7] similar to free CB[7]. Avidin to btCB[7] (50 μM) led to four peaks at −68 ppm (Xe@*i*btCB[7]), 0 ppm (Xe_aq_), 100 ppm (Xe@avidin) and −40 ppm (Xe@btCB[7]-avidin). CB[7] interaction with avidin estimated to generate 50% CEST difference.
**Pillar[n]arenes**				
Pillar[5]arenes	cationic viologen salt		8 ± 2 × 10^4^	
(1). water soluble pillar[5]arenes decorated with carboxylate groups	amino acids e.g., L-arginine, L-lysine, L-histidine		ca. 10^3^	
(2). pillar[5]arenes with 10 trimethyl ammonium groups	sodium alkyl sulfonates e.g., 1-octane sulfonate		1.3 ± 0.9 × 10^4^	
(3). Pillar[5]arene	hexane as thread			more than 75 ppm observed compared to external reference (Xe solubilized in CDCl_3_) 10 ppm downfield for Xe compared to Xe@pillar[5]arene.
**Metal organic polyhedra (MOP)**				
Fe-MOP	Fe-MOP (8.9 mM), Xe (20 mM) in D_2_O	Xe@Fe-MOP appeared at 16.6 ppm downfield than free Xe in D_2_O	16	Xe exchange rate in Fe-MOP is 10 Hz.
**Supercage**				
Cry 111 inside Fe^II^_4_L_4_ MOP		Cs^+^ binding to Cry⊂MOP indicated bound resonance (316), free resonance (32, ^133^Cs NMR)	MOP for Cry binding constant is 9.5 ± 0.4 × 10^6^ Cs^+^ binding to Cry⊂MOP revealed 34 ± 3	Cry⊂MOP showed 179 ppm (free Xe), 47 ppm ([Xe@Cry]⊂MOP]) and an exchange rate of 11 ± 2 Hz.
**Liposomes**				
(1). CryA-dicarboxylic acid derivative (CryA-da)	CryA-da (15 ± 5) μM in lipid emulsions	62 (Xe@cage), 189 (Xe_aq_) Increasing lipid conc. between 1 and 5 % led to the appearance of two resonances in aq. solution and third one at 73 ppm. Chemical shift difference between Xe signals in aqueous and lipid environments is larger inside CryA-da (~10 ppm) than unbound Xe (~1 ppm).		
(2). Targeted liposomes decorated with lipopeptide	cells			HBMEC cells (target cells) + liposomes resulted in two peaks at 71 ppm (Xe@CryA@cells) and 62 ppm (Xe@CryA@aq) compared to reduced signal in control HAoEC cells. ^129^Xe HyperCEST intensity of (68 ± 4)% in HBMECs compared to (23 ± 5)% for control cells
(3). CryA-monocarboxylic acid (CryA-ma) in liposomes	CryA-ma (5 μM) + POPC or DPPC (200 μM) at 300 K			64 ppm (Xe@CryA-ma@aq), 74 ppm (Xe@CryA-ma@lipid)
(4). POPC/cholesterol model membranes	CryA-ma loading into liposomes			70 ppm (Xe@CryA-ma@aq), ~77 ppm (Xe@CryA-ma@lipid)
**Biogenic scaffolds**				
MS2 viral capsids	CryA (1 μM): 70% modification of MS2 proteins i.e., ~125 copies of CryA per capsid.			190 ppm (Xe_aq_), 60 ppm (bound Xe peak)
	CryA, fluorophore installed at capsid interior (~110 CryA per capsid) and exterior decorated with DNA aptamer TD05.1			scaffold (167 nM in capsids) incubated with Ramos (positive cells) showed a broad Xe@CryA peak at 57 ppm and 50% higher image contrast compared to control Jurkats.
M13 bacteriophage	p8 proteins modified with PEG5k (approx. 28% i.e., 760 copies) and CryA (approx. 39% i.e., 1050 copies)			sensor (2.3 nM, 293 K) revealed peaks at 192 ppm (Xeaq), 61.8 ppm (Xe@CryA@M13), 59.4 ppm (Xe@CryA). At 310 K led to changes in the observed peaks i.e., 192.4 ppm (Xeaq), bound Xe 64.6 ppm (Xe@CryA@M13), 62.2 ppm (Xe@CryA).
fd bacteriophage (EGFR targeted biosensor)	p8 proteins modified by 6–8% (~330 cages per phage)			Target cells (MDA-MB-231) incubated with fd-CryA sensor (0.7 nM) exhibited a bound peak at 70 ppm and 16.0 ± 9.4% contrast compared to control Jurkats.
**Gas binding protein structures**				
(1). TEM-1-beta-lactamase (bla, 29 kDa)			40	CEST of recombinant bla (80 μM) showed two peaks at 195 ppm (Xeaq) and 255 ppm (Xe@bla)
(2). Maltose binding protein (MBP)				HyperCEST response for bound Xe appeared at 95 ppm (Xe@MBP) after treating MBP (80 μM) with 1 mM maltose.
**Superhosts**				
Perfluorooctyl bromide (PFOB) nanoemulsions	PFOB (130 nm)			Two CEST peaks found at 111 ± 9 ppm (Xe@PFOB) and ~192 ppm (Xeaq)
Targeted PFOB				106 ppm (PFOB-dissolved Xe) if particle size larger than 250 nm and conc. of 5 pM. Target cells (A549) incubation with RGD-tagged PFOB led to CEST peak at 106 ppm and 54% HyperCEST effect compared to no uptake by control cells (WI-38).
Gas vesicles (GVs)	145 nm diameter, 250–1000 nm length			CEST peak at 31.2 ppm (Xe@GVs), 195 ppm (Xeaq) and saturation contrast of about (33 ± 2)%. GVs engineered from *Halobacteria sp. NRC-1, Microcystis SP* and *E. Coli* exhibited following CEST responses at 14.4, 30.6 and 51.4 ppm.

### 5.9. Nanoscopic Formulations as Multivalent Xe Hosts

While the initial Xe biosensor design focused on the conjugation of individual, synthetic hosts to targeting units (biotin, peptides, antibodies etc.), additional applications emerged where such hosts were embedded into nanoscopic formulations such as liposomes. Nano-droplets of gas binding substances also fall into this category where many Xe atoms (up to thousands) can bind to nanoscopic structures. These become modular scaffolds that can be decorated with targeting moieties for different applications.

#### 5.9.1. Liposomes and Micelles as Modular Platform

Liposomes are spherical entities that are self-assembled from phospholipids of natural and synthetic origin (sometimes in combination with cholesterol) such that they can act as an excellent carrier platform for different probes aiming at in vivo applications [330,331,332,333,334]. Liposomes generated with sizes ranging between 50 and 200 nm are reported to enhance blood circulation of loaded probes and subsequently lead to the accumulation at target tissues (e.g., tumors) via “passive” targeting promoted by an enhanced permeation and retention (EPR) effect. Installation of functional moieties (e.g., targeting ligands, antibodies) at the liposome surface leads to the generation of different “active” targeted liposomal formulations. Although the particle size does not have a direct effect on the targeting behavior of liposomes, nanosized versions of the latter are pivotal for enhancing the interaction with desired tissue or organs [335]. Liposomes are often prepared by employing one of the methods such as thin-film hydration, reverse-phase evaporation, ethanol injection, or microfluidics technology, respectively [330]. Use of sonication or extrusion is often necessary while using the aforementioned first three methods in order to restrict the generated liposomes sizes to 50–200 nm.

Incorporation of different probes of interest into liposomes is usually performed by utilizing either passive or active loading. In terms of the passive loading, the probes (hydrophilic or hydrophobic) are added into the appropriate media (e.g., organic or aqueous) along with the phospholipids prior to the generation of liposomes. This type of loading is almost applicable to every probe, however, the encapsulated efficiency varies substantially depending on the structure of the probes and liposomal formulations. Conversely, the active loading is performed by subjecting the preformed liposomes to probe loading after incorporating the former with hydrophobic chelators via passive loading. The active loading method is more complicated and its applicable only for limited probes. However, encapsulation efficiency achieved through this method is greater than 90%. Liposomes find applications in the fields of diagnostics, therapeutics and in image-guided drug delivery, respectively [336]. As such, liposomes cannot behave as imaging probes and they display such properties only upon incorporation of imaging reporters. All liposomes destined for in vivo studies are often decorated with PEG units in order to limit their uptake by RES and to promote enhanced cellular uptake and accumulation in the target tissue. A review on the lipid compositions of different liposome-based probes utilized for in vivo applications indicated that these were mostly designed with DPPC, cholesterol and DSPE-PEG2k as the main components [337]. The molar ratio between DPPC and cholesterol in different formulations varied a lot albeit the DSPE-PEG2k ratio remained between 4–6%. Notably, the DSPC- and HSPC-based formulations are more stable than DPPC-based ones under in vivo conditions while the EPC- and DOPC-based formulations are less stable due to their lower transition temperatures, respectively [337].

In the context of MRI, liposomes have been utilized as carriers for NMR reporters, e.g., paramagnetic Gd or iron oxide nanoparticles in order to attain reduced systemic toxicity, extended circulation, and an increased contrast enhancement for the loaded reporter [338]. For example, the folate expression in HeLa cells was mapped effectively by using folate-conjugated, Gd-containing liposomes via ^1^H NMR/MRI compared to the control Gd-DTPA [339]. Similarly, a safe alternative *T*_1_-MR contrast agent, e.g., Fe-succinyl deferoxamine/doxorubicin (DOX) encapsulated in liposomes has been utilized to determine the amount of DOX released onto the tumor via the change in the relaxation rates [340]. Additionally, ferri liposomes are useful to achieve high resolution *T*_2_-weighted MR images of mice tumors (e.g., breast xenografts) [341]. The efficacy of liposomes loaded with imaging probes can be improved by increasing stability through optimization of the lipid constitution or by crosslinking the lipid layer in order to avoid pre-leakage of the encapsulated probes into serum during systemic circulation. Additionally, an undesired uptake into the liver can be prevented by administration of blank liposomes prior to the targeted ones such that an enhanced accumulation in the target tissue will be achieved [342].

Although liposomes can be loaded with (passive) relaxation reporters for enhancing the achieved contrast in tissue water, generating a distinct NMR signature that is directly associated with liposomes is of great interest. To this end, CEST can be utilized as a tool for probing the intrinsic characteristics of liposomes that are capable of generating MR contrast. An example are paraCEST agents that were utilized to derive another class of reporters, namely lipoCEST agents. In such liposomes, H_2_O entrapped inside the liposomal aqueous environment (inner compartment) represents an exchangeable pool of protons that undergoes exchange with the bulk water proton pool (outside the liposomes). However, unloaded liposomes themselves cannot be used as a CEST agent since the chemical shift of H_2_O available in both compartments is indistinguishable. Therefore, it was necessary to introduce a lanthanide shift reagent (LSR) into the inner liposomal compartment (LipoCEST) to promote fast exchange of H_2_O from inner to outer compartment. A detection threshold of 5% CEST level can be reached by using lipoCEST reporters in the pM range [343]. Tm(III) and Dy(III)-DOTA (LSR) complexes could generate only a few (+4 and −4) ppm shift. To attain even higher shifts, aspherical compartments in liposomes have been proposed for encapsulating H_2_O [344]. The exchange rate of H_2_O across the liposomal membrane of aspherical liposomes increases due to increased surface/volume ratio of vesicles and disordered phospholipids (PL) assembly in the bilayer. For example, spherical liposomes encapsulating neutral LSR [Tm(hpdo3a)] (40 mM) and incorporating an amphiphilic Tm complex (30 mol %) subjected to osmotic stress resulted in aspherical liposomes reaching an H_2_O chemical shift of δ = 20 ppm compared to δ = 0.4 ppm for spherical ones. Using erythrocytes (RBC) as “aspherical or shrunken lipoCEST agents” labeled with Dy-HPDO3A under hypo-osmotic conditions led to 6.5 ppm shift and 65% CEST effect in vivo [345]. Even though the (aspherical) lipoCEST exhibited a considerable improvement over the Gd-loaded/conjugated liposomes, there is still a growing interest in having a metal free MR contrast enhancement using liposomes.

**Figure 11:**
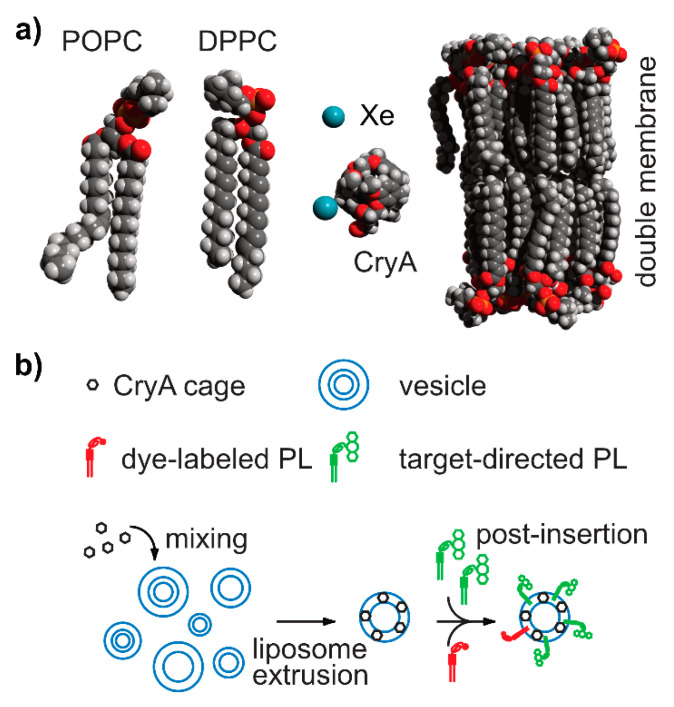
Lipid membrane components and functionalized liposome production for HyperCEST applications. (**a**) POPC with a kinked hydrocarbon chain yields more loosely packed membranes than DPPC with saturated, straight chains. A double membrane layer in comparison next to a lipophilic CryA cage. (**b**) Procedure for generating functionalized liposomes that carry CryA cages. Optional units are phospholipids with a dye unit or a binding moiety for a certain molecular target.

Such a metal free NMR/MRI signature from liposomes can be realized in terms of ^129^Xe HyperCEST to attain even higher signal enhancement compared to the aforementioned conventional ^1^H NMR/MRI. Since Xe is highly sensitive to its molecular environment, it was successfully utilized to investigate the composition, fluidity, and domain fluctuations occurring at the phospholipid bilayer. The interaction anticipated between different hydrophobic players such as Xe, CryA (as Xe host), and the phospholipid environment was evaluated using model lipid membranes made of DPPC and POPC (see Figure 11a) to reveal different Xe characteristics (e.g., exchange rate, partitioning, detection of CryA in aqueous/lipid environment etc.) experienced at the phospholipid bilayer. The interaction between CryA-da and lipid vesicles was evaluated using ^129^Xe NMR. CryA-da ((15 ± 5) µM) for self-insertion into the bilayer initially displayed two Xe resonances at 62 ppm (Xe@cage) and 189 ppm (Xe_aq_), respectively. Exposing 1, 2, or 5% lipid suspension to CryA-da led to three resonances in each case, i.e., two in aqueous solution and a third one at 73 ppm (attributed to Xe@cage in the lipid phase). For a lipid fraction of 10%, only the cage resonance at 73 ppm was observed, which might occur due to preferential partitioning of CryA-da and Xe into the lipid environment. Interestingly, the chemical shift separation between Xe signals in aqueous and lipid environment is bigger (~10 ppm) inside CryA-da than for uncaged Xe (~1 ppm). The saturation experiments performed at frequencies corresponding to above Xe peaks indicated a more rapid exchange of Xe with the host in the lipid phase than in H_2_O. For temperatures up to 25 °C, the two signals of Xe@CryA-da in lipid and in aqueous environment can be separated. Above 25 °C, the two peaks overlapped and remained indistinguishable. Additionally, the chemical shifts of Xe@cage_aq_ and Xe@cage_lipid_ increased linearly with temperature. Higher solubility of both Xe and CryA-da in the lipid environment along with fast exchange led to generation of an efficient lipid-based HyperCEST agent [346].

The interaction of fluorophore-labelled CryA with phospholipid membranes was also investigated with ^129^Xe HyperCEST NMR in combination with Förster resonance energy transfer (FRET) with a donor dye inside the membrane. This helped to understand the interaction between the CryA-conjugate and hydrophobic membranes and gave insights regarding complete or partial insertion of the conjugates into the membranes. The two CryA-dye conjugates utilized in this study were mixed with lipid vesicles (125 ± 30 nm) prepared from different phospholipids (POPC, EYPC, DPPC) and HyperCEST measurements revealed a large difference in signal intensities for different CryA-dye conjugates compared to no observable difference using fluorescence. A higher CEST signal was observed for POPC vesicles compared to other vesicles (e.g., EYPC, DPPC) which is attributed to a combination of a faster Xe exchange rate, a shorter *T*_2_, and an increased concentration of encapsulated Xe in CryA. Increasing the temperature to 60 °C exhibited a downfield chemical shift for all observed peaks (~2–3 ppm) and overall lower absolute signal intensities (ca., 50%). However, line broadening occurring for the relatively fast Xe exchange occurring inside the membrane with the CryA conjugate makes it difficult to distinguish between different membrane types. In spite of evaluating the chemical shift for various lipid vesicle compositions, the phospholipid intrinsic characteristics and membrane-specific Xe interaction can be utilized to distinguish different lipid membranes (see Section 5.8.2) [233].

An advanced version of liposome-based reporters can be obtained by functionalizing the vesicle membrane. Self-insertion of lipopeptides (for presenting targeting moieties at the membrane surface) and of lipophilic Xe hosts is a straightforward strategy for this purpose (see Figure 11b). In this case, further chemistry on the lipophilic Xe host becomes obsolete as this simply partitions into the membrane. Such host-carrying liposomes can be obtained through POPC-based large unilamellar vesicles (LUVs) that are treated with CryA such that ca. 3800 CryA units were loaded into each liposome. Careful CryA loading into LUVs did not alter their structural integrity (diameter: (116 ± 2) nm) as long as the concentration ratio of [CryA]:[POPC] did not exceed a ratio of 1:20. The targeted liposomes were prepared by post-insertion of cationic lipopeptide P2Rn (arginine (R) rich peptides) into POPC LUVs (CryA + LUV + P2Rn), leading to a marginal increase in vesicle size (diameter: (125 ± 3) nm). These R-rich lipopeptides are readily internalized by the cells lining the blood brain barrier (BBB) via the clathrin- and caveolin-independent endocytotic uptake mechanisms such as receptor-mediated uptake and through the interactions with negatively charged cell surface heparan sulfate proteoglycans [346,347,348,349,350]. This modular approach was beneficial in generating targeted liposomes for Xe NMR/MRI without the need for elaborate synthesis. The toxicity of non-targeted (CryA+LUV) and targeted (CryA + LUV + P2Rn) counterparts on the brain microvascular endothelial cells (HBMECs) and the control human aortic endothelial cells (HAoECs) indicated significantly reduced toxicity effect compared to administration of bare CryA. Neither the incorporation of CryA cargo nor the positively charged P2Rn on the liposomes indicated any significant toxicity within the applied biosensor concentration. Fluorescent tags such as rhodamine-labeled lipids (Rh-PE) in combination with carboxyfluorescein-labeled P2Rn were introduced into the membrane such that the fate of the LUVs and loaded cargo can be visualized independently. Colocalization of both fluorescent tags in HBMECs revealed the intactness of LUVs without any degradation even after 4 h. Additionally, the liposomes labeled with the lipopeptides accumulated in distinct intracellular compartments of the cells in agreement with earlier reports [347]. Similarly, flow cytometry-based quantification indicated that LUV + P2Rn exhibited 7.4-fold higher uptake in HBMECs than for the control liposomes. The specificity of CryA + LUV + P2Rn for HBMECs cells was further confirmed through Xe HyperCEST NMR and MRI measurements. Xe HyperCEST of HBMECs with targeted liposomes showed two peaks, namely at 71 ppm (Xe@CryA@cells) and 62 ppm (Xe@CryA@aq). This former signal also occurs in control HAoECs, albeit with significantly reduced intensity due to the less efficient uptake. Also, ^129^Xe HyperCEST MRI of HBMECs indicated a high mean HyperCEST effect (68 ± 4)% compared to (23 ± 5)% for control cells. This demonstrates that liposomes loaded with CryA and labeled with a targeting peptide will foster high sensitive detection of cell-specific surface interaction [131].

In cases where the core of such liposomes is not used for drug cargo, a rather large fraction of the particle volume is not used for any function. Since the membrane volume can carry only a limited number of cages, such particles can be further optimized in terms of their invasive cell labelling volume. A recent study introduced a lipopeptide that forms size-optimized micelles with a diameter of about 11 nm at low micromolar concentration [351]. It provides a high local CryA payload that is ca. 7-fold higher than the cage/volume ratio of the liposomes. Importantly, the introduction of CryA did not hamper the micelle aggregation behaviour. Xe access to the hosts inside the micelle is efficient and does not require lateral diffusion to cage units like in the case of the liposomes. This easy access enables a strong and switchable impact on the bulk magnetization. The peptide decoration of the micelles foster preferential uptake into endothelial cells of the blood brain barrier. Altogether, this type of cell labelling is ca. 16,000-fold more efficient than ^19^F cell labelling.

#### 5.9.2. Biomembrane Fluidity Studies

Although Xe is highly soluble in lipid environments [352], determining its mobility in lipid-aqueous conditions using conventional relaxivity or diffusion is challenging due to relative fast exchange occurring between membrane-embedded Xe and Xe in aqueous phase. Therefore, an approach has been implemented in which the intra-membrane mobility of the atoms is probed indirectly. To this end, the build-up of Xe spin depolarization through membrane-embedded CryA-ma cages was investigated. This separates the Xe spins into two spectrally distinct populations [233,346], thereby facilitating selective depolarization through rf irradiation inside the membrane. The induced depolarization accumulates in the non-caged Xe pool outside the membrane and it causes the respective signal to decrease. Overall, Xe from aqueous environment has to penetrate into the membrane, followed by interaction with the host cages for depolarization, and subsequent exchanging back into the aqueous pool while carrying along the depolarization information. The differences in the membrane fluidity are anticipated to influence the efficiency of this process revealed through the intensity and build-up of the CEST response.

Our lab induced Xe depolarization inside CryA-ma embedded into a phospholipid bilayer using on-resonant saturation pulses with increasing saturation times. In this context, Bachert and co-workers have shown that such enhanced Xe depolarization process occurs in a mono-exponential way as follows [353]:

(1) $$f\left(\tau ,t_{sat}\right)\;=\;e^{-t_{sat}/\tau \left(k,T_{2},f\right)}$$

Here, τ is the depolarization time and depends on parameters like the Xe exchange rate *k*, the relaxation time *T*_2_ and the local concentration ratio *f* = [Xe@CryA-ma]/[Xe@solution].

Even though the encapsulated Xe in both vesicles (POPC/DPPC) display similar chemical shifts, the different time constants τ displayed by two biomembranes can be used to produce a new tunable MRI contrast. An inverse Laplace Transform (ILT) was applied to isolate the time constants from exponential signal loss to yield a method coined depolarization Laplace transform analysis (DeLTA). This algorithm allowed extracting the τ’s resulting from the (multi-)exponential decays. The HyperCEST responses of CryA-ma (5 µM) with either POPC or DPPC (200 µM) at 303 K indicated two CEST resonances (64 ppm (Xe@CryA-ma@aq) and 74 ppm (Xe@CryA-ma@lipid), respectively. The CEST response of POPC appeared much larger in intensity compared to DPPC. Subsequently, a CEST spectrum of a double-phantom containing POPC (inner compartment) and DPPC (outer compartment) gave a combined response from spectrally overlapping signals emerging from both biomembranes. Applying the DeLTA method revealed the Xe dynamics in two neighboring molecular environments by acquiring MR images of the phantom with saturation pulses at 74 ppm with variable *t*_sat_ (10^−3^–20 s) times. Increasing the *t*_sat_ resulted in faster depletion with POPC (τ _POPC_ = $3.00_{-0.61}^{+0.77}\;$*s*) in the inner compartment compared to DPPC (τ_DPPC_ = $22.15_{-4.16}^{+5.19}\;s$) in the outer compartment. Application of the ILT algorithm to the entire phantom volume revealed two time constants, namely τ _POPC*_ = $2.23_{-0.73}^{+1.12}\;s$ and τ _DPPC*_ = $19.75_{-7.35}^{+11.60\;}s$. These were attributed to faster (inner compartment) and slower (outer compartment) depolarization profiles and to their differences in membrane fluidity since there were no contributions from other experimental parameters. The depolarization time was clearly shorter when the membrane fluidity was higher and it resulted in complete saturation of the HyperCEST signals even when applying shorter saturation pulses. Thus, the ILT-based approach enables to distinguish the membrane fluidity represented by different molecular environments via the different depolarization times [354].

DeLTA was also utilized to investigate the mixed compositions (variable DPPC/POPC ratio) and variable cholesterol levels of model membranes. Binary liposomes (DPPC/POPC) can organize as a liquid-disordered (*L*_d_) phase, a solid-ordered (*S*_o_) phase, or into a phase where both coexist (*L*_d_ + *S*_o_). The actual state depends upon the concentration ratio and temperature, but also the liposome preparation. Mixing DPPC and POPC in different molar ratios prior to liposome generation yields so-called “premixed liposomes”. In another method, pure DPPC liposomes are titrated with POPC-pure liposomes leading to the generation of so-called “mixed liposomes”. In a feasibility study, such liposomes displayed a size distribution of (97 ± 26) nm in HEPES buffer. In both cases, the liposomes were loaded with 5 µM CryA-ma to enable ^129^Xe HyperCEST experiments. The “premixed liposomes” gave a CEST signal from Xe@CryA-ma@aq at 62 ppm and Xe@CryA-ma@lipid at 73 ppm, where the latter changes according to the applied DPPC/POPC molar ratio. Applying DeLTA to the exponential decays resulting from saturating the Xe@CryA-ma@lipid resonance with increasing *t*_sat_ yielded τ = (14.2 ± 0.7) s for 100% DPPC compared to τ = (69 ± 3) s for 98% DPPC. This increased depolarization time was attributed to domain fluctuations that might have perturbed Xe diffusion and exchange, thereby leading to an inefficient CEST effect. Below 77% DPPC, the depolarization was significantly faster (τ = (2.3 ± 0.1) s) and fastest for pure POPC owing to stable liquid POPC domains. In the case of “mixed liposomes”, the capability of ^129^Xe HyperCEST in sensing two different types of vesicles was tested in solution. In this case, the CEST resonance of Xe@CryA-ma@aq was observed at 62 ppm for different DPPC concentrations and the intensity of Xe@CryA-ma@lipid resonance decreased monotonically with decreasing DPPC content (98–50%). These results suggest that upon mixing the two liposomes, there is no observable phase transition occurring between DPPC and POPC liposomes. Interestingly, DeLTA-based analysis indicated one τ for high DPPC content (≥77%), while the lower DPPC content (<77%) led to a second τ -component that was shifted compared to τ of pure DPPC and POPC due to the lack of any physical separation between the two phospholipids in “mixed liposomes”.

A liquid-ordered (*L*_o_) phase can be induced in lipid bilayers by incorporating cholesterol [355]. Generally, different cholesterol concentrations tune the phospholipid phase at 310 K such that the *L*_d_ phase (<15%), *L*_d_ + *L*_o_ phase (~15–45%), and at higher concentration only the *L*_o_ phase was observed [356]. Two CEST resonances were observed for POPC/cholesterol model membranes at ~70 ppm (Xe@CryA-ma@aq) and ~77 ppm (Xe@CryA-ma@lipid) where the intensity of the latter one decreased with increasing cholesterol levels (0–50%). Using DeLTA, it was found that (2.4 ± 0.2) s as τ for pure POPC changes to (8.9 ± 0.3) s for 50% cholesterol admixture. Overall, the change appears to depend linearly on the cholesterol content. This suggests that the DeLTA method might complement the HyperCEST technique in detecting the different compositions and phase transitions (*L*_d_ + *S*_o_ → *S*_o_) in DPPC/POPC liposomes, as well as cholesterol levels (5% accuracy) of model membranes [357,358].

### 5.10. Biogenic Scaffolds and Gas-Binding Proteins

Although the genetic engineering of biogenic scaffolds is somewhat tedious, it provides a myriad of functionalization possibilities in which different moieties (e.g., hosts, tags, drugs etc.) can be easily tethered onto such scaffolds. These modified scaffolds can provide an unprecedented increase in detection sensitivity and selectivity towards the target of particular interest. Specifically, the surface and interior of such scaffolds are amenable to functionalization at ease without any steric interference arising from adjoining amino acid residues. Additionally, site- and residue specific-based chemical activation are achievable at different biogenic scaffolds. Unlike for macrocyclic hosts, different chemistry strategies can be applied to biogenic scaffolds due to the presence of multiple and distinct amino acid residues that are susceptible to chemical modification. The scaffolds withstand different harsh chemical reaction conditions without disassembling the protein assembly that envelops the genetic material. The activation and modification efficiency of different scaffolds varies and depends on several factors, e.g., pH, activation agent, scaffold structure etc. In order to achieve a higher modification at the scaffolds, the activation strategy should be chosen mainly depending on the scaffold’s protein structure, e.g., cavities, open or buried residues etc. At the end, the stability of the modified scaffold over time should be duly considered prior to its usage for different applications.

The chemical modification of such biogenic (e.g., viral) scaffolds are categorized into four broad classes. In the first category, the inherent amino side chains are functionalized with appropriate entities of interest. For example, carboxylates of Asp and Glu might be activated using EDC, thereby promoting its coupling to amine containing moieties. Similarly, the phenol of Tyr and amine of Lys can be functionalized by coupling to diazonium salts (Tyr residue) and NHS esters or activated carboxylates (Lys residue), respectively [359,360]. The amine functionalities are shown to be most reactive and efficient in undergoing coupling reactions at the scaffolds. The second category is about modifying the N-terminal amines into aldehydes such that different bioorthogonal reactions can be performed [361]. In the third category, the chemical modifications are performed after the genetic incorporation of either Cys or unnatural amino acids with desirable new functionalities. As an example, genetically displayed N-terminal Cys was modified in different ways including native chemical ligation [362]. Additionally, incorporation of p-azidophenylalanine through phage display also provided an easily modifiable azide handle on the scaffold (e.g., phage) surface [363,364]. The modification efficiency might be limited in the event of solvent having restricted access to some of the scaffold residues. Such limitations can be circumvented by relying on the non-covalent modifications (fourth category) possible at the scaffold’s surface. Such modifications exploit either the negative charge on the scaffold surface (e.g., phage) arising from Glu, Asp acid residues in the N-terminal region or by building upon the amphipathic coat proteins, respectively [365].

#### 5.10.1. Capsid-Based Scaffolds

The MS2 viral capsid contains a single-stranded RNA in its genome and forms a symmetric icosahedral capsid structure generated from 180 protein-subunits [366]. The average size of this capsid is ca. 27 nm and the presence of 32 pores each with a radius of 1.8 nm on its surface fosters further modification [366]. The empty envelope is known to be resistant to pH fluctuations (range: 3–10) and to rise in temperature, respectively. The modification at this capsid can be enhanced by introducing a cysteine residue (C87) onto the sequence of each monomer in the interior portion of the coat [367]. Additionally, the exterior surface can be engineered to display an unnatural amino acid, e.g., p-aminophenylalanine (paF). For example, the aniline group from paF can be utilized for conjugating it to *N*,*N*-dialkyl-phenylenediamine derivative [368]. In yet another example, the porphyrin maleimide was installed on the interior of MS2 through a thiol-maleimide coupling at Cys87 (N87C). The exterior was attached to a DNA aptamer that targets cancer cells via NaIO_4_-mediated coupling reaction to paF. Thus, MS2 capsids functionalized with 180 porphyrins and ~20 copies of aptamer specifically targeted Jurkat cells and selectively killed significant cell amounts in 20 min after illumination [369].

Regarding MRI reporters, Gd(III) complexes were also fused to this capsid. While complexation with simple chelators, e.g., DOTA or DTPA, the Gd(III) yields fairly low relaxivity values of 4–5 mM^−1^s^−1^ at 60 MHz and 25 °C, higher relaxivities can be obtained upon reduced tumbling [370] due to attachment to larger structures [371,372,373,374]. For example, a very high relaxivity (*T*_1_ = 202 mM^−1^ s^−1^) was observed for coupling Gd complexes to coat proteins of cowpea chlorotic mottle virus (CCMW, 28.5 nm diameter) [375]. Similarly, attaching Magnevist (Gd-DTPA) onto the MS2 viral capsid exterior via lysine residue modification revealed a 3-fold increase in relaxivity (14–16.9 mM^−1^ s^−1^) compared to free Magnevist [376]. An improved contrast for Gd was explored through optimization of interior and exterior labeling [377,378] and by installing rigid linkers [379] at MS2 viral capsids using chelators based on high affinity hydroxypyridinone (HOPO) ligands. Internally modified capsids (via cysteine) and usage of more rigid linkers such as the *S,S* enantiomer of 1,2-cyclohexyl diamine revealed up to 2.5-fold higher overall relaxivity achieved via the combination of rigid linkers and higher Gd loading i.e., 7416 mM^−1^s^−1^ at 60 MHz compared to relaxivities obtained for lysine and tyrosine MS2-Gd-bioconjugates (2900 mM^−1^s^−1^).

Similarly, the MS2 capsids were employed as a platform for improving the ^129^Xe NMR HyperCEST conditions by appending more Xe hosts (CryA) cages onto it. A mutation (N87C) in MS2 capsids aided the introduction of a solvent-accessible cysteine at the capsid interior. To facilitate coupling at the cysteine, a linker containing a maleimide and taurine for increasing aqueous solubility of CryA, and an amine that can be coupled to CryA are synthetically assembled. The as-generated cage-linker was treated with N87C MS2 for 4 h at room temperature in 10 mM phosphate buffer at pH 7.2 to generate the MS2-cage construct (MS2CA). ESI-MS analysis of the MS2CA construct estimated the extent of modification as ~70% conversion that corresponds to ~125 copies of cage per capsid. The ^129^Xe NMR spectrum of CryA (1 µM) indicated a peak from Xe_aq_ at ~190 ppm and only by applying HyperCEST method the bound Xe peak was observed at ~60 ppm, respectively. The performance of MS2CA sensor (7 nM) was comparable to free CryA (1 µM), i.e., similar contrast enhancement was observed for both solutions. However, a downfield shift of Xe@cage peak and slight upfield of Xe_aq_ peak was observed in the spectrum. A significant line broadening of Xe@cage peak was observed for MS2CA (~5 kHz) compared to free CryA (~1 kHz), respectively. Screening different dilutions of MS2CA between 700 and 0.7 pM indicated 0.7 pM as the detection limit for Xe-based biosensors through HyperCEST method [216].

A targeted and doubly modified version of the MS2 viral capsid was generated as virus-like nanoparticles (VLN) for targeting a biomarker of interest. As discussed above, the MS2 underwent N87C mutations to create a series of 180 sulfhydryl groups facing the interior surface of each capsid following its assembly [367]. The interior of N87C-T19pAF capsids was first conjugated to CryA-maleimide (CryA-Mal) which in turn was tethered to five glutamic acids in order to increase the solubility of the conjugate. ESI-MS analysis of the conjugate revealed that 60% of the monomers were modified, corresponding to an average of 110 CryA molecules per capsid. To enable validation using flow cytometry, the cysteine residues in the interior were labeled with Oregon Green maleimide. The hydrophobic moieties (CryA, fluorophore) are installed at the capsid interior since it enhances the solubility of the tagged molecules. On the exterior, p-aminophenylalanine (pAF) residues were installed on the position 19 of each monomer resulting in 180 copies per assembled capsid using the Schultz amber suppression technique [367,380,381]. The DNA aptamer TDO5.1 targeting membrane-bound mIgM markers expressed highly in lymphoma cells [382,383] were modified with aminophenol groups at the 5′-termini such that it can be attached to aniline side chains of the pAF residues via oxidative coupling in the presence of sodium periodate [369]. SDS-PAGE analysis revealed approximately 30% of the 180 monomers were derivatized with oligonucleotides. Flow cytometry confirmed the specific binding of TD05.1-labeled capsids to mIgM expressing Ramos Burkitt’s lymphoma cells compared to almost negligible binding to control Jurkat cells, respectively. ^129^Xe HyperCEST NMR of Ramos or Jurkat cells (2 × 10^7^ cells) incubated for 1 h with the MS2-CryA-TD05.1 conjugate (167 nM in capsids) indicated a broad Xe@CryA peak at 57 ppm for the Ramos compared to no peak observed for Jurkat cells. A CEST comparison generated by comparing the off- and on-resonance saturation signals for each cell suspension as a function of saturation time indicated a detectability in the case of Ramos cells with a contrast of (69 ± 9)% at 20 s saturation. Similarly, ^129^Xe HyperCEST MRI of both cells exposed to MS2-CryA-TD05.1 conjugate at similar experimental conditions indicated a higher image contrast (~50%) compared to no contrast in the controls (e.g., Jurkat cells and cell growth media), respectively [384].

#### 5.10.2. Bacteriophage-Based Scaffolds

Filamentous phages are another type that can serve as scaffolds. They represent a structure in which a single stranded DNA is encircled by a long rod-shaped protein coat. These filamentous phages that infect *Escherichia coli* are most fertile and are termed as “Ff phages”. They are subdivided into f1, M13 and fd phages, respectively [385]. Their major coat protein, p8, is common among all the filamentous phage types and contains just 50 amino acids albeit its configuration might be slightly different. The p8 protein is composed of three distinct domains, namely a negatively charged hydrophilic N-terminal domain, an intermediate hydrophobic domain, and a positively charged domain that interacts electrostatically with the phage genomic DNA. The chemical or genetic functionalization is possible only at the N-terminal domain since it is exposed to the media [386]. Further, genetic engineering at major coat protein p8 is less common since its monomers can only tolerate the incorporation of 6–8 amino acids [387,388]. Additional proteins such as p3, p6, p7, p9 display co-constructive roles in the construction of the phage coat. The minor coat protein p3 has been explored extensively in phage display due to the accessibility of its N-terminus that facilitates insertion of various peptides including larger proteins [389,390]. The length of the DNA core determines the overall size of these pages. It can be altered through insertion and deletion of nucleic acids, e.g., for the p8 protein 2700 copies in M13 and 4200 copies in fd. Although the filamentous phages are capable of displaying different peptides, their modification at the surface is limited due to the lack of a wide range of chemically distinct amino acids. To enhance such modification, a new method was suggested to convert the p8 N-terminal amino group into a ketone group, followed by the final conversion into an alkoxyamine group. In this way, a fluorophore tag and polyethylene glycol (PEG) was tethered to the p8 phage coat, while the p3 displayed peptides or an antibody that directs the phage to a specific receptor highly expressed in cancerous cells [361].

M13 phages display a cylindrical shape with a diameter of 6.6 nm and length of about 880 nm, respectively. The single stranded DNA remains inside the cylindrical coat made out of p8 and five copies of other minor proteins (p3, p6, p7 and p9) at both ends of the cylinder, respectively. The p8 protein displays a helical structure and it was found recently that the average distances between two neighboring N-termini of p8 proteins are 3.2 and 2.4 nm, respectively [391,392]. In this case, the M13 phage can act as a platform to achieve site-specifically modified functional materials, i.e., modifications along the body or at the tip of the phage. The phage functionalization challenges might be surpassed by performing mild and facile chemoselective reactions at the phage. In general, M13 surface comprises different amino acid residues such as lysine, N-terminal (amine group), aspartic or glutamic acid (carboxylic group) and tyrosine (phenol groups) that are amenable to further functionalization with molecules of interest. The M13 can be chemically modified by choosing the appropriate functionalization strategies depending upon the available residues, e.g., amino-NHS coupling (lysine), CuAAC chemistry, alkyne-diazonium reagent (tyrosine) etc. Using phage display techniques, many M13 mutations were selected for achieving different applications such as high-power batteries [393,394,395], tissue regenerating materials [396], metal nanowire catalysts [397], biological sensors [398,399], gene transfer vectors [400,401] and targeted cancer therapies [402], respectively. Out of the different phage modifications strategies, often functional moieties are introduced onto the major coat protein p8 via the nonspecific modification of amine groups available on the phage surface using NHS esters [403,404]. An extensive and undesired acylation of many lysine residues on the p3 proteins and associated protein fusions is noted while pursuing the nonspecific way of amine modification. This led to a considerable heterogeneity and potential binding interference that occurred at higher modification levels. Alternatively, tyrosine residues functionalized by diazonium coupling reactions might lead to modification of the critical phage residues [359]. Another way to achieve phage specificity towards a molecular target is by attempting genetic modification of phage DNA to introduce new residues at p3 N-termini, e.g., serine, threonine. These residues can be oxidized with sodium periodate to produce an aldehyde suitable for further chemical labeling [405]. Although genetic approaches such as enzymatic ligations, e.g., biotin ligase [406] and sortase A [387,388], endorse more specificity than chemical-labeling approaches, this approach requires rigorous genetic engineering of phage DNA which might not be always feasible. To achieve a specific modification of the filamentous phage chemically and to introduce new functional groups, a two-step transamination/oxime formation approach was utilized [407,408,409,410]. This new approach is highly selective for N-terminal groups and the lysine ε-amines remain unaffected without undergoing any transamination. It was found that the transamination proceeds readily in the presence of N-terminal alanine residues available in proximity to the proximal lysine side chains [411]. Indeed, the phage p8 monomers possess a solvent-exposed N-terminal alanine and lysine, thus making it an interesting substrate for the transamination reaction.

To promote a biomimetic transamination reaction, the p8 protein of the M13 phage underwent a pyridoxal 5′-phosphate (PLP) mediated selective activation of the N-terminal amine such that ketone groups can be introduced at the latter after 18 h of reaction at pH 6.5 [407]. In order to improve solubility and bioavailability of the final construct, some of the converted keto groups are tethered to aminooxy-functionalized 5 kDa PEG (PEG-5k-ONH_2_) in the presence of aniline as a catalyst at pH 6.0 for 1 h (293 K). Subsequently, the PEG-modified M13 (M13-PEG) was allowed to react with an aminooxy-functionalized CryA-peptide (CryA-ONH_2_), catalyzed by aniline at pH 5.5 for 22 h (293 K) in order to achieve modification at some of the remaining N-terminal ketone groups. The resulting M13-PEG-CryA (M13-based biosensor) p8 proteins were approximately 28% modified with PEG-5k (760 copies) and approximately 39% modified with CryA (1050 copies), respectively. The CryA modification was controlled such that a desired high CryA payload could be attained without compromising the aqueous solubility of the whole biosensor. Assessing the CEST capabilities of the M13-based biosensor (2.3 nM) compared to “unscaffolded” CryA (2.2 µM) by ^129^Xe HyperCEST at 293 K indicated a Xe@aq peak at 192 ppm and bound Xe peaks at 61.8 (Xe@CryA@M13) and 59.4 ppm (Xe@CryA), respectively. With a 2.4 ppm downfield shift and increased width of the signal for Xe@CryA@M13 compared to Xe@CryA (which was anticipated due to slower tumbling of the supramolecular phage structure), CEST effects of 17% and 19%, respectively, were comparable for both samples. An elevated temperature of 310 K changed the chemical shifts of all peaks, i.e., Xe@aq appeared at 192.4 ppm and the peaks of bound Xe 64.6 ppm (Xe@CryA@M13) and 62.2 ppm (Xe@CryA), respectively. The saturation time curves at both 293 and 310 K indicated comparable contrast for both M13-based biosensor and the free CryA. However, at higher temperatures the contrast was generated more rapidly due to the faster chemical exchange dynamics: the residual signal was 90% and 42% for 293 and 310 K, respectively, after 5 s of saturation. Under very dilute conditions, the CEST contrast decreased roughly logarithmically in accordance with decreasing M13-based biosensor concentration for a given saturation time. Scaffolding of CryA onto the M13 phage improved the per-CryA sensitivity (230 fM) with a mean contrast of 3.7% which corresponded to 242 pM of free CryA in solution [412].

In a similar approach, the p8 protein of the filamentous phage fd (4200 p8 copies) was modified using PLP mediated transamination at pH 6.5 for 13 h. Subsequently, the activated fd phage was exposed to different alkoxyamine derivatives at pH 6.5 for 24 h in order to achieve fd-scaffolded constructs. The oxime formation between the activated fd phage and alkoxyamine derivative was accelerated by using aniline as a catalyst [413]. According to the extent of the modifications desired for the p8 protein, the reaction times and alkoxyamine concentrations were selected. The overall protein recovery after the transamination and oxime formation steps involved in fd phage modification ranged between 55 to 95%. The only byproduct noticed in this type of transamination reaction was a covalent adduct of protein with PLP formed in lower amounts, presumably by an aldol addition of N-terminal pyruvamide to the pyridoxal aldehyde group. The epidermal growth factor receptor (EGFR) served as a cellular target unit. It is a cell surface receptor which is highly expressed in solid tumors, e.g., breast cancer and it is also linked to cancer progression [414,415], making it a suitable target for diagnostic and therapeutic applications. The transaminated anti-EGFR, anti-HER2 and control anti-BoNT fd phage were treated with Alexa Fluor 488 or 647 C5-aminooxyacetamide (AF488/647-ONH_2_) dyes such that its specific binding to respective molecular targets can be followed via fluorescence. The p8 proteins of fd phage were labeled approximately at a fraction of 6–8% (~300 copies/phage) with the fluorophores. At higher p8 modifications (80%) the fd construct became less soluble in buffers. FACS results demonstrated the specific binding of fd constructs to respective cell surface receptors via endocytosis compared to no binding in the case of anti-BoNT phage control. To reduce non-specific binding and for increasing solubility of appended molecules on fd phages, 2 kDa PEG2k-ONH_2_ was installed on the ketone labeled fd phage leading to approximately 67% modification of p8 proteins. The PEG-labeled anti-EGFR fd showed binding to MDA-MB-231 (EGFR positive) cells compared to no binding at MCF-7 cl18 cells (EGFR negative), respectively [361].

Similarly, such fd phages can display single-chain antibody variable fragments (scFvs) for recognizing either epidermal growth factor receptor (EGFR) or a negative control (botulinum toxin serotype A, BONT) through engineering on their minor coat proteins (p3). This strategy could generate a highly sensitive ^129^Xe biosensor. Applying the same chemistry involving transamination/oxime formation to fd phages resulted in the generation of fd-CryA biosensors. On average, the fd phage was ~8% modified with CryA cages that corresponded to approximately 330 copies per phage. Fluorescence-based detection showed the specific uptake of fd-CryA biosensors by MDA-MB-231 cells (EGFR positive) compared to Jurkat cells (EGFR negative), respectively. Conversely, the negative control anti-BONT phage constructs indicated low binding to MDA-MB-231 cells which demonstrated that the modification with CryA did not lead to any non-specific binding. A direct ^129^Xe NMR spectrum of MDA-MB-231 cells (120 million cells/mL) incubated with fd-CryA biosensor (0.7 nM) showed a bound Xe peak at 70 ppm compared to no peak for control Jurkats. The MDA-MB-231 cells incubated with fd-CryA biosensor exhibited a (16.0 ± 9.4)% contrast compared to almost no contrast from Jurkat cells ((1.4 ± 4.6)%) when saturated for 10 s. In this study, the lowest detectable cell concentration was 50 million cells/mL and it gave a contrast of (6.4 ± 9.0)%. Further optimization of the detection technique, cellular labeling approach, and higher modification levels of the p8 protein are thus required prior to in vivo translation of such targeted fd phages [416].

#### 5.10.3. Gas-Binding Protein Structures as Xe Hosts

As mentioned in Section 2.1, various proteins offer potential Xe binding sites. Many of these systems exhibit relatively fast gas exchange of their natural gas loading, O_2_, and thus the residence time of Xe is also rather short: for metmyoglobin (metMb) methemoglobin (metHb), Xe remains ca. 10–20 ps inside the cavities and does not interfere with the overall protein structure and stability [23]. Such structural biology insights were revisited later for identifying options regarding exchange-coupled pools that could enable CEST applications. Protein structures that exhibit tighter binding of Xe represent a good starting point for protein engineering to obtain expressible HyperCEST agents. One example is TEM-1 β-lactamase (bla, 29 kDa) that was engineered from *E. coli* for this purpose [417]. Xe MRI contrast could be achieved in samples of both bacterial and mammalian cells expressing this protein. MD simulations revealed binding of Xe between two terminal α-helices and the flanking β-sheet. On the one hand, the experimentally observed exchange is slow enough to obtain a ^129^Xe HyperCEST spectrum of recombinant bla (80 μM) that comprises two peaks at 195 ppm (Xe@solution) and 255 ppm (Xe@bla), respectively. Nevertheless, these signals were characterized by a broad line width due to relative fast Xe exchange between the free and the transiently bound Xe state. 23% saturation effect could be observed at 0.1 μM protein concentration. Transfected HEK cells generated 13% CEST effect at a density of 0.2 million cells/mL but required the usage of isotopically enriched ^129^Xe to achieve sufficient sensitivity [418].

This initial study triggered interest in characterizing the Xe-bla interaction. X-ray crystallography, as well as its combination with protein mutagenesis and MD simulations revealed that bla crystallized as a tetramer with three cavities for Xe atoms (designated as Xe1, Xe2, and Xe3) being identified at similar sites in all four bla molecules [419]. Unsurprisingly, these include predominantly hydrophobic protein side chains leading to no water occupancy. The highest affinity was assigned to the Xe1 site with a binding constant of 40 M^−1^. HyerCEST was then helpful to investigate the structural dynamics of the Xe1 cavity in the presence of tazobactam as a competitive β-lactamase inhibitor [420]. Addition of this inhibitor caused line broadening indicative of accelerated Xe exchange under competition conditions. The lack of evidence in the crystal structure for an open pathway for Xe to reach the Xe1 cavity from the surface suggests dynamic fluctuations of the protein to enable access to the Xe1 cavity, that can actually host three Xe atoms.

Single-point mutations were then introduced to bla for perturbing the Xe1 affinity and exchange dynamics. The residues I263 and I279 regulate Xe access and were thus chosen for modifications. HyperCEST studies of bla mutants I263A and I279N revealed complete loss of CEST responses. Additional MD simulations and X-ray crystallography for single point mutations (I263A, I263N and I263L) illustrated that these can highly impact Xe access to the cavity and thus alter the achievable CEST contrast [421].

Another example of this Xe host category is the maltose-binding protein (MBP). Wild-type (WT) MBP binds maltose and other maltodextrins between two nearly symmetrical lobes that can undergo a transition from an “open” (MBP_open_) to “closed” (MBP_closed_) conformation [422]. The open state does not exhibit a HyperCEST response for bound Xe at 80 µM MBP concentration. This changes upon addition of 1 mM maltose where a response was observed at 95 ppm (Xe@MBP_closed_). The limit for maltose detection was in the range of 100 nM where the observable saturation contrast was still (5.0 ± 0.7)%. This could be improved to a detection limit of 32 nM by means of mutagenesis of Ile-329 to Tyr with a remaining saturation contrast of (7 ± 1)%. Switching to a cellular environment with expression of an MBP-GFP tag in *E. coli* did come with a CEST response of ~25% in the presence of maltose compared to ~5% saturation response in *E. coli* expressing MBP in the absence of maltose.

A joint use of both MBP (27 μM) and bla (80 μM) in the presence of maltose could thus demonstrate an example for the multiplexing approach that is achievable with HyperCEST detection. The signal at 60 ppm (Xe@bla) was maltose-independent whereas the one at 95 ppm (Xe@MBP) vanishes in the absence of maltose. This example might pave the way for in vivo quantification of maltose via a ratiometric evaluation of the responsive (MBP) and non-responsive (bla) HyperCEST signals. Rational mutagenesis could also yield MBP variants with a range of ^129^Xe NMR chemical shifts with applications in multiplexed cellular imaging [423].

### 5.11. Super Hosts

With the insights regarding the HyperCEST performance of individual functionalized Xe hosts or scaffolded approaches, there is an increasing interest in the field of ^129^Xe NMR/MRI to design host systems that can encapsulate and facilitate exchange of many Xe atoms at once to generate so-called “super host” systems. Such boosted encapsulation of Xe atoms leads to the creation of a higher contrast per particle unit. In other words, the product of Xe loading per host and of the exchange rate provides a fundamental measure of the achieved contrast amplification [123]. The HyperCEST contrast efficiency also depends on the exchange rate of Xe between the bound compartment and the bulk pool. Sometimes, the medium fast exchange rate observed for superhosts might promote the enhancement of their HyperCEST capabilities.

#### 5.11.1. Nanodroplets/Nanoemulsions

Regarding gas-binding substances, perfluorooctyl bromide (PFOB) nanodroplets are known as blood substitute as they solubilizes oxygen [424] and were also proposed as a carrier for intravenous delivery of hyperpolarized ^129^Xe [425]. These PFOB characteristics paved the way for generating highly sensitive ^129^Xe HyperCEST NMR/MRI reporters. In analogy to the compartmental lipoCEST approach, PFOB nanoemulsions encapsulate Xe and the surrounding free Xe in solution serves as the bulk pool. Usage of PFOB nanoemulsion droplets has two advantages: achieving a large number of Xe atoms dissolved within each droplet in addition to fast Xe exchange dynamics (compared to cryptophane hosts). This produces a strong contrast per reporter unit in analogy to lipoCEST. To obtain such reporters, PFOB was homogenized along with a poloxamer surfactant (Pluronic F-68) to yield perfluorocarbon-in-water nanoemulsions with narrow droplet size distributions (160–310 nm) [426]. The surfactant was shown to be effective in stabilizing the droplets which was revealed through the low polydispersity and slow growth curves, respectively. The droplets were about 130 nm in size at 1 h post synthesis. However, these droplets slowly grew over time due to slow coalescence after emulsification. The ^129^Xe HyperCEST measurements revealed two CEST peaks for PFOB, namely a broad one at (111 ± 9) ppm (Xe@PFOB) and at ~192 ppm (Xe@aq), respectively. With increasing droplet sizes, the Xe exchange rate is slowed down due to diffusion-governed Xe residence times in the droplets. As opposed to lipoCEST, PFOB nanodroplets displayed fast-intermediate chemical exchange for smaller (160 nm) droplets compared to slow-intermediate exchange for larger (310 nm) ones. Additionally, the droplets with 210 nm size were detectable as low as 1 pM while the bigger droplets (310 nm) were detected at 100 fM using saturation powers compatible with in vivo-compatible absorption rates (SAR). The slow-down of the Xe exchange rate will enhance contrast on a per agent basis for PFOB nanodroplets which might be achieved by utilizing different surfactants such as cross-linking phospholipids or fluorine-containing block copolymers [426].

The cell labeling properties of PFOB nanodroplets with defined size was evaluated in combination with a rigid macrocyclic Xe host such that the differently labeled cell populations can be delineated based on the distinct Xe chemical shifts from both hosts. This multichannel (multiplexing) approach could demonstrate the detection of different types of Xe hosts, i.e., PFOB (compartmentalization) and CryA-ma (inclusion complexes), to non-specifically label separate cell types. A fluorophore tag incorporated into the PFOB droplet (DiI) and another one conjugated to CryA-ma (CryA-FAM) was helpful in validating the MRI findings through fluorescence. Unlike fluorescence, Xe NMR has the additional advantage of differentiating between Xe residing in extracellular and cell-associated environments. The same cell line was utilized for both labelling protocols in order to observe the unaltered chemical shift difference (40 ppm) between the two Xe hosts and to avoid CEST peak broadening arising due to differences in relaxation effects and magnetic field inhomogeneity after the host’s cellular intake [353]. ^129^Xe HyperCEST spectra of mouse fibroblast cells (L929) incubated with 1.6 nM PFOB nanodroplets (diameter: 200 nm) for 18 h indicated a narrower PFOB-related CEST (cell associated) peak at 110 ppm and a Xe@PFOB peak (120 ppm) in solution. ^129^Xe-MRI of L929 (10 × 10^6^ cells/mL) cell suspensions labeled by using either PFOB nanodroplets (0.25 nM, 380 nm) or CryA-FAM (50 µM) indicated four different spin pools, namely Xe@solution (192 ppm), Xe@PFOB in cells (110 ppm), Xe@CryA-FAM in cells (70 ppm), and Xe@CryA-FAM in solution (60 ppm), respectively. Additionally, a higher HyperCEST effect was observed for L929 cells labeled using either PFOB nanodroplets (diameter: 380 nm) or CryA-FAM with a mean value of 70% while being saturated at the respective Xe host resonance in cells. Both Xe hosts also indicated a 10 ppm upfield shift upon their cellular internalization. Thus, the compartmentalization of Xe by PFOB guarantees sufficient Xe exchange depending upon its size for detecting molecular events in vivo at relatively low concentration (nM regime). To achieve this, the PFOB nanodroplets should be stabilized with more intact surfactants such that its size remain unchanged over time and while during an extended longitudinal study [14].

**Figure 12:**
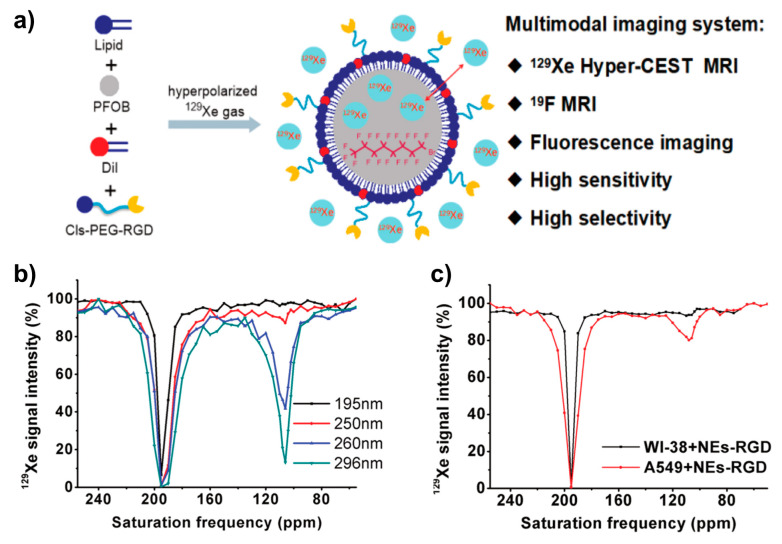
Application of PFOB nanodroplets as HyperCEST agents. (**a**) Sketch of nanodroplet structure. (**b**) z-spectra for different nanodroplet sizes. The CEST response increases with the droplet size. (**c**) CEST response from target vs. control cells incubated with functionalized nanodroplets. Adapted with permission from *ACS Appl. Bio Mater.* 2019, 2, 1, 27–32 [427]. Copyright (2019) American Chemical Society.

Such size-controlled PFOB nanoemulsion might foster the generation of multimodal contrast agents in combination with another reporter such that a biological target can be detected selectively and at high sensitivity. The intrinsic fluorine atoms along with the gas-binding properties of PFOB can be manipulated to generate multimodal contrast agents, e.g., ^129^Xe and ^19^F NMR/MRI. Stable PFOB nanoemulsions have been prepared by employing two emulsifiers such as Pluronic F-68 and Lipoid S75, respectively. The hydrodynamic diameters of PFOB nanoemulsion (NE) were 195 nm with an observed polydispersity index of 0.19. Introduction of a fluorescent tag (DiI) into the phospholipid surface of PFOB nanoemulsion via hydrophobic interaction rendered them suitable for fluorescence imaging [14]. Similarly, the targeting capabilities of PFOB nanoemulsion was demonstrated with the incorporation of a cholesterol-labeled targeting peptide (Cls-PEG-RGD, see Figure 12a) synthesized via click chemistry between mercapto and maleimide. This enables them to target cells expressing higher levels of α_v_β_3_. The targeted RGD-nanoemulsion (RGD-NE) displayed a slightly larger hydrodynamic diameter than untargeted NEs owing to the surface modification. Typically, a size control over the PFOB nanoemulsion was achieved by manipulating the ratio between PFOB and phospholipids, followed by processing the as-generated nanoemulsion either through sonication or by extruding the final nanoemulsion through appropriate membranes, respectively. Surprisingly, the fluorescence images of normal human lung cells (WI-38) and murine macrophage (RAW 264.7) indicated a higher uptake of NEs compared to negligible uptake by human lung cancer cells (A549). Thus, non-specific uptake by cells was prevalent in spite of tethering the PFOB with targeting modules on its surface. Conversely, the fluorescence of A549 cells indicated a higher uptake only in the case of RGD-NEs incubation. This was also indirectly proven through an RGD-induced blocking experiment.

The ^129^Xe HyperCEST spectrum indicated a peak at 106 ppm from the PFOB-dissolved Xe for NEs only if their particle size is larger than 250 nm and concentration is close to 5 pM. Similarly, the A549 cells incubated with RGD-NEs displayed a specific CEST response centered at 106 ppm compared to no response from WI-38 cells (see Figure 12b,c). Additionally, ^129^Xe MRI of A549 cells treated with RGD-NEs were localized with 54% HyperCEST effect compared to the significantly less efficient, non-specific uptake of NE by WI-38 cells (3% HyperCEST effect) and that of NE by A549 cells (17% HyperCEST effect), respectively. Furthermore, ^19^F MRI also demonstrated the targeting achieved through RGD-NEs upon incubation with A549 cells, thereby pushing the detection limit as low as 26 pM. ^19^F MRI of mice injected intravenously with RGD-NEs was not linked to any acute toxicity and the PFOB nanodroplets were found to accumulate in the liver after systemic circulation. Intratumoral injection of RGD-NEs to an A549 xenograft revealed a hot spot ^19^F image and the location of the tumor was shown by comparing it to previously acquired anatomical images. This study implies the advantage of using multimodal probes in targeting different cancerous cells and tumors. Further, non-specific uptake by cells was prevalent in spite of tethering the PFOB with targeting modules on its surface [427]. However, different in vivo aspects, e.g., stability and performance of such probes must be evaluated in an elaborate manner prior to further translation [427].

Although PFOB is beneficial as Xe super host, it suffers from displaying variable particle sizes over a period of time [426]. This might hinder its application in which longitudinal studies are desired. Along this line, PFOB nanodroplets were alternatively stabilized in a recent study with equimolar amounts of purified egg lecithin (E80 S) and a semi-fluorinated alkane diblock compound (C_6_F_13_C_10_H_21_, F_6_H_10_) after high-pressure/shear homogenization (1000 bar, 30 min). This led to generation of particles with (209 ± 17) nm. Incubating the as-generated PFOB nanodroplets with human blood samples from patients with heart disease and healthy volunteers indicated that these droplets are specifically and immediately taken up by monocytes/macrophages, followed by deposition in reticuloendothelial (RES) systems, e.g., liver and spleen.

It should be mentioned that PFOB nanodroplets were also utilized as contrast agents for achieving targeted ^19^F MR imaging of inflammation [428,429]. The half-life of PFOB was reported to be 12 days and it was reported that it is exhaled through the lung [424,429]. However, ^19^F MRI of such compounds relies on the detection of (a) thermally polarized spins and (b) does not benefit from CEST enhancement. It is thus significantly less efficient than the HyperCEST approach as demonstrated in the above mentioned recent study with micelles carrying a high local Xe host load [351].

#### 5.11.2. Bacterial Gas Vesicle

Even though PFOB fosters itself as Xe super host despite its size-limited contrast enhancement, still there is a strong interest in improving the performance of HyperCEST agents. New types of super hosts might be generated either synthetically or through biogenic technology, albeit preferably without elaborative steps and extensive purification, respectively. Indeed, genetically encoded Xe hosts produced via the biogenic strategy can display somewhat superiority over PFOB nanodroplets in terms of Xe cargo capacity: bacterial gas vesicles provide a loading volume of 4.1–16.5 × 10^9^ Å^3^ compared to PFOB with ~4.2 × 10^9^ Å^3^ ascribed to its droplets with ca. 200 nm [14].

Gas vesicles (GVs) are gas-filled protein-shelled intracellular nano compartments that are mostly produced by different water-borne microorganisms to foster buoyancy such that the latter could migrate to the surface in search of light and nutrients [430,431]. GVs provide buoyancy to cells once a fraction of the cell volume (about 3–10%) is occupied by gas [432,433]. Such GVs are produced to display different functions like gas diffusion/permeation across the proteinaceous shell, precluding water vapor condensation inside its hollow cavity, and avoiding its collapse under varying hydrostatic pressures experienced at different depths, respectively [434]. In spite of having only 20 Å thick proteinaceous shells on average, GVs are found to resist collapse to a considerable extent. The hollow GVs are usually filled with air of atmospheric composition and it is in constant equilibrium with gas dissolved in the aqueous phase. Water vapor does not condensate inside the GVs even though the vesicle wall is permeable to different gases and molecules, e.g., H_2_, N_2_, O_2_, Ar, CO, CO_2_, and perfluorocyclobutane etc. This observation was believed to occur due to a highly hydrophobic and tightly curved inner face of the gas vesicles without any suitable nucleation sites [430,431]. Mature GVs are either spindle- or cylinder-shaped and their dimensions are typically 0.1~2 µm (length) by 45~250 nm (width), respectively [435]. The most widely studied GVs are namely from cyanobacterium *Anabaena flos-aquae* and the haloarchaea *Halobacterium salinarum* and *Haloferax mediterranei*. The carbohydrates produced by photosynthetic bacteria are considerably denser than water and might serve as a ballast. The counteractive effects of GVs (buoyancy) and carbohydrate ballast enable diurnal vertical migrations of the bacteria [435]. The vesicle shells are generated almost exclusively from 7 to 8 kDa GV protein A (GvpA) which is not cross-linked or otherwise posttranslationally modified [436]. The GvpA units are assembled into a low-pitch helix and it is highly hydrophobic and negatively charged at neutral pH in addition to possessing a highly conserved sequence at its structure [437,438]. There are characteristic 4.6 nm striations or ‘ribs’ of GvpA which are available perpendicular to the long axis of the gas vesicles. Additionally, GvpC is well characterized (3 times larger than GvpA) and it adheres to the outside of the shell formed by GvpA thereby strengthening the overall structure [439,440]. Further GV proteins such as GvpF, GvpG, GvpJ, GvpL, and GvpM were detected through immunoblotting. They represent less than 1% of overall GV protein in vesicles isolated from *Halobacterium salinarum*. The GvpJ and GvpM display partial sequence similarity to GvpA and are hypothesized to be part of cap formation while GvpF, GvpL and GvpG are hypothesized to be linked to nucleation of the gas vesicle assembly process at the tips of the caps [441].

The acoustic impedance mismatch occurring between the gas interior of GVs with surrounding aqueous media makes the GVs to strongly scatter sound waves, a prerequisite for using them as ultrasound (US) contrast agents as their initial imaging application [255,442,443,444,445]. These GVs generate robust US contrast across a range of frequencies at pM concentrations and also exhibit harmonic scattering that enable enhanced detection without any background in vivo [255]. In native bacteria or archaea, the gas vesicles are encoded usually by clusters of 8 to 14 genes including one or two primary structural proteins and other genes encoding the putative assembly factors or minor shell constituents. A heterogeneous expression of the GVs as acoustic reporter genes for US was attempted in another bacterial species in order to generate microbial-based diagnostic and therapeutic agents [446]. Only the expression of an *E. coli*-compatible gene cluster from *Bacillus megaterium* aided the translation of GV-forming genes from cyanobacteria to *E. coli* [447]. This illustrates the emerging use of GVs and renders them interesting for Xe MRI applications. In fact, *E. coli* and *Salmonella typhimurium* expressing the above discussed acoustic reporter genes was imaged through US detection at concentrations on the order of 10^8^ cells mL^−1^ that corresponded to volume fraction of approximately 0.01%. This enabled in vivo visualization of GVs in the gastrointestinal (GI) tract and tumor xenografts, respectively. Further, the GV protein (GvpC) was utilized as a platform for molecular engineering of GV-based US contrast agents. Deletion, addition or modification of GvpC resulted in tunability of acoustic, harmonic, mechanical, zeta potential and ligand tagging at GVs produced in *Anabaena flos-aquae* [442]. Such work illustrates the engineering options that might also be relevant for GV detection with other modalities.

GVs have thus been investigated for ^129^Xe HyperCEST NMR/MRI. Although earlier reports imply weak non-specific interactions of hyperpolarized ^129^Xe with several different proteins’ cavities and voids, the genetically encoded GVs come with a better tunability of their structure (e.g., shape and size), thereby making GVs a promising class of ^129^Xe HyperCEST contrast agents. It was hypothesized that dissolved ^129^Xe might partition into the GVs to produce a distinct chemical shift at gaseous phase followed by efficient exchange between GVs and solution. This motivates the establishment of genetically encoded ^129^Xe HyperCEST reporters. GVs from *Anabaena flos-aquae* were isolated via hypertonic lysis followed by purification aided by centrifugally assisted flotation [59]. The generated GVs displayed approximately 145 nm as diameter and lengths ranging between 250 to 1000 nm, respectively. ^129^Xe HyperCEST NMR of GVs indicated a CEST peak at 31.2 ppm in addition to free Xe in aqueous solution with a peak at 195 ppm, respectively. The broadening of Xe@aq peak is a characteristic of CEST detection which was observed earlier with fast exchanging ^1^H CEST agents [448,449]. The GVs (400 pM) saturated at different saturation times (*t*_sat_ = 0.4, 0.8 s) indicated saturation contrasts of (16 ± 2)% and (33 ± 2)%, respectively. However, a statistically significant contrast was achieved at 25 pM of GVs saturated for 1.63 s. Similarly, ^129^Xe HyperCEST MRI of GVs (400 pM) revealed a nearly complete saturation and a significant saturation contrast was also observed at lower GVs concentration (100 pM).

The multiplexing aspect was demonstrated by ^129^Xe HyperCEST NMR of intact *Halobacteria* sp. NRC-1, *Microcystis* sp. and *E. coli* suspended in the appropriate media. Except for the first two species, the *E. coli* was transformed with a minimal GV-forming gene cluster from *Bacillus megaterium* [447] in order to induce the host to produce GVs. The CEST peaks for *Halobacteria* sp. NRC-1, *Microcystis* sp. and *E. coli* were observed at 14.4 ppm, 30.6 ppm and 51.4 ppm, respectively. Additionally, GVs were also utilized as quantitative reporters of gene expression by placing its expression in *E. coli* under the control of a promoter induced by isopropyl β-D-1-thiogalactopyranoside (IPTG). An increased GV expression was noted in IPTG-induced cells resulting in a robust HyperCEST image contrast compared to no contrast from non-induced cells or induced cells that contained a control vector lacking GV genes, respectively. The magnitude of HyperCEST contrast was highly dependent on the dosage of IPTG administered, thus confirming the capability of GVs as quantitative gene expression reporters.

**Figure 13:**
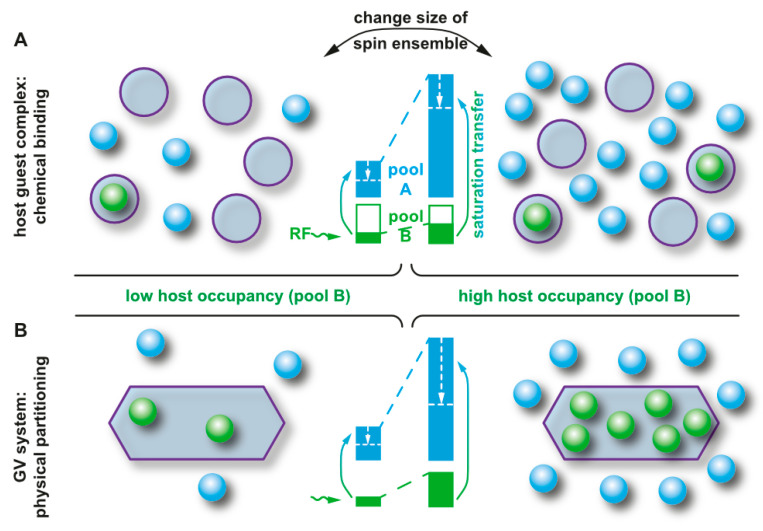
Self-adjusting filling of Xe hosts. (**A**) Chemical affinity of Xe to CrA: The number of loaded hosts does not scale linearly with increasing the total amount of Xe in the solution. (**B**) Physical partitioning into the GV cavity: The GVs are “elastically” loaded with a number of Xe atoms that reflects the size of the dissolved Xe pool according to Henry’s law. Reprinted with permission from Kunth et al. ACS Nano 2018, 12, 10939–10948 [133]. Copyright (2018) American Chemical Society.

An important aspect is that the excellent CEST performance of GVs at lower concentrations (pM range) relies on simple physical partitioning of ^129^Xe. This provides an “elasticity” of their loading as illustrated in Figure 13. The amount of Xe inside the GVs follows the surrounding Xe concentration in a self-adjusting way and thus allows for a stable CEST contrast. This was investigated in detail using quantitative HyperCEST (qHyperCEST), a method which will be explained in more detail in Section 6. This partitioning makes optimum use of the available magnetization. Unlike conventional CEST agents, the CEST pool self-adjusts (according to the ideal gas law) and does not become less efficient with increasing bulk pool size.

GVs could be functionalized with appropriate targeting moieties (e.g., antibodies) in order to generate targeted GVs-based Xe NMR contrast agents for imaging a specific biological target. For example, the GVs isolated from *A. flos-aquae (Ana)* were functionalized with biotin via sulfo N-hydroxysuccinimide (sulfo-NHS) linkages to lysine side chains on the GV surface [59]. *Ana* GVs were chosen for functionalization since they are highly resistant to collapse and allow easy handling during repeated purifications compared to GVs produced from other species. These biotinylated GVs were combined with streptavidin-functionalized antibodies targeting the HER2 receptor via a modular approach involving streptavidin-biotin binding. ^129^Xe HyperCEST MRI of HER2-expressing breast cancer cell line SKBR3 labeled with anti-HER2 GVs indicated a prominent saturation contrast of (79 ± 1)% compared to almost no contrast observed in the control Jurkat cells [450]. Modification of GVs was also achieved in different contexts for

- creating theranostic GVs for both US-imaging and photodynamic therapy (PDT) agent addressing human breast carcinoma (MCF-7) [451];
- obtaining a genetically engineered variant of *Ana* GvpC-containing N- or C-terminal hexahistidine sequences in *E. coli* for protein fusion with lysine-rich protein (LRP). The latter introduces 100 positive charges that modulate the GVs behavior in solution and in vivo [452];
- expressing GvpC_RGD_ on their surface leading to specific targeting of integrin-overexpressing U87 human glioblastoma cell line in vitro compared to no uptake in the case of scrambled GvpC_RDG_, respectively [453];
- uptake by phagocytic cells through polycationic polyarginine (R8) peptide fusion [454];
- a modular approach by fusing GvpC with SpyTag (ST; a 13-residue peptide that forms a covalent amide bond with a partner SpyCatcher protein under physiological conditions [455] for demonstrating such the capability of such GVs (SpyTag-SpyCatcher) as multimodal probes (e.g., acoustic and fluorescence imaging) [442].

Overall, the GVs can be functionalized either by chemically modifying their surface amino acid residues or by overlaying the genetically engineered protein GvpC on the GVs surface in order to attain modular way of functionalization via protein fusions onto the GvpC, respectively.

It should be mentioned that beside GV applications in Xe MRI, the gas contents inside the GVs are shown to produce microscale magnetic field gradients in their vicinity and subsequently dephasing the aqueous protons leading to contrast in *T*_2_-weighted and quantitative susceptibility images [456]. Interestingly, the MR contrast generated by susceptibility measurements of GVs can be modulated using ultrasound (US) in order to avoid background interference. Additionally, the acoustic properties of GVs were utilized in ^1^H MRI where the serial acoustic collapse of GVs engineered with certain collapse thresholds were performed to achieve multiplexed imaging [445]. This option could also be included in Xe MRI detection protocols.

## 6. Quantitative Analysis of ^129^Xe Host Systems

In many studies, NMR and MRI are used as analytical tools where differences in signal intensities are observed but not necessarily analyzed to obtain absolute numbers for certain physical or chemical quantities. While this qualitative interpretation has been sufficient for many applications, there is an increasing interest in evaluating certain characteristic parameters on the molecular level to compare signal differences in different samples and between different setups.

In this context, computational efforts have been made for quantitative analysis of CEST responses. The saturation transfer comes with the advantage that the signal contrast can be controlled through the saturation duration (*t*_sat_) and power (*B*_1_). It is thus possible to characterize the exchanging host-guest system by applying different saturation conditions and analyzing the spectral response. The time-dependent magnetization components for two exchange-connected pools *A* and *B* that are exposed to a saturation pulse are described by the Bloch-McConnell equations [113]. Key parameters are the pool size of bound nuclei, *f_B_*, the exchange rate *k_BA_*, and the intrinsic relaxation times *T*_1/2_ in both pools. The binding constant *K*_B_ can be derived from *f_B_* for a given host concentration or, knowing the exchange kinetics and binding properties, the concentration of a host can be determined.

### 6.1. Quantitative hp Xe Saturation Transfer Analysis (qHyperCEST) for Host Characterization

**Figure 14:**
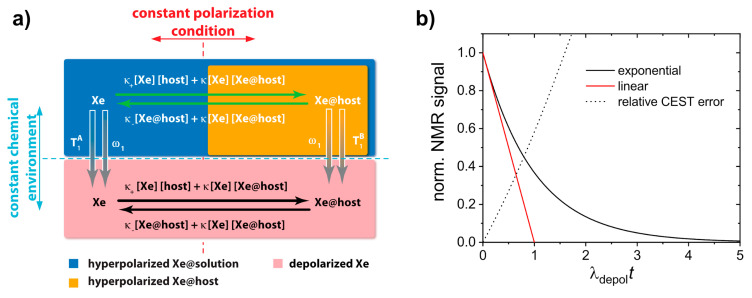
Aspects of qHyperCEST analysis. (**a**) Exchange-connected spin pools and depolarization pathways evaluated in qHyperCEST. Adapted from Supp. Inf. of [76]. (**b**) Exponentially driven depolarization given by the depolarization parameter λ_depol_ and applied saturation time. For CEST effects <25–30%, the behavior can be approximated by a linear slope and the relative CEST error remains below 20%.

For CEST with thermally polarized protons, an unambiguous analysis of the saturation response is somewhat challenging as a certain CEST signal can originate either from a small pool *B* with a high exchange rate or a large pool *B* with slow exchange. Different approaches have been implemented to resolve this issue [457,458,459,460]. For hp Xe, however, it has been shown that a simplified approach is possible that eventually yields the full HyperCEST solution (FHC, [353]). The model takes both the intrinsic and the RF-driven depolarization into account as well as the chemical exchange pathways that are indifferent to the magnetization of the nuclei (see Figure 14a).

The theoretical background has been validated with experimental data [76] and illustrated nicely that carefully acquired *z*-spectra with high reproducibility of Xe magnetization perfectly confirmed the predicted exponential Lorentzian line shape of individual CEST responses. The exponential nature of the increasing CEST response can make it difficult to compare z-spectra from samples with different host concentrations. The important parameter is the product of applied saturation time and depolarization rate (λ_depol_). The latter one includes host concentration and exchange rate and moderate CEST responses not exceeding 25–30% of signal loss can be approximated linearly by the product λ_depol_*t* (see Figure 14b). Within this regime, this allows rough estimates for relative differences in host concentrations or exchange rates or to predict the impact of scaling the applied saturation time.

Deriving the quantitative exchange parameters from the FHC-based evaluation was then used to compare CryA with CB6 to quantify the better gas turnover rate given for the more open CB6 system [123]. It made clear that the transition to faster exchanging systems makes direct detection even more challenging and that some host systems might be overseen as valuable systems unless a HyperCEST experiment is performed. Moreover, the optimum performance only becomes clear once the saturation parameters are adjusted: slow exchanging systems can perform better with low saturation power but are actually outcompeted by fast exchanging systems if higher saturation power is permitted (limitations might be given by the coil hardware or the maximum allowed rf power deposition in biomedical applications). Eventually, the quantification of the Xe release rate from the host into the bulk pool in combination with the fraction of occupied hosts (derived from the binding constant) allows to classify HyperCEST agents according to their gas turnover rate as mentioned in Section 4.2. A similar quantification was used to investigate the Xe loading of GVs. This revealed that adjusting the partial Xe gas pressure on top of the sample solution causes a directly proportional loading of GVs with Xe spins that follows the ideal gas law for these nano-compartments [133].

Along the line with performing quantitative HyperCEST measurements, some general rules could be derived for the optimum cw saturation once the exchange parameters are known [122]. A pulse strength corresponding to *B*_1_ = 5 *k_BA_*/γ (with the gyromagnetic ratio of ^129^Xe, γ) was found to generate 96% of the maximal HyperCEST contrast while preserving the spectral selectivity. The latter aspect is important for setups where more than one Xe host shall be detected [14] because further increase of the saturation power simply yields additional line broadening in the *z*-spectra.

CEST detection is only one implementation of perturbing one of the exchange-connected pools and observing the signal response in the other one. Another approach is the selective inversion of the bulk pool, followed by observation of the bound Xe pool. This technique has actually revealed a component for degenerate Xe exchange with CryA in water under certain conditions [461] (i.e., one Xe atom kicks out another from an already occupied host). The degenerate type of exchange had already been postulated in an earlier study [87], albeit in an organic solvent. A combination of different detection schemes at variable Xe concentration allowed the identification of exchange mechanisms and the quantification of their individual contributions to the overall kinetics [462].

### 6.2. Quantifying HyperCEST Changes in Displacement Assays

Quantitative investigation of HyperCEST signals becomes particularly interesting when following the progress of a chemical reaction. Typically, NMR is too slow for most cases but an enzymatically activated conversion can extend over several minutes. Still, conventional (Hyper)CEST is a rather slow method with point-wise acquisition of the data along the *z*-spectrum. In its original implementation, this did require a fresh Xe delivery with low shot-to-shot noise for each data point [58]. A homogeneous sample, however, permits to pair the otherwise constant resonance condition along one spatial dimension with a saturation pulse in the presence of a magnetic field gradient along that dimension. Thus, the CEST effect can only occur for those hosts that have the matching resonance condition for trapped Xe in the presence of the gradient. This principle has been applied by different labs in order to accelerate CEST acquisitions for ^1^H [463,464] and for ^129^Xe [465,466]. A temporal resolution of ~30 s can then be used to follow the change in a HyperCEST response where the host is increasingly blocked. An application example is the production of cadaverine from lysine by lysine decarboxylase (LDC) where the starting material has a low affinity for the host CB6 whereas the product displaces Xe and eventually causes a complete loss of the HyperCEST signal [124]. The time-resolved HyperCEST monitoring of this reaction evaluated the *z*-spectra in the context of the observed exponential Lorentzian line shape [76]. Thus, calculating the HyperCEST intensity from the peak center after logarithmic rescaling yields a value that is directly proportional to the amount of accessible CB6 [467]. Its decrease over time reflects the ongoing production of cadaverine and yields a specific activity for LDC that agrees well with fluorescence measurements that also rely on displacement assays with CBs [468]. Other types of catalytic conversion have also been studied with ^1^H NMR using the so-called catalyCEST approach [469]. The advantage of qHyperCEST is its very high sensitivity that enables following the process just at the very beginning when the conversion rate is still constant. Changes in catalyCEST signals, however, only become detectable after a larger amount of substrate is converted.

## 7. Translation Potential

NMR with hp ^129^Xe has been long established for in vitro studies in the material sciences and through demonstrations of novel Xe biosensors. Regarding future applications of functionalized hp Xe in such sensors, biomedical studies in a preclinical context are the clear focus. This has also been the case for DNP and PHIP/SABRE applications, particularly for studies with ^13^C-labelled pyruvate since the advent of dissolution DNP [470] and its continuous transition into clinical studies [72]. The DNP technique has been widely used to polarize ^13^C or ^15^N found in metabolites such that the change in metabolic activity linked to different diseases such as cancer or multiple sclerosis can be easily identified. A rather fast translation of DNP-mediated ^13^C-metabolic findings into clinics was facilitated through continuous development of improved polarization of ^13^C-labelled pyruvate, MRI optimizations, disease models, and biological insights achieved via various preclinical investigations. In spite of having several practical limitations, the recent advancement on DNP led to successful clinical trials, e.g., [1-^13^C]-pyruvate trial in prostate cancer patients [72,471] and its further translation into clinical routine [472,473].

A critical point for the translation of the HyperCEST technique to (pre-)clinical applications is that this technique requires a certain time frame for building up the actively driven signal loss. A key component for future in vivo studies is thus the incorporation of host structures that provide a rather efficient exchange for optimum CEST effect. Additionally, clinical experience with MRI of dissolved Xe beyond lung tissue is still an emerging field of research that leaves room for improvement. Achieving sufficient SNR for the ^129^Xe signal in different tissues is constrained by the initial level of hyperpolarization obtained by spin-exchange optical pumping. Although improvements in spin-exchange optical pumping setups and ^129^Xe bio-carriers might overcome these limitations, a successful demonstration of the Xe biosensor imaging at the desirable low concentrations in a pre-clinical setting is still lacking.

**Figure 15:**
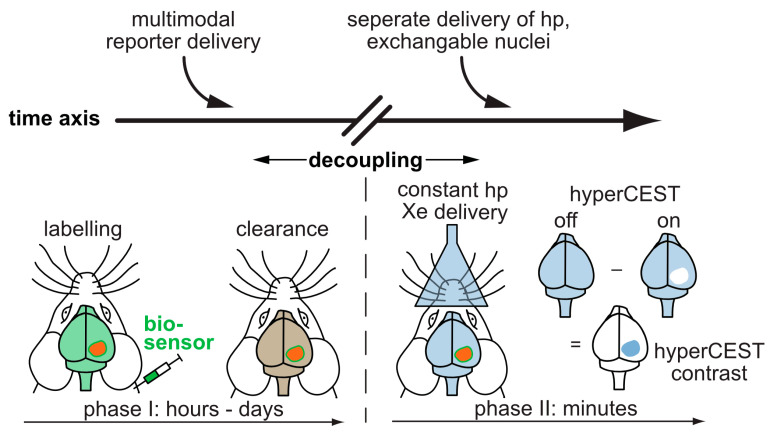
Translation of functionalized Xe to in vivo studies. The sensor is applied systemically and can accumulate in target tissue while wash-out occurs in the surrounding tissue. The decoupling of the time scales allows to perform the more time-sensitive detection of hp ^129^Xe in a second step. The hp ^129^Xe can be delivered via inhalation and visualized in other highly perfused organs such as the brain.

In preclinical studies, hp ^129^Xe MRI has been utilized to validate different lung disease models, e.g., radiation induced lung injury (RILI), fibrotic lung tissue, elastic models of emphysema, asthma models, brain perfusion etc., noninvasively compared to tissue histology. By utilizing the sensitivity provided by hp ^129^Xe MRI, the progress or changes in the disease patterns are monitored longitudinally in a more controlled way. Clinical lung imaging with Xe is already quite established and provides great anatomical and functional information [474,475]. It also served as a stepping stone for advanced clinical applications for mapping the Xe distribution in other organs like the human brain [69,476,477] as well as Xe MRI of the kidneys [70]. Additionally, hp ^129^Xe MRI in the clinical side faces challenges similar to that of hp ^13^C MRI, e.g., requiring optimization of dedicated RF coils for signal transmission/reception. Further, to sustain a longer T_1_ time and polarization levels, the interaction of hp ^129^Xe with oxygen during delivery should be minimal. A vast majority of clinical research is performed on asthma and COPD disease models using hp ^129^Xe MRI [2] where Xe has no extended contact time with oxygen within the blood or organs and MRI with Xe dissolved in such tissue is still being explored. A critical contribution is the parallel improvement of the technical components such as modern polarizers including high-performance laser diodes [478], optimized RF antenna systems [479,480], and adapted readout protocols. Any Xe MRI application benefits from such advancements, irrespective of being void space imaging or dissolved gas detection that ultimately also enables (pre)clinical biosensor studies. Recently, hp ^129^Xe was utilized for the first time to quantify the gas exchange defects in a rat model of pulmonary hypertension [481]. Similarly, another (pre)clinical study reported the in vivo observation of ^129^Xe HyperCEST responses after administering Sprague-Dawley (SD) rates with unfunctionalized CB[6] at millimolar concentrations [482]. Although this preliminary study provided insights on possible Xe distribution in highly perfused areas and nonspecific CB[6] accumulation in different organs, still there is a need for further improvements, e.g., MR sequence optimization, Xe carrier functionalization etc. to facilitate faster clinical translation. Such preclinical approaches might lead to testing different ^129^Xe biosensors in vivo subsequently and to moving forward to the demonstration of molecular imaging applications of such probes. The full potential of hp ^129^Xe NMR/MRI is not yet realized in the clinical stage due to an ongoing accumulation of sufficient and profound translation knowledge related to several components, including organ perfusion with Xe, polarizer optimization for CEST applications, biosafety of biosensors, pharmacokinetics etc. Critically, this still happens within a relatively small research community. In a clinical context, Xe was originally seen as an anesthetic or neuroprotective agent and also partly as a non-specific gas interacting with different endogenous lipidic (hydrophobic) components in vivo. Currently, there is not yet a large number of labs using hp Xe in a clinical context, either because they have to buy the polarizer as a non-standard equipment or because they need expertise to build it. Such a prevailing situation is also detrimental for its quick translation into the clinics. Further, to expedite easy translation of hp ^129^Xe MRI into (pre-)clinical settings, the cost linked to Xe production/enrichment, hyperpolarization, and polarizer hardware should be reduced such that many researchers, clinicians, and patients will appreciate the usefulness of this technique. However, hp ^129^Xe MRI might serve as a complementary technique in dual-modality studies by providing valuable insights at lower concentration (nM/pM), e.g., ^129^Xe NMR/optical imaging, ^129^Xe/^19^F NMR etc. similar to other imaging modalities. According to the pharmacokinetic simulations established by Shapiro and co-workers, translatability of ^129^Xe HyperCEST technique into the (pre-)clinics should be achievable [450]. However, a main challenge in this context is the requirement of highly interdisciplinary effort. Compared to the other hp NMR techniques, the big advantage of Xe biosensors is the separate delivery of functionalized host units and hp nuclei as discussed in Section 3.2. Figure 15 illustrates how this decoupling of the time scales for sensor delivery and hp lifetime immensely broadens the applications of hp MRI beyond metabolic imaging and will remain a driving force for further Xe biosensor development. The convenient timing for defining a diagnostic window as in Section 3.2 together with simulations for highly efficient HyperCEST agents [450] pinpoint the way for diagnostic applications of functionalized Xe.

Overall, we also assume the clinical translation is less rapid than for DNP because the currently active groups do not cover the entire range of academic work comprising sensor synthesis, experimental SEOP handling, MRI expertise, intriguing animal models etc. all on one site.

## 8. Conclusions

The applications of hp ^129^Xe nowadays go far beyond gas phase NMR/MRI. Since the pioneering work undertaken at UC Berkeley almost 20 years ago, the Xe biosensor concept has been constantly expanding. Advancements from Xe MRI in general (driven by lung imaging applications) and the overall success of the CEST approach in MRI have made important contributions to further pursue this diagnostic approach. The community is small but making important progress, as illustrated during regular meetings like the XeMat conference [483]. A translation to in vivo applications will most likely happen with one of the more recent super hosts or scaffolded approaches to ensure most efficient CEST build-up under more challenging in vivo conditions where accelerated relaxation and blood perfusion will partially diminish CEST efficiency. Nevertheless, the feasibility of this approach has been predicted in a pharmacokinetic model with repeated Xe inhalation and low flip angle excitations for continuous imaging and on-resonance saturation of Xe bound to the above mentioned GVs [450]. Other host systems of similar efficiency should be investigated too. Neighboring disciplines like chemical engineering are invited to further promote this concept. At the same time, the sensing options with reversibly bound, hp ^129^Xe could make novel contributions to characterize emerging materials like MOPs/MOFs or discover weak interactions in supramolecular systems. Taken together, the perspectives for detecting “functionalized Xe” with the HyperCEST method are promising for interdisciplinary work. Its high sensitivity can address previously inaccessible molecular systems, and this will continue to make NMR and MRI important analytical tools throughout the natural sciences and biomedical research communities.
